# The Pet127 protein is a mitochondrial 5′-to-3′ exoribonuclease from the PD-(D/E)XK superfamily involved in RNA maturation and intron degradation in yeasts

**DOI:** 10.1261/rna.079083.121

**Published:** 2022-05

**Authors:** Karolina Łabędzka-Dmoch, Michal Rażew, Marta Gapińska, Jakub Piątkowski, Adam Kolondra, Hanna Salmonowicz, Joanna M. Wenda, Marcin Nowotny, Paweł Golik

**Affiliations:** 1 Institute of Genetics and Biotechnology, Faculty of Biology, University of Warsaw, Warsaw 02-106, Poland; 2 Laboratory of Protein Structure, International Institute of Molecular and Cell Biology, Warsaw 02-109, Poland; 3 Laboratory of Metabolic Quality Control, IMOL, Polish Academy of Sciences, Warsaw 00-783, Poland; 4 Institute of Biochemistry and Biophysics, Polish Academy of Sciences, Warsaw 02-106, Poland

**Keywords:** Pet127, exoribonuclease, mitochondria, *Candida albicans*, introns, RNA degradation

## Abstract

Pet127 is a mitochondrial protein found in multiple eukaryotic lineages, but absent from several taxa, including plants and animals. Distant homology suggests that it belongs to the divergent PD-(D/E)XK superfamily which includes various nucleases and related proteins. Earlier yeast genetics experiments suggest that it plays a nonessential role in RNA degradation and 5′ end processing. Our phylogenetic analysis suggests that it is a primordial eukaryotic invention that was retained in diverse groups, and independently lost several times in the evolution of other organisms. We demonstrate for the first time that the fungal Pet127 protein in vitro is a processive 5′-to-3′ exoribonuclease capable of digesting various substrates in a sequence nonspecific manner. Mutations in conserved residues essential in the PD-(D/E)XK superfamily active site abolish the activity of Pet127. Deletion of the *PET127* gene in the pathogenic yeast *Candida albicans* results in a moderate increase in the steady-state levels of several transcripts and in accumulation of unspliced precursors and intronic sequences of three introns. Mutations in the active site residues result in a phenotype identical to that of the deletant, confirming that the exoribonuclease activity is related to the physiological role of the Pet127 protein. Pet127 activity is, however, not essential for maintaining the mitochondrial respiratory activity in *C. albicans*.

## INTRODUCTION

Mechanisms that assure the presence of correctly processed RNA molecules in tightly regulated quantities are a central feature of gene expression. Pathways that shape the transcriptome show great evolutionary diversity, yet they share the common theme of interplay between transcription and RNA degradation. In general, transcriptional activity in the known genomes extends beyond the annotated gene sequences, often covering the majority of the genome (Kapranov et al. 2007a,b; Wade and Grainger 2014; Tudek et al. 2015; Łabędzka-Dmoch et al. 2021). Intronic RNAs, as well as transcripts originating from noncoding intergenic sequences are quickly degraded by diverse ribonucleases.

Mitochondrial genes are transcribed as polycistronic primary transcripts (from two to dozens, depending on the species) that undergo extensive processing (Ojala et al. 1981; Lipinski et al. 2010; Turk et al. 2013; Kolondra et al. 2015; Łabędzka-Dmoch et al. 2021) by simple single-subunit polymerases of bacteriophage origin (Masters et al. 1987). In wild-type *Candida albicans,* there are eight primary transcripts transcribed from separate (but similar) promoters (Kolondra et al. 2015). Transcripts corresponding to the entire mtDNA sequence, including intergenic regions, can however be observed in RNA degradation impaired strains (Łabędzka-Dmoch et al. 2021), suggesting pervasive transcription encompassing intergenic regions. RNA degradation is also responsible for the varying final abundance of mitochondrial RNAs originating from a single primary transcript (Mercer et al. 2011; Turk et al. 2013; Kolondra et al. 2015; Shang et al. 2018; Łabędzka-Dmoch et al. 2021).

The enzymatic activities involved in RNA degradation include diverse ribonucleases (Houseley and Tollervey 2009), with exoribonucleases playing the major role in mitochondria (Szczesny et al. 2012). The main ribonuclease of mitochondria is the mtEXO (mitochondrial degradosome) complex, exhibiting a 3′-to-5′ exoribonucleolytic activity that is composed of the conserved Suv3 helicase, and either the hydrolytic Dss1 RNase in fungi (Dziembowski et al. 2003; Hoffmann et al. 2008) and in trypanosomes (Mattiacio and Read 2008), or the phosphorolytic polynucleotide phosphorylase (PNPase) in animals (Borowski et al. 2013) and plants (Holec et al. 2006). This activity is essential for the functioning of the organellar gene expression system, and its dysfunction results in the loss of respiratory capacity in *S. cerevisiae* (Dmochowska et al. 1995; Golik et al. 1995; Rogowska et al. 2006), *S. pombe* (Hoffmann et al. 2008), and *C. albicans* (Łabędzka-Dmoch et al. 2021). The yeast mtEXO complex shapes the 3′ ends of protein coding transcripts that are protected from further degradation by the Rmd9 protein (Hillen et al. 2021), and degrades a variety of junk RNAs (Dziembowski et al. 2003; Rogowska et al. 2006; Łabędzka-Dmoch et al. 2021). In mammalian mitochondria it is also responsible for RNA degradation and surveillance (Szczesny et al. 2010; Dhir et al. 2018; Pietras et al. 2018a,b). The structural and enzymatic aspects of mtEXO activity have been extensively studied in yeast (Malecki et al. 2007; Razew et al. 2018).

In contrast, the nature and function of the mitochondrial 5′-to-3′ exoribonuclease activity, related to the Pet127 protein is not as well understood. A loss-of-function mutation of the *S. cerevisiae PET127* gene was found in a screen for suppressors of impaired mitochondrial mRNA-specific translation (Haffter and Fox 1992). Subsequent genetic and molecular studies indicated that Pet127p is involved in the processing of the 5′ termini of mitochondrial transcripts (Wiesenberger and Fox 1997; Chen et al. 1999; Islas-Osuna et al. 2002; Fekete et al. 2008). Overexpression of the Pet127p can partially suppress the phenotype related to the dysfunction of the mtEXO exoribonuclease complex (Wegierski et al. 1998). Involvement of the Pet127 protein in RNA degradation was also demonstrated in *S. pombe* (Wiesenberger et al. 2007). Recently, *S. cerevisiae* Pet127p was shown to be a negative regulator of the mitochondrial RNA polymerase Rpo41p through protein–protein interactions that do not depend on Pet127p ribonuclease activity (Corbi and Amon 2021).

In spite of these diverse functions, deletion of *PET127* in laboratory strains of *S. cerevisiae* does not lead to a loss of respiratory competence (Haffter and Fox 1992). Likewise, in *S. pombe* Pet127p is not essential for respiratory competence, and its dysfunction has only a moderate phenotype at the elevated temperature (Wiesenberger et al. 2007). In contrast, overexpression of the *S. cerevisiae PET127* gene from a strong *ADC1* promoter leads to respiratory failure and loss of functional (*rho*^+^) mtDNA (Wiesenberger and Fox 1997).

The 5′-to-3′ exoribonuclease activity was ascribed to the Pet127 protein on the basis of genetic studies, mentioned above, investigating the changes in mitochondrial RNAs in the mutants. The first indications suggesting that the Pet127 protein may possess intrinsic ribonuclease activity came from in silico studies. Even though sequence homology is significant only for true orthologs of Pet127, application of remote homology detection algorithms revealed its similarity to the PD-(D/E)XK superfamily that includes nucleases such as Dxo1 or Rat1 (Steczkiewicz et al. 2012). Significantly, this similarity includes the presence of key catalytic residues in structurally conserved domains.

In order to study the exoribonuclease activity of Pet127p, we purified the protein and tested its activity toward different RNA substrates. As purification of the Pet127 protein of sufficient purity and solubility for enzymatic assays from more conventional model species proved to be challenging, we used the ortholog (TmPet127) from *Talaromyces marneffei*, a thermotolerant dimorphic ascomycetous fungus (Gauthier 2015).

In order to provide a comprehensive overview of the phenotypic effect of the *pet127* null mutation, we also constructed deletant and point catalytic mutant strains in the *Candida albicans PET127* gene ortholog (*CaPET127*), and analyzed their phenotypes on physiological and molecular level. In particular, we performed an RNA-seq analysis of the mitochondrial transcriptome in the mutants. Our previous studies (Kolondra et al. 2015; Łabędzka-Dmoch et al. 2021) confirmed the utility of *C. albicans* for mitochondrial transcriptomics, as unlike *S. cerevisiae*, this petite-negative yeast species has a stable mitochondrial genome that does not undergo frequent deletions or rearrangements, obfuscating the results in *S. cerevisiae*.

Our results indicate that even though the Pet127 protein is not essential for the functioning of the *C. albicans* respiratory system, its 5′-to-3′ exoribonuclease activity, confirmed in vitro using the *T. marneffei* ortholog, is involved in mitochondrial RNA metabolism, including degradation of intronic sequences.

## RESULTS

### Pet127 is an ancient eukaryotic protein lost in multiple independent lineages

**FIGURE 1.**
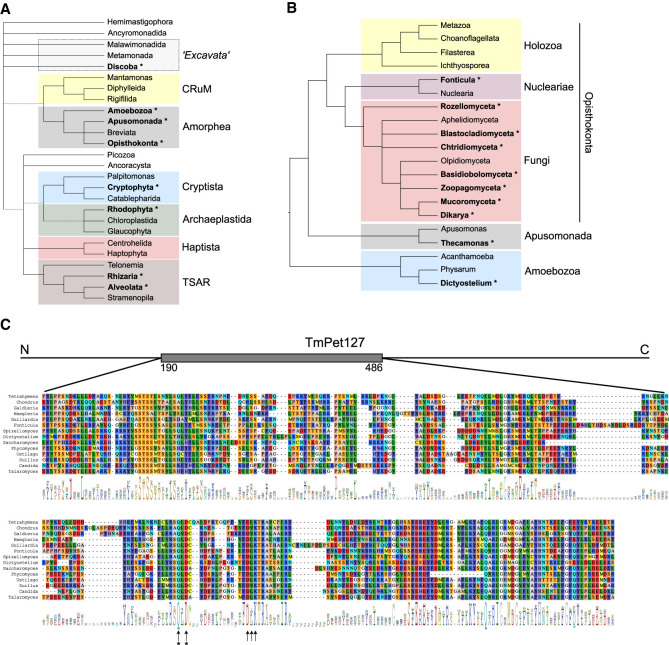
Pet127 is a conserved eukaryotic protein, lost in multiple independent lineages. (*A*) Presence of Pet127 in different eukaryotic supergroups. Names in bold with an asterisk indicate that at least one member of the group carries a Pet127 ortholog. Supergroups and putative tree topology are drawn following recent phylogenomic studies (Lax et al. 2018; Burki et al. 2020); dashed lines indicate uncertain monophyly of a group. Excavata are no longer considered a monophyletic supergroup. (*B*) Presence of Pet127 orthologs in Opisthokonta and related taxa. Tree topology follows the conclusions of phylogenomic analyses of Paps et al. (2013), fungal subkingdoms are according to Tedersoo et al. (2018). Ascomycete species, like *S. cerevisiae*, *S. pombe*, *C. albicans*, and *T. marneffei*, belong to Dikarya. (*C*) Alignment of Pet127p amino acid sequences from selected eukaryotic lineages (the full list of taxa and accession numbers can be found in Supplemental Table S1). Detailed alignment is shown for the most conserved core nuclease domain, its location in the sequence of *Talaromyces marneffei* Pet127 protein is marked on the schematic (gray box). Conservation is visualized as the sequence logo (Schneider and Stephens 1990) *below* the alignment, with the total height of the position indicating the degree of conservation measured as the information content on a scale of 0 to 4.3 bits. Arrows indicate the key amino acids in the conserved nuclease core (Kosinski et al. 2005; Steczkiewicz et al. 2012): E340, and the residues forming the variant of the PD-(D/E)XK motif (here QD-DLK): Q386, D388, D401, L402, K403. Residues mutated in the described experiments are marked with asterisks. Numbering follows that of the *T. marneffei* sequence.

Studies of the Pet127 protein concentrated on model yeasts belonging to the fungal phylum of Ascomycota. The absence of Pet127 orthologs in the genomes of multicellular animals and plants is apparent, and homologs from some unicellular Eukaryotes were identified previously (Wiesenberger et al. 2007). Using the wealth of recently obtained genomic sequences that provide new insights into the eukaryotic diversity and phylogeny (Katz and Grant 2015; Lax et al. 2018; Burki et al. 2020), we looked for Pet127 orthologs among the currently recognized major taxa (“supergroups”) (Burki et al. 2020). Orthology was determined using the reciprocal best hits criterion, with the *S. cerevisiae* Pet127 protein sequence (accession number NP_014660.1) as query. In particular, taxa used in recent phylogenomic studies of Eukaryotes (Katz and Grant 2015; Lax et al. 2018) were checked for the presence of Pet127 orthologs. The results, shown in Figure 1A, reveal that Pet127 is present in several divergent lineages, and absent in others, without a clear phylogenetic pattern. In the TSAR supergroup it can be found in Alveolata (in ciliates and dinoflagellates) and in Rhizaria, whereas in Archaeplastida only in the Rhodophytes (a list of identified taxa possessing a Pet127 ortholog is provided in Supplemental Table S1). Remarkably, it is also present in some members of the basal clade Discoba, as well as in *Guillardia theta*, belonging to Cryptophyta in the supergroup Cryptista. The number of complete sequenced genomes in most Protist lineages is not sufficient to exclude the possibility that some other clades may also harbor the Pet127 protein; however, given the large number of known plant genomes, the lack of the *PET127* gene in Chloroplastida appears certain.

The situation in Amorphea, the supergroup that contains Opisthokonta, Amoebozoa, and related clades, is of particular interest (Fig. 1B). Pet127 is entirely absent from Holozoa, the clade containing animals and related protist taxa. It is, however, present in the majority of Fungi, including Dikarya, the superphylum containing, among others, the extensively studied Ascomycota and Basidiomycota. It can also be found in *Fonticula*, a cellular slime mold related to Fungi, and in *Thecamonas,* a zooflagellate classified in Apusomonada (Paps et al. 2013). Among Amoebozoa, orthologs of Pet127 are encoded in the genomes of several dictyostelid slime molds (and one protostelid species), but neither in physarids, nor in unicellular amoebae with known genomic sequences.

The maximum likelihood phylogenetic tree of Pet127 amino acid sequences (Supplemental Fig. S1) is generally consistent with the proposed eukaryotic phylogeny, ruling out recent horizontal gene transfer as the explanation of the observed pattern of conservation. This suggests that Pet127 is an ancient eukaryotic protein that was probably present in the last common eukaryotic ancestor, and its absence in multiple lineages is most likely a result of several independent gene loss events.

Mitochondrial localization of the Pet127 protein in Fungi was demonstrated experimentally in yeast *S. cerevisiae* (Wiesenberger and Fox 1997), and can be predicted for other fungal orthologs. In silico prediction of protein localization in nonmodel organisms, particularly in taxa that were not extensively studied experimentally, remains a challenge. However, the application of an ML algorithm designed to predict the localization of proteins in mitochondria and mitochondrion-related organelles in nonmodel organisms (Kume et al. 2018) indicates that Pet127 orthologs from dinoflagellates, Rhodophyta, and Discoba should also localize to mitochondria, suggesting that subcellular localization is also a conserved feature of this protein.

Alignment of Pet127 amino acid sequences (Fig. 1C) shows that the core PD-(D/E)XK nuclease domain, including the key catalytic residues (Kosinski et al. 2005; Steczkiewicz et al. 2012) is conserved in diverse eukaryotic lineages, whereas the other regions show significant divergence, suggesting that the exoribonuclease activity of the protein is maintained by selective pressure. In the region responsible for the regulatory interaction with the Rpo41 mitochondrial RNA polymerase, corresponding to residues 48–215 in *S. cerevisiae* (Corbi and Amon 2021), only a short stretch (residues 178–208) shows conservation (Supplemental Fig. S2). This suggests that this interaction is either not universal, or it depends on those segments of the Rpo41 protein, located in the amino-terminal domain, that are also highly divergent (Ringel et al. 2011; Yang et al. 2015).

### Pet127 is not essential for the functioning of the *C. albicans* mitochondrial respiratory system

In *C. albicans,* the Pet127 ortholog (CaPet127) is encoded by the C1_11070W gene (orf19.2309), and is a protein of 705 amino acids sharing 24% sequence identity and 42% sequence similarity with the *S. cerevisiae* protein (accession number NP_014660.1). The homozygous deletion mutant *ΔCapet127/ΔCapet127* was constructed in the background of the BWP17 wild-type laboratory strain (Wilson et al. 1999) using two-step PCR based targeting (Walther and Wendland 2008). The heterozygous *ΔCapet127/CaPET127* strain obtained in the first step was also used in respiratory growth tests.

Additionally, the *Capet127_D375A_/ Capet127*_D375A_ point mutant strain was also constructed, encoding a form of the protein with the conserved D375 residue in the PD-(D/E)XK core nuclease motif (Fig. 1C) changed to alanine. This residue corresponds to D378 in the *S. cerevisiae* protein (ScPet127), and to D388 in *Talaromyces marneffei* (TmPet127).

**FIGURE 2.**
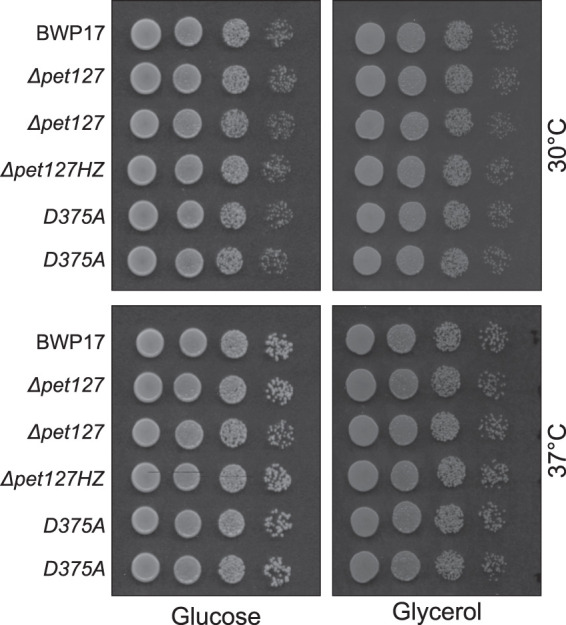
The homozygous *ΔCapet127* strain and functional mutants are viable and do not show respiratory deficiency. Growth on solid agar media containing glucose (YPD) or glycerol (YPG) as the carbon source. A series of 10× dilutions from overnight YPD starter cultures of wild-type (BWP17), heterozygous *CaPET127/ΔCapet127* (*Δpet127*HZ), homozygous *ΔCapet127* (*ΔCapet127*), and two independent point mutant *Capet127_D375A_/Capet127*_D375A_ (D375A) strains were spotted on plates and incubated for 48 h at 30°C or 37°C.

Respiratory competence of the deletant and point mutant strains was compared to that of the wild-type parental strain by assessing growth on solid media with nonfermentable (glycerol) and fermentable (glucose) carbon sources (Fig. 2). The test was performed at 30°C and 37°C. In all the tested conditions, the growth of homozygous and heterozygous deletants, as well as the point mutants, was indistinguishable from that of the wild-type strain. We can therefore conclude that the loss of Pet127 activity does not affect the respiratory growth capacity of *C. albicans*.

### Changes in steady-state mRNA levels and increased intron accumulation in Pet127 deficient mutants

**FIGURE 3.**
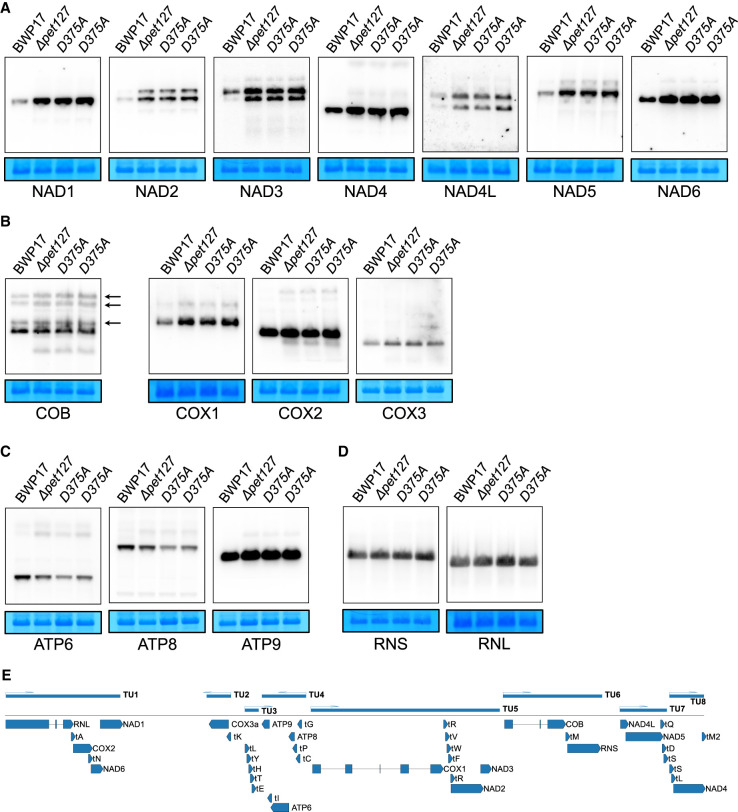
Changes in steady-state RNA levels in *ΔCapet127* and *Capet127*_*D375A*_ mutant strains. Northern blot analysis of mitochondrial mRNA and rRNA transcripts from wild-type (BWP17), homozygous *ΔCapet127* (*ΔCapet127*), mutant, and *Capet127*_*D375A*_ (D375A) catalytic mutants (two independent strains). (*A*) mRNAs encoding subunits of Complex I. (*B*) mRNAs encoding subunits of Complex III (COB) and Complex IV (COX). Arrows indicate the splicing intermediates of COB. (*C*) mRNAs encoding subunits of the ATP synthase (Complex V). (*D*) rRNAs of the small (RNS) and large (RNL) subunits of the mitoribosome. RNAs were prepared from purified mitochondria and separated by agarose/formaldehyde gel electrophoresis in denaturing conditions. Methylene blue staining of the small subunit mitochondrial rRNA in the blot is shown *below* each autoradiogram as a loading control. In panels *A*–*D*, blot series [NAD3, RNS]; [NAD4, COB]; [RNL, COX1]; [COX2, NAD5] were prepared by stripping and rehybridizing the same membrane, hence the same loading controls. (*E*) Schematic map of *C. albicans* mtDNA (without the second identical repeat region) showing the location of genes in primary transcription units (TU), according to Kolondra et al. (2015).

We performed a series of northern blot experiments using RNA isolated from mitochondria of the homozygous *ΔCapet127/ΔCapet127* deletant, as well as the *Capet127_D375A_/Capet127*_D375A_ point mutant strain in order to investigate the effect of Pet127 dysfunction on the major mitochondrial transcripts (Fig. 3). While the changes in steady-state levels and processing patterns of multiple transcripts are detectable, they are minor. None of the mRNAs and rRNAs show significantly decreased or largely aberrant expression, consistently with the observed lack of respiratory growth impairment. The size of the mature mRNAs and rRNAs in the mutant strains appears to be unchanged compared to the wild-type control. This contrasts with the observations made in *S. cerevisiae*, where several transcripts show decreased steady-state levels and increased length in *pet127Δ* strains (Wiesenberger and Fox 1997).

The three bicistronic transcripts encoding subunits of Complex I: NAD6–NAD1, NAD2–NAD3, and NAD4L–NAD5, as well as the monocistronic NAD4 mRNA all show increased steady-state levels (two- to fivefold according to blot quantification) in the mutant strains (Fig. 3A). In the case of NAD2, NAD3, and NAD4L probes, hybridization reveals that in mutant strains both bicistronic and monocistronic forms are present, even though the downstream ORFs overlap with the upstream ones, and are presumably translated only as a part of the bicistron (Kolondra et al. 2015). Similarly, the steady-state level of the mature mRNA encoding COX1 and, to a lesser extent, COX3 are slightly increased, whereas the mature COX2 mRNA level appears to be unchanged (Fig. 3B). The bicistronic ATP6–ATP8 transcript shows slightly decreased steady-state level of the mature mRNA (Fig. 3C), and both rRNAs are unaffected (Fig. 3D). In the case of the COB transcript, the level of mature mRNA is unaffected, but there is a visible increase in the signal from the processing intermediates (indicated by arrows on Fig. 3B).

In order to investigate the effect of Pet127 dysfunction on the mitochondrial transcriptome, we performed RNA-seq analysis on mitochondrial RNA from the homozygous *ΔCapet127/ΔCapet127* deletant, as well as the *Capet127_D375A_/Capet127*_D375A_ point mutant (two independent isolates that gave identical results), comparing them with the wild-type BWP17 strain. RNA sequencing libraries were prepared from isolated mitochondria and sequenced using the Ion Torrent Proton NGS System. We used the same RNA-seq workflow to describe the mitochondrial transcriptome of wild-type *C. albicans* (Kolondra et al. 2015), and to analyze the phenotype of mutants deficient in the mtEXO function (Łabędzka-Dmoch et al. 2021). The mtDNA sequence of *C. albicans* strain SC5314 (GenBank:AF285261.1), which is known to be identical to the mtDNA sequence of BWP17 (Kolondra et al. 2015), with one of the two identical 6.8 kb repeat regions removed, was used as the template for read mapping. 32–36 million reads were obtained from each sample, of which 92%–93% mapped to the reference sequence.

**FIGURE 4.**
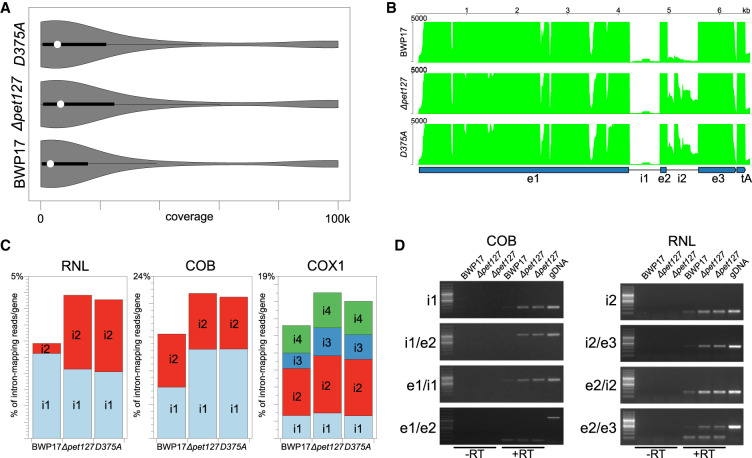
Increased intron accumulation in *ΔCapet127* and *Capet127*_*D375A*_ strains. (*A*) The distribution of sites in the mtDNA reference sequence with varying coverage in the wild-type (BWP17), homozygous *ΔCapet127* mutant, and *Capet127*_*D375A*_ catalytic mutant. Width of the plot corresponds to the frequency of sites covered by the number of RNA-seq reads shown on the *x*-axis. White circles and dark bars correspond to the median and the interquartile range (IQR), respectively. Coverage depth was calculated using the -depth option of SAMtools (Li et al. 2009) and visualized in R using the vioplot package. (*B*) Coverage by RNA-seq reads of the fragment of the *C. albicans* mtDNA reference sequence encompassing the RNL gene and the downstream tRNA-Ala [tA(UGC)mt] in the wild-type (BWP17), homozygous *ΔCapet127* deletant, and *Capet127*_*D375A*_ catalytic mutant. BWA files obtained using bamCompare (Ramírez et al. 2016) were visualized in pyGenomeTracks (Ramírez et al. 2018). The depth coverage axis was set at the maximum value of 5000 reads to better visualize low-coverage regions, truncating the highest values. (*C*) RNA-seq reads mapping to the intronic sequences in the RNL, COB, and COX1 genes, expressed as % of all reads mapping to the gene sequence (exons + introns). (*D*) Semiquantitative RT-PCR analysis of the introns and intron–exon junctions in the homozygous *ΔCapet127* mutant (two independent repeats) compared to the wild-type (BWP17) strain. Fragments internal to the intron, encompassing the intron–exon junctions, and across introns were amplified following reverse transcription (+RT) of DNase-treated mitochondrial RNA. Amplification of reactions with the reverse transcriptase omitted (−RT) were used to control for DNA contamination of the RNA samples, and genomic DNA (gDNA) was used as a positive control.

Visualizing the distribution of coverage depth (Fig. 4A) shows that the mutant strains do not show apparent differences from the wild-type, unlike the previously described mtEXO deficient mutants (Łabędzka-Dmoch et al. 2021), where the density distribution was significantly shifted toward higher coverage values. Counting reads mapping to the annotated primary transcripts (Kolondra et al. 2015) in the sense and antisense orientation shows that in all the analyzed strains the majority of reads (99.8%) map to the transcription units, with only 0.2% of reads corresponding to intergenic regions, regardless of the presence of the functional Pet127 protein. Of the reads mapping to the primary transcripts, only 0.01% are in the antisense (“mirror”) orientation, again with no differences between the wild-type and the mutants. This corresponds to the results obtained in wild-type *C. albicans* mitochondria in the previous studies, and is in stark contrast to the phenotype of mtEXO mutants, where both intergenic region and antisense RNAs show significant increase (Kolondra et al. 2015; Łabędzka-Dmoch et al. 2021). These results suggest that general mitochondrial RNA degradation and surveillance is not impaired in strains lacking the Pet127 activity.

Coverage graphs of regions corresponding to different mitochondrial genes (Supplemental Fig. S3) shows that apart from a moderate increase in coverage, consistent with the results of northern hybridizations, there are no significant qualitative differences between the mutants and the wild-type. Notably, there is no evidence of any unprocessed 5′ extensions that were observed in *S. cerevisiae pet127Δ* strains (Wiesenberger and Fox 1997). The most apparent phenotype is related to an increase in reads mapping to introns, evident in the case of the second intron (i2) of the RNL gene (Fig. 4B). Counting reads mapping to introns and exons of RNL, COB, and COX1 genes (Fig. 4C) shows that overall the proportion of intronic reads is increased about 1.5-fold in the deletant and mutant strains compared to wild-type. This is mostly due to the accumulation of the second intron of RNL (sevenfold increase), the first intron of COB (1.7-fold increase), and the third intron of COX1 (1.8-fold increase), the remaining introns are mostly unaffected.

In order to verify these observations, we performed semiquantitative RT-PCR of amplicons internal to the first intron of COB and the second intron of RNL, encompassing relevant exon–intron junctions, and across the introns from exonic primers (Fig. 4D). In the case of the first intron of COB, the increase is apparent for the internal intronic amplicon and both exon–intron junctions, indicating that the unspliced precursor and, potentially also the excised intron are accumulated in the Pet127 deletants. Similar results were observed for the second intron of RNL, with additional evidence from the amplification across the intron from primers located in the second and third exon, showing that the amount of spliced product is unchanged, while the unspliced precursor accumulates (in the case of COB amplification across the intron was not successful).

**FIGURE 5.**
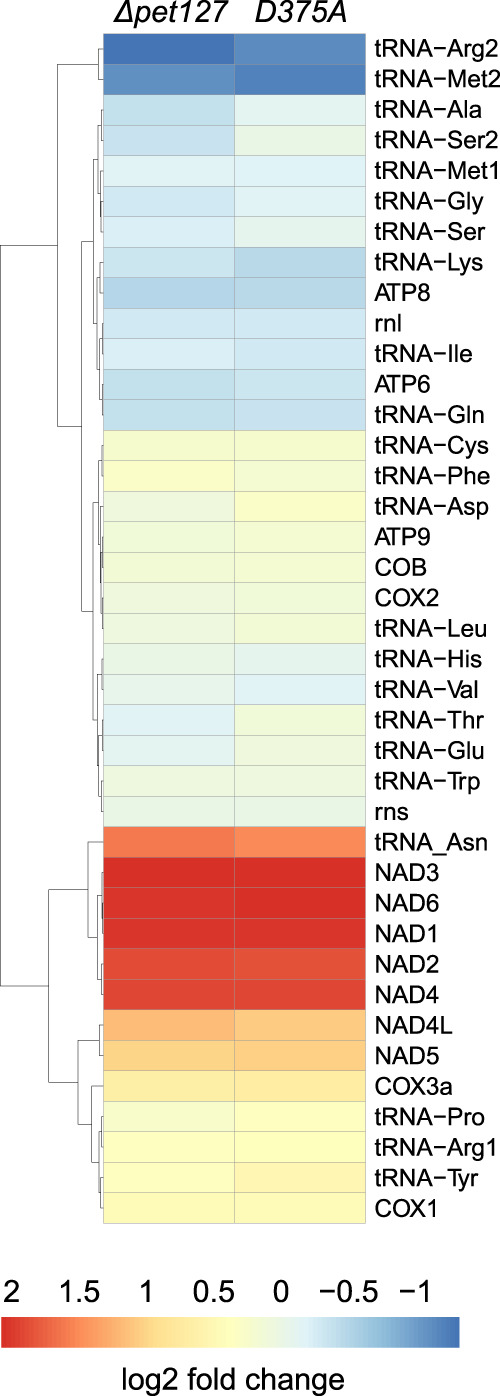
Changes in steady-state levels of mitochondrial transcripts in CaPet127 deficient strains estimated by RNA-seq. RNA-seq reads mapping to each gene were counted using featureCounts (Liao et al. 2014) and normalized using the TMM method in the edgeR package (Robinson et al. 2010). The heatmap presents log_2_ fold change of expression in the *ΔCapet127* deletant (*ΔCapet127*) and *Capet127*_*D375A*_ (D375A) mutant compared to the wild-type strain.

In order to estimate the quantitative changes in transcript levels in Pet127 deficient strains, we calculated the fold change of normalized read counts mapping to each gene compared to wild-type. The results (Fig. 5) are consistent with the observations made by quantitatively analyzing the northern blots, with a two- to fourfold increase in the expression of transcripts encoding the Complex I subunits (NAD genes) as the most prominent change.

Overall, the changes in the northern hybridization and RNA-seq patterns are moderate, quantitative rather than qualitative, and consistent with the lack of observable impairment of respiratory function in the deletant. In all the experiments, the D375A substitution resulted in a phenotype that was indistinguishable from that of the complete deletion, indicating that this mutation is sufficient to produce a nullomorphic allele with regard to the postulated exoribonuclease activity.

### Purified *T. marneffei* Pet127 protein is a progressive 5′-to-3′ exoribonuclease

Genetic and molecular studies described above in *C. albicans*, as well as those published earlier in *S. cerevisiae* (Wiesenberger et al. 2007; Fekete et al. 2008) and *S. pombe* (Wiesenberger et al. 2007) suggest that the Pet127 protein has a 5′-to-3′ exoribonuclease activity. The presence of conserved sequence and structure motifs indicates that it belongs to the PD-(D/E)XK superfamily (Steczkiewicz et al. 2012). In order to investigate the enzymatic activity of Pet127, we attempted to purify the catalytically active protein from different fungal species. The best results were obtained using the Pet127 ortholog from the filamentous fungus *Talaromyces marneffei*. It is a protein of 849 amino acids (accession number XP_002153343.1) sharing 24% sequence identity and 40,5% sequence similarity with the *C. albicans* protein, and 26% identity and 44% similarity with the *S. cerevisiae* ortholog. The conserved region encompasses the putative nuclease domain (Fig. 1C).

To determine Pet127 ribonucleolytic activity, we established the expression and purification protocol, then purified the full length wild-type protein as well as its two point mutants D388A and Q386A in the predicted motif II of the PD-(D/E)XK nuclease active site. These two residues correspond to D375 and Q373 in *C. albicans*, as well as to D236 and E234 of the extensively studied mammalian Dxo1 nuclease (Chang et al. 2012; Jiao et al. 2013), respectively. Its crystal structure (DOI: 10.2210/pdb4J7L/pdb) shows that both residues are responsible for coordination of the two magnesium ions in the active site (Jiao et al. 2013). Based on the sequence similarity of the catalytic domain within members of the PD-(D/E)XK nuclease superfamily, mutations of these residues in TmPet127 should also lead to inhibition of its ribonuclease activity. Northern blot and RNA-seq experiments, described above, demonstrated that the D375A mutation in *C. albicans* (corresponding to D388A in *T. marneffei*) has a phenotype indistinguishable from that of the complete deletion.

**FIGURE 6.**
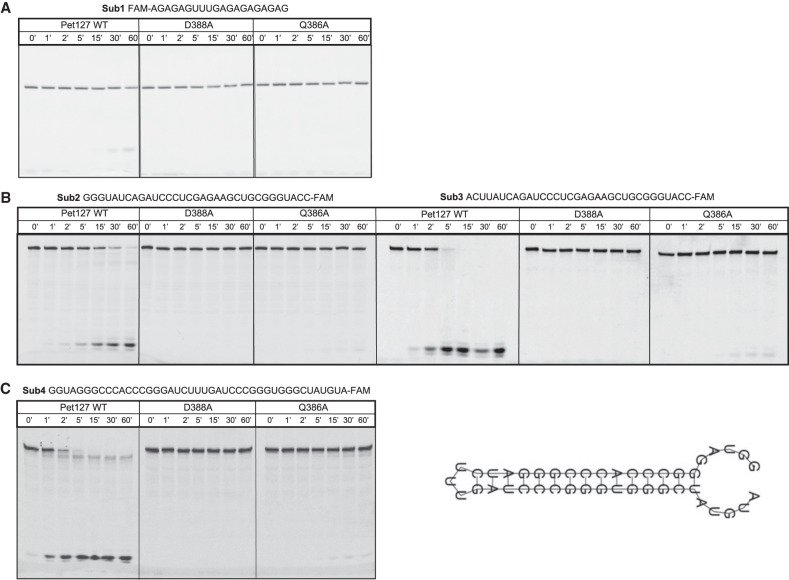
Purified *T. marneffei* Pet127 is a processive 5′-to-3′ exoribonuclease. RNA degradation activity of *Tm*-Pet127 wt and point mutants against. (*A*) 5′ fluorescein (FAM) labeled single-stranded RNA substrate (ssRNA), (*B*) 5′ G-rich ssRNA compared to a random sequence, (*C*) hairpin RNA structure within the mRNA ATP6/8 transcript from *C. albicans*. Degradation products were analyzed by 20% denaturing TBE-urea PAGE.

To investigate the exoribonuclease activity of Pet127, we conducted activity assays against substrates (sequences of the substrates are listed in Supplemental Table S2 and Fig. 6) labeled with fluorescein either on 5′ end (Fig. 6A) or 3′ end (Fig. 6B,C). While 3′-labeled substrates were efficiently degraded by Pet127, the 5′-labeled substrate remained undigested. This indicates that the Pet127 protein has an intrinsic 5′-to-3′ exoribonuclease activity, consistent with the hypothesis formulated based on genetic studies and sequence analysis. In this experiment, Pet127 is active toward different substrates. The activity toward a substrate which comprised a 5′-terminal tract of Gs is slightly diminished in the comparison to an RNA of the same length without a 5′-terminal G (Fig. 6B). This feature was also found within Dxo1/Rai1 hydrolases, members of the same PD-(D/E)XK superfamily (Doamekpor et al. 2020). Finally, we tested Pet127 activity toward a hairpin-structure-forming RNA, derived from the *C. albicans* ATP6/8 mRNA transcript (Fig. 6C; Kolondra et al. 2015). Pet127 is able to degrade this type of structured RNA, although with lower efficiency than for unstructured RNA. The single-stranded end of this substrate is degraded quickly, and the remaining ds-RNA hairpin appears to be partially resistant to degradation. This suggests that the Pet127 5′-to-3′ exoribonuclease activity is not sequence or structure-dependent, although it is partially inhibited by 5′-terminal G tracts and strong secondary structure regions. Degradation of all the substrates is complete, leaving only a 3′-FAM labeled nucleotide as the final product (Supplemental Fig. S4), even at the earliest time points, suggesting that the exoribonuclease activity of Pet127 is processive.

In all the assays, the activity of D388A and Q386A TmPet127 variants was severely reduced (Fig. 6). The D388A substitution removed any detectable activity of the protein, consistent with the experiments performed in the D375A *C. albicans* mutant, which was indistinguishable from the deletant in RNA experiments. The substitution at Q386 also severely diminished the exoribonuclease activity, although traces of the degradation product, corresponding to 6%–7% of the wild-type activity, can be detected at later time points.

## DISCUSSION

The PD-(D/E)XK protein superfamily is very large and extremely diverse, containing members with a number of different roles and activities, including both active nucleases and their inactive homologs (Steczkiewicz et al. 2012). It includes prokaryotic restriction endonucleases, proteins involved in DNA repair and recombination, tRNA intron splicing factors, and exoribonucleases involved in mRNA decapping and degradation in Eukaryotes (Poole and Stevens 1995; Stevens and Poole 1995; Belfort and Weiner 1997; Ban and Yang 1998; Aravind et al. 2000; Hickman et al. 2000; Kosinski et al. 2005; Orlowski and Bujnicki 2008; Xiang et al. 2009; Steczkiewicz et al. 2012; Jiao et al. 2013; Doamekpor et al. 2020). Exoribonucleases containing the PD-(D/E)XK fold include both processive (Xiang et al. 2009; Miki and Großhans 2013) and distributive (Jiao et al. 2013; Doamekpor et al. 2020) enzymes. Identification of the PD-(D/E)XK motif and the associated conserved fold in the Pet127 protein (Steczkiewicz et al. 2012) was therefore not sufficient to infer its enzymatic activity.

A 5′-to-3′ exoribonuclease activity was suggested for Pet127 based on a series of genetic studies conducted in model yeasts *S. cerevisiae,* where the *pet127Δ* strains accumulate several transcripts with longer, unprocessed 5′ termini (Wiesenberger and Fox 1997; Fekete et al. 2008). In the best studied case of the COB transcript, the 5′ end of the mature mRNA is determined by the site of Cbp1 protein binding, which protects it from the 5′-to-3′ exoribonuclease activity that is dependent on the product of the *PET127* gene (Fekete et al. 2008). Degradation dependent on the Pet127 protein activity was also shown to play a role in producing different steady-state levels of mature RNAs derived from a single primary transcript encompassing COB and tRNA-E (Krause 2004). None of these studies, however, provided decisive proof of intrinsic ribonuclease activity in the Pet127 protein.

In this work we also purified the *T. marneffei* Pet127 protein and conducted ribonuclease activity assays. In order to verify whether the PD-(D/E)XK domain is responsible for Pet127 activity, we also purified mutein forms with substitutions in two amino acid residues that are crucial for the catalytic activity by coordinating magnesium cations in the active site of the enzyme, as evidenced from the study of mammalian Dxo1 exoribonuclease (Jiao et al. 2013). These two residues are strictly conserved in all the Pet127 orthologs we identified in the available sequence databases.

Our results (Fig. 6) indicate that the purified TmPet127 protein does possess a readily detectable exoribonuclease activity in vitro, and that mutations in the residues predicted to be crucial for the PD-(D/E)XK domain active site completely, or nearly completely abolish this activity. We can thus conclude that Pet127 is a bona fide ribonuclease of the PD-(D/E)XK superfamily. Assays using 5′ or 3′ labeled substrates indicate that RNA degradation by the TmPet127 protein occurs from the 5′ terminus, and the lack of any detectable intermediates even at the earliest time points suggests a processive exoribonucleolytic activity. Pet127 in vitro is capable of complete degradation of the substrate, leaving only the single labeled nucleotide as product, similar to the Dxo1 exoribonuclease (Chang et al. 2012; Jiao et al. 2013). Results of assays with different substrates indicate that the TmPet127 activity is not sequence-specific, although its activity toward a substrate containing multiple Gs at the 5′ terminus is visibly reduced. It is even capable of degrading, albeit with a reduced efficiency, a substrate containing a region of strong hairpin secondary structure. Unlike another well studied mitochondrial exoribonuclease, the Dss1 protein, where its intrinsic 3′-to-5′ exoribonuclease activity is low, and it requires the action of the Suv3 RNA helicase to efficiently degrade even short unstructured substrates (Malecki et al. 2007; Razew et al. 2018), the Pet127 5′-to-3′ exoribonuclease is highly active in vitro without any accessory proteins.

Most data on the physiological role of the Pet127 exoribonuclease come from the studies in model yeast *S. cerevisiae*. The most apparent phenotype of mutants lacking Pet127p in this species is associated with abnormal processing of 5′ termini of multiple mRNA and rRNA transcripts, associated with visible decrease in the mRNA levels of COX2, ATP6, and ATP9 (Wiesenberger and Fox 1997). In contrast, the loss of the Pet127 protein in *Schizosaccharomyces pombe* does not cause apparent RNA maturation defects, but results in a 1.5- to 4-fold increase in the steady-state level of mature RNAs, most apparent for COX2 and COX3, suggesting that in this species the Pet127 exoribonuclease is involved in general RNA degradation rather than processing (Wiesenberger et al. 2007). In order to further investigate the biological function of Pet127, we performed genetic analysis in *Candida albicans*, which is an attractive mitochondrial model due to the stability of its mtDNA (no formation of cytoplasmic petites), and well-studied organellar transcriptome (Kolondra et al. 2015; Łabędzka-Dmoch et al. 2021).

The results of molecular phenotype investigations using northern blot hybridizations (Fig. 3) and RNA-seq transcriptome analysis (Figs. 4, 5) indicate that in *C. albicans*, unlike in *S. cerevisiae*, there are no apparent defects in RNA maturation in deletants and mutants lacking Pet127. The number of RNA-seq reads covering 5′ regions of transcripts does increase, but there is no detectable extension of the mature RNAs upstream beyond the 5′ terminus of transcripts observed in the wild-type strain. As in *S. pombe*, the steady-state levels of several mRNAs increase in the mutants by a similar amount (two- to fourfold), but this increase does not involve all the transcripts (Fig. 5). The effect is most apparent for the mRNAs of genes encoding the subunits of Complex I—the bicistronic NAD6–NAD1, NAD2–NAD3, and NAD4L–NAD5 mRNAs, as well as the monocistronic NAD4. The expression of tRNA-Asn, located in the first transcription unit just upstream of NAD6 is also increased. Several transcripts, mostly tRNAs and ATP6–ATP8 bicistronic mRNA show a slight (by about 25%) decrease in expression. Curiously, the transcripts that show visibly increased levels in the mutant strains are located close to the 3′ end of their respective polycistronic primary transcription units (see Fig. 3E). tRNA-Asn, NAD6, and NAD1 form the 3′ terminal half of the first transcription unit (TU1) that starts with RNL, NAD2 and NAD3 are downstream from COX1 in TU5, and NAD4 is transcribed as a part of TU8 downstream from tRNA-Leu and tRNA-Met-2. This suggests that degradation by the Pet127 5′-to-3′ exoribonuclease probably occurs after the primary transcripts are processed into separate RNAs by the tRNA punctuation mechanism (Kolondra et al. 2015).

Another phenotype of Pet127 deficient mutants in *C. albicans* is related to the accumulation of intronic sequences, evidenced by RNA-seq and confirmed by RT-PCR (Fig. 4). The effect is most pronounced for the second intron in RNL, with about a sevenfold increase in the fraction of reads mapping to the intron among all reads mapping to the gene. A more moderate increase is also observed for the first intron of COB (1.7-fold), and the third intron of COX1 (1.8-fold). Semiquantitative RT-PCR analysis performed for the second intron of RNL and the first intron of COB confirms the RNA-seq results and additionally shows that the increase involves not only the excised intronic RNA, but also the unspliced precursor. These changes do not, however, significantly affect the levels of respective mature spliced RNAs. This indicates that in *C. albicans* the Pet127 exoribonuclease is involved in the degradation of introns and splicing intermediates, a role that was previously ascribed mostly to the main mitochondrial mtEXO ribonuclease (Golik et al. 1995; Dziembowski et al. 2003; Łabędzka-Dmoch et al. 2021). Based on the available data we cannot, however, explain why certain introns accumulate in mutants lacking Pet127, while others in the same gene do not.

In principle, observed changes in RNA steady-state levels could be attributed to changes in transcription, as well as to impaired degradation. Mitochondrial transcripts are, however, polycistronic, and changes in transcription would affect multiple RNAs transcribed from the same promoter, as well as multiple introns of the same gene. This is clearly not the case here, as, for example, the level of the COX2 transcript, transcribed from the same promoter as the visibly increased NAD1 and NAD6 RNAs, remains unchanged (see Fig. 3E). Similarly, only the first of the two COB introns shows accumulation in the mutant strains. It is thus reasonable to ascribe the observed phenotype to changes in RNA degradation rather than transcription, using steady-state transcript levels as proxy.

These results naturally raise questions about the functional interplay between the known mitochondrial exoribonucleases. In the nucleus and cytoplasm, both the 5′ and 3′ exoribonucleolytic pathways participate in RNA turnover and surveillance (Houseley and Tollervey 2009). In mitochondria, the 3′-to-5′ exoribonucleolytic degradation pathway provided by the mtEXO complex is universally conserved, although the exoribonuclease subunit itself can be one of two different types: a phosphorolytic PNPase in plants and animals, and a hydrolytic Dss1 ortholog in other lineages (Dziembowski et al. 2003; Holec et al. 2006; Hoffmann et al. 2008; Mattiacio and Read 2008; Borowski et al. 2013). The mtEXO activity is essential for the functioning of the mitochondrial genetic system (Dziembowski et al. 2003; Rogowska et al. 2006; Szczesny et al. 2010; Dhir et al. 2018; Pietras et al. 2018a,b; Łabędzka-Dmoch et al. 2021). In contrast, the 5′-to-3′ activity of Pet127 is neither essential, nor universal. Nullomorphic *pet127* mutants of *S. cerevisiae* and *S. pombe* show no impairment of respiratory function at normal growth conditions, and only a moderate phenotype at elevated temperatures (Wiesenberger and Fox 1997; Wiesenberger et al. 2007). Similarly, in *C. albicans* the loss of Pet127 does not produce any measurable growth impairment on respiratory media. In *S. cerevisiae*, overexpression of Pet127 partially rescues the phenotype of reduced mtEXO activity caused by the deletion of the gene encoding the Suv3 helicase component (Wegierski et al. 1998), suggesting that the two RNA degradation pathways are to a certain extent redundant.

A recent study (Corbi and Amon 2021) demonstrated that in *S. cerevisiae* Pet127 acts as a negative regulator of the mitochondrial RNA polymerase (Rpo41) in a manner that is independent of its RNase domain, raising a possibility that the principal biological role of this protein could be regulatory rather than enzymatic. In our study, however, a single amino acid substitution (D375A in CaPet127, equivalent to D388A in TmPet127) that abolished the ribonucleolytic activity produced a phenotype indistinguishable from that of a complete deletion in RNA based assays, suggesting that it is the exoribonuclease activity which is responsible for the phenotype in *C. albicans*. With respect to changes in the steady-state levels of mitochondrial transcripts this phenotype also resembles that of the *pet127Δ* deletant in *S. pombe*, suggesting that the involvement of Pet127 in general RNA degradation is the conserved function of this protein, at least in diverse Ascomycete lineages. The PD-(D/E)XK domain with its critical residues is also the part of the Pet127 protein sequence which is conserved among orthologs from diverse eukaryotic lineages. The primary conserved role of Pet127 thus appears to be related to its exoribonuclease activity rather than the non-enzymatic regulatory function.

The presence of Pet127 orthologs in diverse lineages, including early branching groups like Discoba (Fig. 1), suggests that it is an ancient eukaryotic protein. Pet127 forms a distinct subfamily among PD-(D/E)XK nucleases, with significant similarity on the sequence level limited to true orthologs. Its relationship to other members of the superfamily, like Dxo1 (Chang et al. 2012; Jiao et al. 2013; Doamekpor et al. 2020) or Rat1 (Xiang et al. 2009) becomes apparent only upon application of remote homology detection methods (Steczkiewicz et al. 2012). Mitochondrial localization of Pet127 is experimentally supported in Fungi, and predicted in organisms as diverse as dinoflagellates, Rhodophyta, and Discoba. There is no convincing evidence for the presence of any Pet127 homologs in Bacteria or Archaea (a few positive hits in Bacteria show very high similarity to fungal sequences and thus probably are a contamination artifact or a result of recent horizontal gene transfer). In Bacteria, the only common 5′-to-3′ exoribonuclease is RNase J, also found in chloroplasts of plants (Halpert et al. 2019), which is unrelated to Pet127 and does not belong to the PD-(D/E)XK superfamily. Pet127 appears thus to be an early eukaryotic evolutionary invention, and its absence of Pet127 in multiple lineages, most apparent in Holozoa (including animals) and Chloroplastida (including higher plants) is most parsimoniously explained by independent gene loss events. Genetic studies in three different yeast species show that Pet127 is not essential for the functioning of mitochondria, and its loss does not produce an obvious phenotype under laboratory conditions. It is thus not surprising that it was easily lost in the evolution of several distinct evolutionary lineages. What is more perplexing is the actual biological function of Pet127 underlying its conservation in all the diverse eukaryotic groups that retained its orthologs. The organisms that either lost or retained Pet127 represent a multitude of diverse mitochondrial genome organizations (Gray et al. 2004; Wideman et al. 2020; Zardoya 2020) and include both unicellular and multicellular organisms.

The results demonstrating the role of Pet127 in the degradation of intron-containing transcripts prompted us to investigate whether mitochondrial introns, particularly those belonging to Group I, could be linked to its biological function. Animal mitochondria are mostly intronless, with the exceptions mostly attributed to horizontal gene transfer events, and plant mitochondria are rich in Group II introns, but not in Group I (Haugen et al. 2005; Nielsen and Johansen 2009; Huchon et al. 2015; Mukhopadhyay and Hausner 2021). There are, however, no Group I introns in the mitochondrial genomes of Rhodophyta, and ciliate mtDNAs generally lack introns, yet both groups contain Pet127 orthologs. On the other hand, multiple Group I introns are present in the mitochondrial genomes of Choanoflagellata (Burger et al. 2003), which lack Pet127 like all other known Holozoa. The key to the primary biological function of Pet127 and its conservation in some, but not all, eukaryotic lineages must therefore lie elsewhere, and remains to be elucidated. Investigating the primary function of this mysterious exoribonuclease will entail moving beyond experiments in model organisms under laboratory conditions, and require combining functional and evolutionary approaches. We believe that it shall reveal interesting insights into the origin and functioning of the eukaryotic cell architecture.

## MATERIALS AND METHODS

### Strains and media

*Candida albicans* strain BWP17(*arg4::hisG/arg4::hisG, his1::hisG/his1::hisG, ura3*::*imm434*/*ura3*::*imm434, iro1*::*imm434* /*iro1*::*imm434*) (Wilson et al. 1999) was used as the wild-type control and the starting point for construction of mutants. The *ΔCapet127/ΔCapet127* strain (*arg4*/*arg4*, *his1*::*hisG*/*his1*::*hisG, ura3*::*imm434*/*ura3*::*imm434*, *iro1*::*imm434*/*iro1*::*imm434*, *pet127*::*HIS1*/*pet127*::*SAT*) was constructed using PCR based targeting (Walther and Wendland 2008). The first allele was disrupted by integration of the *CaSAT1* gene with ∼40 nt flanks homologous to the upstream and downstream sequence of *CaPET127,* amplified by PCR on the template of pFASAT1 (Walther and Wendland 2008). In the second round, a deletion cassette was constructed by yeast recombinational cloning (Colot et al. 2006). The *CaHIS1* gene from the plasmid pFAHIS1 (Walther and Wendland 2008), together with ∼1 kb flanks upstream and downstream from *CaPET127*, all obtained by PCR, were used for in vivo recombination into the pRS426 vector in the *S. cerevisiae* (*MATα, ade2*-*1, leu2*-*3, 112 ura3*-,*1 trp1*-*1, his3*-*11, 15 can1*-*100* [rho^+^ intronless]) strain (Saint-Georges et al. 2002). Deletion cassettes were amplified on the template of recombined plasmids by PCR and introduced into *C. albicans* heterozygous strains by electroporation (Reuss et al. 2004). Genotypes of obtained knockouts were confirmed by PCR.

The Capet127_D375A_/ Capet127_D375A_ strain (arg4/arg4, his1::hisG/his1::hisG, ura3::imm434 /ura3::imm434, iro1::imm434 /iro1::imm434, PET127::pet127_D375A_/PET127:: pet127D375A, eno1::CaCAS9-SAT/ENO1) was constructed using the *C. albicans* CRISPR/Cas9 Solo system as described previously (Vyas et al. 2015). The guide sequence 5′-CTTTTTATTGAGATCACAGT-3′ started 23 nt upstream of the GAT encoding Asp (D). Aspartic acid to alanine substitution was performed by introduction of a repair template bearing GAT (Asp)-GCA (Ala) mutation introducing SphI restriction site used for diagnostic RLFP and silent PAM mutation (TTG to TTA encoding leucine). To obtain the repair template, first the pET28aSumo vector with cloned CaPET127 was subjected to site directed mutagenesis with the primers KL182 and KL183. Later the vector pET28aPET127_D375A_Sumo was used as a PCR template with the primers KL184 and KL185 to amplify the donor DNA for homologous recombination. Sequences of all primers used in the construction and verification of strains are listed in Supplemental Table S2.

For isolation of mitochondria, strains were grown in liquid YPGal medium (1% yeast extract, 2% peptone, and 2% galactose) containing 80 g/L uridine at 37°C until logarithmic growth phase. Respiratory growth was tested on agar plates with either YPD (1% yeast extract, 2% peptone, and 2% glucose) or YPG (1% yeast extract, 2% peptone, and 2% glycerol).

### Isolation of mitochondria and RNA extraction

RNA was obtained from purified mitochondria isolated from log-phase liquid cultures of *C. albicans* grown in YPGal as described previously (Kolondra et al. 2015). Isolation of mitochondrial RNA was performed by hot phenol procedure (Schmitt et al. 1990) or by Fenozol (A&A Biotechnology) extraction. RNA samples were treated with 0.6U of DNase I (Roche) per μg of RNA according to the manufacturer's protocol. DNase-treated RNA was phenol-extracted, precipitated, and resuspended in water as described previously (Kolondra et al. 2015).

### Northern blot analysis

Northern hybridization was performed essentially as described previously (Malecki et al. 2008; Kolondra et al. 2015). Briefly, 2.5 µg of mitochondrial RNA was separated on a 1% denaturing formaldehyde gel, transferred onto Nytran N nylon membrane (GE Healthcare) and hybridized with the appropriate probe. The probes and ^32^P labeling protocols used to detect each transcript were described previously (Kolondra et al. 2015). The blots were scanned on the Typhoon FLA 9000 (GE) biomolecular imager, and quantitative analysis was performed in Fiji (Schindelin et al. 2012).

### Transcriptome sequencing, mapping, and analysis

The RNA-seq experiments were performed essentially as described previously (Łabędzka-Dmoch et al. 2021). Briefly, mitochondrial RNA-seq libraries were obtained using 400–500 ng RNA prepared from purified mitochondria, using the Ion Total RNA-Seq Kit v2 (Thermo Fisher Scientific) according to the manufacturer's protocol. The libraries were sequenced on the Ion Torrent Proton NGS System according to the manufacturer's instructions. Raw sequencing data were processed using the Torrent Suite Software (Life Technologies). Barcode removal and quality trimming were performed in Torrent Suite using default parameters (30% QC threshold, reads <25 nt rejected). The processed reads were exported as FASTQ files.

The complete mtDNA sequence of *C. albicans* strain SC5314 (GenBank:AF285261.1), with one of two identical copies of the inverted repeat region removed, and additional annotations (Kolondra et al. 2015) were used as a reference. Reads were mapped to the reference sequence using BWA-mem (Li and Durbin 2009), and SAMtools (Li et al. 2009) was used to manipulate the resulting alignments and to calculate coverage depth for each position in the reference sequence. Coverage graphs were obtained by visualizing BWA files obtained using bamCompare from the deepTools2 package (Ramírez et al. 2016) in pyGenomeTracks (Ramírez et al. 2018). The vioplot package in R was used to visualize coverage depth distributions. Reads mapping to chosen features were quantified using featureCounts (Liao et al. 2014) from the Rsubread package (Liao et al. 2019). Counts were normalized using the TMM method in the edgeR package (Robinson et al. 2010) for expression comparison between wild-type and mutants.

### RT-PCR analysis

An amount of 5 µg of DNase-treated RNA was reverse transcribed using Maxima Reverse Transcriptase (Thermo Scientific) with random hexamers. An amount of 2 µL of 10-fold diluted RT product was amplified in 20 µL with 0.5 U of Phusion polymerase (Thermo Scientific) and 10 pmol specific primers and analyzed by agarose gel electrophoresis. The number of amplification cycles was experimentally adjusted to stop the reaction before reaching the plateau phase. Sequences of all primers used in RT-PCR are listed in the Supplemental Table S2.

### In silico analysis

Searching for Pet127 orthologs was performed using a BLAST algorithm (Altschul et al. 1990) against amino acid (blastp) and translated nucleotide (tblastn) sequence databases at NCBI (NCBI Resource Coordinators 2018), with the *S. cerevisiae* Pet127 protein sequence (Acc# NP_014660.1) as query. Identified sequences were then used to search (blastp) the *S. cerevsiae* protein sequences, and only those that gave ScPet127 as the top hit were retained (reciprocal best hits criterion for orthology). Additionally, sequences that had long stretches of 100% sequence identity with Pet127 proteins from taxa classified in different kingdoms were ruled out as contamination—this eliminated two hits in plant (tree) sequences with stretches of 100% identity with Pet127 proteins from xylophagous fungi, and one avian sequence with complete 100% identity with *S. cerevisiae*. Global alignment amino acid sequence identity and similarity was calculated using the Needleman–Wunsch algorithm in STRETCHER from the EMBOSS suite (Rice et al. 2000). NommPred (Kume et al. 2018) was used to predict mitochondrial localization.

Amino acid sequences were aligned using muscle (Edgar 2004) and analyzed in SeaView v. 5 (Gouy et al. 2021). For phylogenetic analysis, the alignments were trimmed to remove noninformative regions in BMGE (Criscuolo and Gribaldo 2010) using the BLOSUM30 similarity matrix. Phylogenetic tree was inferred using IQ-TREE (Nguyen et al. 2015) under the LG + F + R3 model. Branch support was assessed using the ultrafast bootstrap approximation (Hoang et al. 2018) with 1000 repeats and the nearest neighbor interchange (NNI) search.

### Protein expression and purification

Codon optimized gene for *Talaromyces marneffei* Pet127 (TmPet127) was synthesized (BioBasic Inc.) and cloned into the expression vector pET28a-SUMO containing a His_6_-SUMO (small ubiquitin-related modifier) tag before the gene sequence. Mutations in the plasmid were introduced using QuikChange Site-Directed Mutagenesis kit (Agilent). Full length wild-type and point mutants were expressed in *E. coli* BL21 (DE3) Star strain. The bacteria cultures were grown in Luria broth (LB) medium at 37°C, induced with 0.4 mM isopropyl β-d-1-thiogalactopyranoside at OD_600_ = 0.6 and grown overnight at 18°C. The cells were harvested by centrifugation and pellets were washed with phosphate-buffered saline (PBS) prior to purification. All proteins (wild-type and mutants) were purified using the same protocol. The pellet from bacteria that expressed the desired protein was lysed by sonication in buffer containing 50 mM Tris (pH 7.5), 500 mM NaCl, 10 mM imidazole, 5% (v/v) glycerol, and 5 mM 2-mercaptoethanol (buffer A).

The lysate was clarified by centrifugation at 186,000*g* at 4°C for 40 min and the supernatant was loaded onto a His-Trap HP column (GE Healthcare) equilibrated buffer A. Protein was eluted using a step gradient of imidazole (10–300 mM in buffer A). The fractions were analyzed by sodium dodecyl sulfate-polyacrylamide gel electrophoresis (SDS-PAGE). Fractions that contained Tm-Pet127 (buffer A containing 300 mM imidazole) were dialyzed in buffer B containing 20 mM Tris (pH 7.0), 100 mM NaCl, 5% (v/v) glycerol, and 1 mM dithiothreitol (DTT). SUMO protease has been added to the protein solution in order to remove the His_6_-SUMO tag. To remove nucleic acid contamination, the protein was then loaded onto a HiTrap Heparin HP column (GE Healthcare) and eluted using a step gradient of NaCl (100mM–1 M in buffer B). After confirming by SDS-PAGE the presence of Tm-Pet127 in fractions eluted with 1 M NaCl in buffer B, the fractions were pooled together and concentrated to 0.5 mL. Concentrated fractions were loaded onto Superose 6 Increase size exclusion column, equilibrated previously with a buffer C that contained 20 mM Tris (pH 7.0), 1 M NaCl, 5% (v/v) glycerol, 1 mM MgCl_2_, and 1 mM DTT. Fractions that contained Tm-Pet127 protein were concentrated using a 50k MWCO Amicon Ultra Centrifugal Filter Device (Millipore).

### Exoribonuclease activity assays

6-carboxyfluorescein (FAM) labeled RNA substrates were synthesized by Future Synthesis. The sequences of the oligonucleotides are presented in the Supplemental Table S2. Exoribonuclease assays were performed with 90 nM protein concentration and 30 nM RNA in a reaction buffer that contained 20 mM Tris (pH 8.5), 50 mM KCl, 2 mM MgCl_2_, and 0.5 mM DTT. The reaction was stopped at selected time-points by the addition of an equal volume of 95% formamide with Orange G dye and boiling the sample for 5 min at 95°C. Reaction products were resolved by 20% TBE-Urea PAGE and visualized with Typhoon Trio Imager (GE Healthcare).

## DATA DEPOSITION

RNA-seq reads were deposited at NCBI Sequence Read Archive (SRA) repository under accession number SRP346086 (https://www.ncbi.nlm.nih.gov/sra/?term=SRP346086).

## SUPPLEMENTAL MATERIAL

Supplemental material is available for this article.

## Supplementary Material

Supplemental Material

## Figures and Tables

**Karolina Łabędzka-Dmoch:**
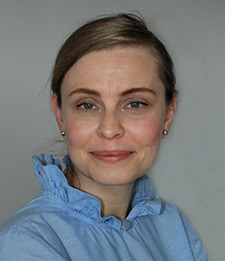


**Michal Rażew:**
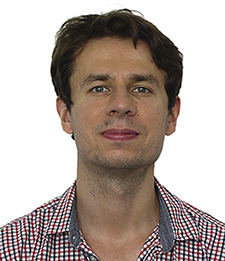


## MEET THE FIRST AUTHORS

***Meet the First Author(s)* is a new editorial feature within *RNA*, in which the first author(s) of research-based papers in each issue have the opportunity to introduce themselves and their work to readers of *RNA* and the RNA research community. Karolina Łabędzka-Dmoch and Michal Rażew are co-first authors of this paper, “The Pet127 protein is a mitochondrial 5′-to-3′ exoribonuclease from the PD-(D/E)XK superfamily involved in RNA maturation and intron degradation in yeasts.” Karolina is an assistant professor at the Institute of Genetics and Biotechnology, Faculty of Biology, University of Warsaw. She works in the laboratory of Professor Paweł Golik on nucleo-mitochondrial interactions and gene expression in the mitochondria of fungi. Michał started working on this project while he was a PhD student in Marcin Nowotny's laboratory at the International Institute of Molecular and Cell Biology in Warsaw, Poland. Currently he is a postdoctoral fellow at EMBL Grenoble in Wojciech Galej's group where he studies macromolecular complexes involved in transcription termination.**

**What are the major results described in your paper and how do they impact this branch of the field?**

Pet127 is a 5′ to 3′ mitochondrial processive exoribonuclease, belonging to the PD-(D/E)XK superfamily. We were able to demonstrate for the first time the activity of the fungal Pet127 in vitro. We show that Pet127 is not essential in *Candida albicans* pathogenic yeast and that mutation at its active site causes the same phenotype as the deletion of the gene. We prove that the physiological role of the Pet127 protein depends on its exoribonuclease activity.

**What led you to study RNA or this aspect of RNA science?**

**KLD:** Since joining Professor Pawe Golik's lab for my postdoc, I have focused on mitochondria and nucleo-mitochondrial interactions in fungi. At one point, I was involved in a project concerning PPR proteins, which are responsible for various aspects of mtRNA metabolism. This led to the degradation of the RNA in mitochondria of *Candida albicans* and *Saccharomyces cerevisiae*, but also to the comparative analysis of these model organisms.

**MR:** My interest in RNA biology started from my PhD project where I studied the structure and function of the yeast mitochondrial RNA degradosome complex mtEXO. This 3′ to 5′ decay machinery is the key regulator of mitochondrial gene expression in yeast, so I was really excited to join forces with the team working on the Pet127 exoribonuclease, which degrades RNA from the opposite site. As a result, we now have a deeper insight into RNA decay in yeast mitochondria.

**During the course of these experiments, were there any surprising results or particular difficulties that altered your thinking and subsequent focus?**

Yeast Pet127 was ascribed as a 5′ to 3′ exoribonuclease according to early genetic experiments. However, the Pet127 protein was difficult to purify due to low solubility. The ortholog from *Talaromyces marneffei* was soluble enough to perform biochemical studies of protein function. Surprisingly, most of the results presented in the paper come from unconventional, pathogenic yeast models *C. albicans* and *T. marneffei*. This shows that we should not be limited to the well-known model systems when investigating molecular phenomena.

**What are some of the landmark moments that provoked your interest in science or your development as a scientist?**

**KLD:** My story is not surprising, without twists in the history. From the moment I started studying biology, I knew that my interests oscillated around science. It was obvious that after completing my PhD, I would decide to work at the University, do research, and teach others.

**MR:** My first laboratory experience during an internship early into my Bachelor's degree assured me that I wanted to become a scientist, but my research interests changed over the years before I decided which path I wanted to follow. I was trying all sorts of different things, from organic chemistry to molecular biology and microfluidics but eventually found a true passion for structural biology.

**If you were able to give one piece of advice to your younger self, what would that be?**

**KLD:** I would advise myself to be patient; experience does not come with knowledge, but with the failures you make. One cannot accelerate the process of getting experience. Another piece of advice would be to keep some enthusiasm until the project's end. A new project is usually exciting, but finishing something requires patience and determination.

**What are your subsequent near- or long-term career plans?**

**MR:** Currently I'm halfway into my postdoc fellowship at EMBL Grenoble and so far it has been a great experience. Since an academic career is also a great chance to travel and explore other cultures, in my next endeavor I would like to do a postdoc in the United States.

**What were the strongest aspects of your collaboration as co-first authors?**

This paper could not be written by one of the groups itself. Our teams complement each other with experience, and only because of that could we complete the project.
