# Supplementary material for: The Pet127 protein is a mitochondrial 5′-to-3′ exoribonuclease from the PD-(D/E)XK superfamily involved in RNA maturation and intron degradation in yeasts
Source: RNA. 2022 May;28(5):711–28. doi: 10.1261/rna.079083.121 (PMC9014873; doi:10.1261/rna.079083.121)
Supplement: Supplemental Material [file supp_28_5_711__DC1.html]

The Pet127 protein is a mitochondrial 5′-to-3′ exoribonuclease from the PD-(D/E)XK superfamily involved in RNA maturation and intron degradation in yeasts — Supplemental Material 

# The Pet127 protein is a mitochondrial 5′-to-3′ exoribonuclease from the PD-(D/E)XK superfamily involved in RNA maturation and intron degradation in yeasts

## Supplemental Material

- Supplemental\_Figure\_S1.pdf
- Supplemental\_Figure\_S2.pdf
- Supplemental\_Figure\_S3.pdf
- Supplemental\_Figure\_S4.pdf
- Supplemental\_Table\_S1.xlsx
- Supplemental\_Table\_S2.docx
