# Supplementary material for: The Pet127 protein is a mitochondrial 5′-to-3′ exoribonuclease from the PD-(D/E)XK superfamily involved in RNA maturation and intron degradation in yeasts
Source: RNA. 2022 May;28(5):711–28. doi: 10.1261/rna.079083.121 (PMC9014873; doi:10.1261/rna.079083.121)
Supplement: Supplemental Material [file supp_079083.121_Supplemental_Figure_S1.pdf]

Supplemental Figure S1. Maximum likelihood phylogenetic tree of Pet127 orthologs listed in Supplemental Table S1. The amino acid sequences were aligned using muscle (Edgar, 2004) and analyzed in SeaView v. 5 (Gouy et al., 2021). For phylogenetic analysis the alignments were trimmed to remove noninformative regions in BMGE (Criscuolo and Gribaldo, 2010), using the BLOSUM30 similarity matrix. Phylogenetic tree was inferred using IQ-TREE (Nguyen et al., 2015) under the LG+F+R3 model. Branch support was assessed using the ultrafast bootstrap approximation (Hoang et al., 2018) with 1000 repeats and the nearest neighbor interchange (NNI) search.

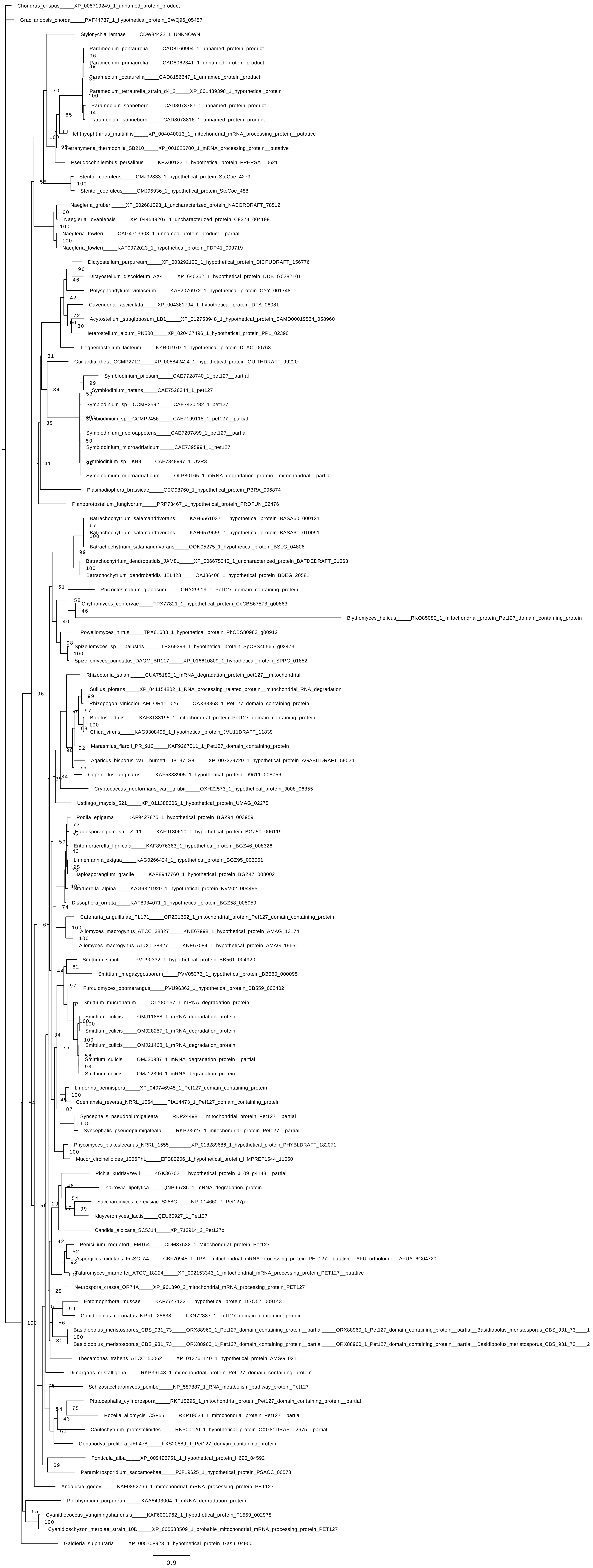
Chondrus_crispus_____XP_005719249_1_unnamed_protein_product
Gracilariopsis_chorda_____PXF44787_1_hypothetical_protein_BWQ96_05457
Stylonychia_lemnae_____CDW84422_1_UNKNOWN
Paramecium_pentaurelia_____CAD8160904_1_unnamed_protein_product
Paramecium_primaurelia_____CAD8062341_1_unnamed_protein_product
Paramecium_octaurelia_____CAD8156647_1_unnamed_protein_product
Paramecium_tetraurelia_strain_d4_2_____XP_001439398_1_hypothetical_protein
Paramecium_sonneborni_____CAD8073787_1_unnamed_protein_product
Paramecium_sonneborni_____CAD8078816_1_unnamed_protein_product
Ichthyophthirius_multifiliis_____XP_004040013_1_mitochondrial_mRNA_processing_protein__putative
Tetrahymena_thermophila_SB210_____XP_001025700_1_mRNA_processing_protein__putative
Pseudocohnilembus_persalinus_____KRX00122_1_hypothetical_protein_PPERSA_10621
Stentor_coeruleus_____OMJ92833_1_hypothetical_protein_SteCoe_4279
Stentor_coeruleus_____OMJ95936_1_hypothetical_protein_SteCoe_488
Naegleria_gruberi_____XP_002681093_1_uncharacterized_protein_NAEGRDRAFT_78512
Naegleria_lovaniensis_____XP_044549207_1_uncharacterized_protein_C9374_004199
Naegleria_fowleri_____CAG4713603_1_unnamed_protein_product__partial
Naegleria_fowleri_____KAF0972023_1_hypothetical_protein_FDP41_009719
Dictyostelium_purpureum_____XP_003292100_1_hypothetical_protein_DICPUDRAFT_156776
Dictyostelium_discoideum_AX4_____XP_640352_1_hypothetical_protein_DDB_G0282101
Polysphondylium_violaceum_____KAF2076972_1_hypothetical_protein_CYY_001748
Cavenderia_fasciculata_____XP_004361794_1_hypothetical_protein_DFA_06081
Acytostelium_subglobosum_LB1_____XP_012753948_1_hypothetical_protein_SAMD00019534_058960
Heterostelium_album_PN500_____XP_020437496_1_hypothetical_protein_PPL_02390
Tieghemostelium_lacteum_____KYR01970_1_hypothetical_protein_DLAC_00763
Guillardia_theta_CCMP2712_____XP_005842424_1_hypothetical_protein_GUITHDRAFT_99220
Symbiodinium_pilosum_____CAE7728740_1_pet127__partial
Symbiodinium_natans_____CAE7526344_1_pet127
Symbiodinium_sp__CCMP2592_____CAE7430282_1_pet127
Symbiodinium_sp__CCMP2456_____CAE7199118_1_pet127__partial
Symbiodinium_necroappetens_____CAE7207899_1_pet127__partial
Symbiodinium_microadriaticum_____CAE7395994_1_pet127
Symbiodinium_sp__KB8_____CAE7348997_1_UVR3
Symbiodinium_microadriaticum_____OLP80165_1_mRNA_degradation_protein__mitochondrial__partial
Plasmodiophora_brassicae_____CEO98760_1_hypothetical_protein_PBRA_006874
Planoprotostelium_fungivorum_____PRP73467_1_hypothetical_protein_PROFUN_02476
Batrachochytrium_salamandrivorans_____KAH6561037_1_hypothetical_protein_BASA60_000121
Batrachochytrium_salamandrivorans_____KAH6579659_1_hypothetical_protein_BASA61_010091
Batrachochytrium_salamandrivorans_____OON05275_1_hypothetical_protein_BSLG_04806
Batrachochytrium_dendrobatidis_JAM81_____XP_006675345_1_uncharacterized_protein_BATDEDRAFT_21663
Batrachochytrium_dendrobatidis_JEL423_____OAJ36406_1_hypothetical_protein_BDEG_20581
Rhizoclosmatium_globosum_____ORY29919_1_Pet127_domain_containing_protein
Chytriomyces_confervae_____TPX77821_1_hypothetical_protein_CcCBS67573_g00863
Blyttiomyces_helicus_____RKO85080_1_mitochondrial_protein_Pet127_domain_containing_protein
Powellomyces_hirtus_____TPX61683_1_hypothetical_protein_PhCBS80983_g00912
Spizellomyces_sp___palustris______TPX69393_1_hypothetical_protein_SpCBS45565_g02473
Spizellomyces_punctatus_DAOM_BR117_____XP_016610809_1_hypothetical_protein_SPPG_01852
Rhizoctonia_solani_____CUA75180_1_mRNA_degradation_protein_pet127__mitochondrial
Suillus_plorans_____XP_041154802_1_RNA_processing_related_protein__mitochondrial_RNA_degradation
Rhizopogon_vinicolor_AM_OR11_026_____OAX33868_1_Pet127_domain_containing_protein
Boletus_edulis_____KAF8133195_1_mitochondrial_protein_Pet127_domain_containing_protein
Chiua_virens_____KAG9308495_1_hypothetical_protein_JVU11DRAFT_11839
Marasmius_fiardii_PR_910_____KAF9267511_1_Pet127_domain_containing_protein
Agaricus_bisporus_var__burnettii_JB137_S8_____XP_007329720_1_hypothetical_protein_AGABI1DRAFT_59024
Coprinellus_angulatus_____KAF5338905_1_hypothetical_protein_D9611_008756
Cryptococcus_neoformans_var__grubii_____OXH22573_1_hypothetical_protein_J008_06355
Ustilago_maydis_521_____XP_011388606_1_hypothetical_protein_UMAG_02275
Podila_epigama_____KAF9427875_1_hypothetical_protein_BGZ94_003959
Haplosporangium_sp__Z_11_____KAF9180610_1_hypothetical_protein_BGZ50_006119
Entomortierella_lignicola_____KAF8976363_1_hypothetical_protein_BGZ46_008326
Linnemannia_exigua_____KAG0266424_1_hypothetical_protein_BGZ95_003051
Haplosporangium_gracile_____KAF8947760_1_hypothetical_protein_BGZ47_008002
Mortierella_alpina_____KAG9321920_1_hypothetical_protein_KVV02_004495
Dissophora_ornata_____KAF8934071_1_hypothetical_protein_BGZ58_005959
Catenaria_anguillulae_PL171_____ORZ31652_1_mitochondrial_protein_Pet127_domain_containing_protein
Allomyces_macrogynus_ATCC_38327_____KNE67998_1_hypothetical_protein_AMAG_13174
Allomyces_macrogynus_ATCC_38327_____KNE67084_1_hypothetical_protein_AMAG_19651
Smittium_simulii_____PVU90332_1_hypothetical_protein_BB561_004920
Smittium_megazygosporum_____PVV05373_1_hypothetical_protein_BB560_000095
Furculomyces_boomerangus_____PVU96362_1_hypothetical_protein_BB559_002402
Smittium_mucronatum_____OLY80157_1_mRNA_degradation_protein
Smittium_culicis_____OMJ11888_1_mRNA_degradation_protein
Smittium_culicis_____OMJ28257_1_mRNA_degradation_protein
Smittium_culicis_____OMJ21468_1_mRNA_degradation_protein
Smittium_culicis_____OMJ20987_1_mRNA_degradation_protein__partial
Smittium_culicis_____OMJ12396_1_mRNA_degradation_protein
Linderina_pennispora_____XP_040746945_1_Pet127_domain_containing_protein
Coemansia_reversa_NRRL_1564_____PIA14473_1_Pet127_domain_containing_protein
Syncephalis_pseudoplumigaleata_____RKP24498_1_mitochondrial_protein_Pet127__partial
Syncephalis_pseudoplumigaleata_____RKP23627_1_mitochondrial_protein_Pet127__partial
Phycomyces_blakesleeanus_NRRL_1555_________XP_018289686_1_hypothetical_protein_PHYBLDRAFT_182071
Mucor_circinelloides_1006PhL_____EPB82206_1_hypothetical_protein_HMPREF1544_11050
Pichia_kudriavzevii_____KGK36702_1_hypothetical_protein_JL09_g4148__partial
Yarrowia_lipolytica_____QNP96736_1_mRNA_degradation_protein
Saccharomyces_cerevisiae_S288C_____NP_014660_1_Pet127p
Kluyveromyces_lactis_____QEU60927_1_Pet127
Candida_albicans_SC5314_____XP_713914_2_Pet127p
Penicillium_roqueforti_FM164_____CDM37532_1_Mitochondrial_protein_Pet127
Aspergillus_nidulans_FGSC_A4_____CBF70945_1_TPA__mitochondrial_mRNA_processing_protein_PET127__putative__AFU_orthologue__AFUA_6G04720_
Talaromyces_marneffei_ATCC_18224_____XP_002153343_1_mitochondrial_mRNA_processing_protein_PET127__putative
Neurospora_crassa_OR74A_____XP_961390_2_mitochondrial_mRNA_processing_protein_PET127
Entomophthora_muscae_____KAF7747132_1_hypothetical_protein_DSO57_009143
Conidiobolus_coronatus_NRRL_28638_____KXN72887_1_Pet127_domain_containing_protein
Basidiobolus_meristosporus_CBS_931_73_____ORX88960_1_Pet127_domain_containing_protein__partial_____ORX88960_1_Pet127_domain_containing_protein__partial__Basidiobolus_meristosporus_CBS_931_73____1
Basidiobolus_meristosporus_CBS_931_73_____ORX88960_1_Pet127_domain_containing_protein__partial_____ORX88960_1_Pet127_domain_containing_protein__partial__Basidiobolus_meristosporus_CBS_931_73____2
Thecamonas_trahens_ATCC_50062_____XP_013761140_1_hypothetical_protein_AMSG_02111
Dimargaris_cristalligena_____RKP36148_1_mitochondrial_protein_Pet127_domain_containing_protein
Schizosaccharomyces_pombe_____NP_587887_1_RNA_metabolism_pathway_protein_Pet127
Piptocephalis_cylindrospora_____RKP15296_1_mitochondrial_protein_Pet127_domain_containing_protein__partial
Rozella_allomycis_CSF55_____RKP19034_1_mitochondrial_protein_Pet127__partial
Caulochytrium_protostelioides_____RKP00120_1_hypothetical_protein_CXG81DRAFT_2675__partial
Gonapodya_prolifera_JEL478_____KXS20889_1_Pet127_domain_containing_protein
Fonticula_alba_____XP_009496751_1_hypothetical_protein_H696_04592
Paramicrosporidium_saccamoebae_____PJF19625_1_hypothetical_protein_PSACC_00573
Andalucia_godoyi_____KAF0852766_1_mitochondrial_mRNA_processing_protein_PET127
Porphyridium_purpureum_____KAA8493004_1_mRNA_degradation_protein
Cyanidiococcus_yangmingshanensis_____KAF6001762_1_hypothetical_protein_F1559_002978
Cyanidioschyzon_merolae_strain_10D_____XP_005538509_1_probable_mitochondrial_mRNA_processing_protein_PET127
Galdieria_sulphuraria_____XP_005708923_1_hypothetical_protein_Gasu_04900
0.9
