# Supplementary material for: The Pet127 protein is a mitochondrial 5′-to-3′ exoribonuclease from the PD-(D/E)XK superfamily involved in RNA maturation and intron degradation in yeasts
Source: RNA. 2022 May;28(5):711–28. doi: 10.1261/rna.079083.121 (PMC9014873; doi:10.1261/rna.079083.121)
Supplement: Supplemental Material [file supp_079083.121_Supplemental_Figure_S2.pdf]

Supplemental Figure S2. Multiple alignment of selected Pet127 ortholog amino acid sequences. Region responsible for the interaction with the mitochondrial RNA polymerase (RPO41) in Saccharomyces cervisiae according to Corbi and Amon (2021) is shaded in yellow

```
                1
Tetrahymena     ---------- ---------- ---------- ---------- ---------- ---------- ----------
Chondrus        ---------- ---------- ---------- ---------- ---------- ---------- ----------
Galdieria       ---------- ---------- ---------- ---------- ---------- ---------- ----------
Naegleria       ---------- ---------- ---------- ---------- ---------- ---------- ----------
Guillardia      ---------- ---------- ---------- ---------- ---------- ---------- ----------
Fonticula       MFRAFLQYSR PRVAQLQHVH LGLSGGLSTP AGAPVGLVQP VLARIGASMG DQSLLTRFGP GRALYSSLPQ
Spizellomyces   ---------- ---------- ---------- ---------- ---------- ---------- ----------
Dictyostelium   ---------- ---------- ---------- ---------- MLSQIKSFNS LNKQIVKPKT FFGGLSSRFG
Saccharomyces   ---------- ---------- ---------- ---------- ---------- ---------- ----------
Phycomyces      ---------- ---------- ---------- ---------- ---------- ---------- ----------
Ustilago        ---------- ---------- ---------- ---------- ---------- ---------- ----------
Suillus         ---------- ---------- ---------- ---------- ---------- ---------- ----------
Candida         ---------- ---------- ---------- ---------- ---------- ---------- ----------
Talaromyces     ---------- ---------- ---------- ---------- ---------- ---------- ----------

               71
Tetrahymena     ---------- ---------- ---------- ---------- ---------- ---------- -MFLRKLLCT
Chondrus        ---------- ---------- ---------- ---------- ---------- ---------- ----------
Galdieria       ---------- ---------- ---------- ---------- ------MPWW RCCAFNSKLR RLSQFYIVRE
Naegleria       ---------- ---------- ---------M LNIFKKRCLS SSGGPGSLLL NSCCHSPSTS VLSTRSIGVV
Guillardia      ---------- ---------- ---------- ---------- ---------- ---------- ------MVGW
Fonticula       SAKVEPQAAG ATRRTPHQGS QQHHHDRRSQ PVNRPAGRSD SGGSPDTGRP RHRPNHTRHN KQRHPSPAGG
Spizellomyces   ---------- ---------- ---------- ---------- ---------- ----MTARAV IHPACRLVLH
Dictyostelium   NRNYTNSINK LFSKNETAPK KSKEREELLN NIFIGAPIQR ISVSDRVRVE VSKVKNIKSA IIEIEDKLDI
Saccharomyces   ---------- ---------- ---------- ---------- --------MG FYNCRFLSRR LSVEPCRIGS
Phycomyces      ---------- ---------- ---------- ---------- ---------- -------MQR IAIRQTTIVW
Ustilago        ---------- ---------- ---------- ---------- ---------- --MSRQSAHD LALLYSLQLV
Suillus         ---------- ---------- ---------- ---------- ---------- -------MHR LQNARSIAIS
Candida         ---------- ---------- ---------- ---------- ---------- ---------- -MIRRNILVI
Talaromyces     ---------- ---------- ---------- ---------- ---------- ---------- --MEYGLFQS

              141
Tetrahymena     QKKF------ ---TNPLLIN SYRFSTQNQQ KGRKRSQNN- ---------- ---------- ----------
Chondrus        ---------- ---------- ---------M SGR------- ---------- ---------- ----------
Galdieria       LRSS---CGN LCREEFFLVN SSKCLFVDKN QKTEGYTQEL KVA------- ---------- ----------
Naegleria       SDSS---KIQ LSSRNFGGTE SAKALFSSSC IGMKKAKSFV TNFERTSSHV NNHT------ ----------
Guillardia      QQED------ ---------- -QTTQAEQAK QGKSSRSELF SLF------- ---------- ----------
Fonticula       TSAA---AGS SAEPGSGVVA GSNPGAGNAP SGTNAHGNRS RHTSGSTPAR QSQHPRRSDG QHARSSTGAP
Spizellomyces   RRSS---SGA AWKHLSEILP SKPPVVAKSV AQKTE----- ---------- ---------- ----------
Dictyostelium   IGRSISKREN FIKNLKFYYD ASKAAVNDFE NNREKYKDNE NEDIYESTEI DPNEPLSYNL PMEKTTIG--
Saccharomyces   AAKS------ ---YQQRSIY HFGAALTNAP SGRETPDKLR SDLHSALEMV D--------- ----------
Phycomyces      SQINPIRSGP KWKTLTRNRI NAVFTNVRFV TSQTNHKNDF SSI------- ---------- ----------
Ustilago        RQLS---TTS SAAGISARLR KQWRGPSRPQ TASASASSSS SSTASSATAS ASSSPETAPS PHSSASSR--
Suillus         ALTC---RRR AFGVSAGVLN ANSDADSNQS QGQSQIVDLG KDKSRSGEMV NEQGKQSGHG QGQEYTFG--
Candida         QGRSFCQSSY LAESLKPKPD IENKVTTNGD SNDITKIALN TNKPGDDG-- ---------- ----------
Talaromyces     PSTS------ -------ISN NASTADSNAP KSNSKAEPLT SDKISFSG-- ---------- ----------

              211
Tetrahymena     ---------- ---------- ---------- ---------- ---------- ---------- ----------
Chondrus        ---------- ---------- ---------- ---------- ---------- ---------- ----------
Galdieria       ---------- --RPEEDDY- ---------- ---------- ---------- ---------- ----------
Naegleria       ---------- --ANRETYT- ---------- ---------- ---------- ---------- ----------
Guillardia      ---------- --RIFRLPE- ---------- ---------- ---------- ---------- ----------
Fonticula       SHHPNHAKTN QAKSNQAKD- ---------- ---------- ---------- ---------- ----------
Spizellomyces   ---------- --DLRKLAA- ---------- ---------- ---------- ---------- ----------
Dictyostelium   ---------- --KIRRLKRL NIELEEKLKV QPVLLEEAKK LYEFVEKELL KSVDKYRDMV FATLPFKPTP
Saccharomyces   ---------- --EIYDTNS- ---------- ---------- ---------- ---------- ----------
Phycomyces      ---------- ---------- ---------- ---------- ---------- ---------- ----------
Ustilago        ---------- --QRHSAFR- ---------- ---------- ---------- ---------- ----------
Suillus         ---------- --KLSRSLR- ---------- ---------- ---------- ---------- ----------
Candida         ---------- --SLKRDDR- ---------- ---------- ---------- ---------- ----------
Talaromyces     ---------- --VLTRDLF- ---------- ---------- ---------- ---------- ----------
```

```
               281
Tetrahymena    ---------- QQNQYAQQQQ QQQQQFQQQQ --------TV TKQEADKQKK SEEDEDIHAK RFAETLKIES
Chondrus       ---------- VLSAASRSAK SSKSTNR--- --------FV RFGYRKQKQE QITLPQAAEQ HGRALPQKSS
Galdieria      ---------- SKIDSSRPVD YVLDAFRSAI --DAAQKAVQ SNPLDGPDSV NKQRSQAEEK KDDARNLSSS
Naegleria      ---------- SHNDSNENNI NFHQPFRKHP --------KN TSYKANHSNN RITHSNNSSS NPEETPILFS
Guillardia     ---------- PLFSYQKSGT GGSE------ --------IC EVKGAGRSSS GQGDSRGTAA EAAAAQWLKE
Fonticula      ---------- STNNSSSASS SNNNSSTSSS -------STS SSSTSSTSSS TSSSSSGNSN NKNTAGAVSS
Spizellomyces  ---------- SFLEASKRSS QGRPQNPQDT -----AD-TV IELLKAHKGD TLSVGAGHAL QHTTVPKEEP
Dictyostelium  KSYSSELEDL LLKESKKLNE DFEDSVKEKP LHSLISSIAS LFRKVETEIE DYTTKVGHLS LNHQKSNLKQ
Saccharomyces  ---------- TVEDIGNKEK GGRQKYTEEM DKAINLLKTN IKKEYRHDKY LERTKVGTYP GRRTYPGRRT
Phycomyces     ---------- NKYNDAKPDV GTKDSDTNVV --------II KNKRRKTARE HLSSQKNSPE SKRGKNKSKG
Ustilago       ---------- ALRQSSRPNV QTSSSVSSSL -------KFI ESARKGKERD SSHPRTASSA ADHSASLIKE
Suillus        ---------- SQRDSSRSNS SVQNPHHDIP -------AAG VSKRRKKEKH SKKKKVGSAG ARPAVESARA
Candida        ---------- VKNLINRLTA TGRHEAKRNP --------TT TNASDAYKLF QNPDKLKALK RMFAQPKVSK
Talaromyces    ---------- KVTNPAEPGE QREQPETAKR --------SK SKKKAKSEKL EEGEQGGETG ENESTKKKSS
```

```
               351
Tetrahymena    TEPG------ ---------- ---------- ---------- ---------- ---------- ----------
Chondrus       IRAR------ ---------- ---------- ---------- ---------- ---------- ----------
Galdieria      RISQ------ ---------- ---------- ---------- ---------- ---------- ----------
Naegleria      FQPTNKIIRN KNSKFKKKSN LSNETRIPSP MNNDIDVSDS LSNSQESSLE PQTGNYGIPQ FKKELEYNVD
Guillardia     HRSS------ ---------- ---------- ---------- ---------- ---------- ----------
Fonticula      SHSAASKPEP QTVGKSPVSA VANSNAPNAG KPDAPARDPS RAKHPHHSTL RRRPRHNHPA GAPAHQASRS
Spizellomyces  LKVN------ ---------- ---------- ---------- ---------L PSRPPTQKP- ----------
Dictyostelium  DIPKENNNNN NNNNNNNNNN NNNNNNNNNN NNNNNNNNNN NNNNNNNNNN NNNNNNNNNN NSDKLNSSQK
Saccharomyces  YPAR------ ---------- ---------- ---------- ---------- ---------- ----------
Phycomyces     FVSRL----- ---------- ---------- ---------- ---------- ---------- ----------
Ustilago       LHAL---DDQ LDALQSQAFS ISQRLRKLAS RRSSSADQLE ETWGPDRLSA PCRAETREN- ----------
Suillus        SLAG---SDA KRAGLLKLLF EGSGETNHAA KDGWTTGWGE DALRP----- ---GENKNP- ----------
Candida        RRVKK----- ---------- ---------- ---------- ---------- ---------- ----------
Talaromyces    KKKKSRKIKR TPGDIKSRMR KVPVKPRA-- ---------- ---------- ---------- ----------
```

```
               421
Tetrahymena    ---------- ---------- ---------- KEGEVEEIKE KRQ------- ---------- ----------
Chondrus       ---------- ---------- ----SDVRKH REFDWDPSLK DVR------- ---------- ----------
Galdieria      ---------- ---------- ---SNYGVES SISTSSSSED DILLQLDLNK LNKIG----- ----------
Naegleria      KLLKQV---- ---------- ---LQKVKTQ KSGEPEENAD TEIDSFSILK QDSDESDIFS LKELNVIDTI
Guillardia     ---------- ---------- ---IKTARKS RDNLSRDTPK RYVPS----- ---------- ----------
Fonticula      RSNPSNPQSG TSSDTVTKAT VSAAARESNS STGTPSPKKH ESAARAQSAP QRAGSAPKSP RTHAFINTSS
Spizellomyces  ---------- ---------- ---SVDHTAG ACDTAPPTPS DGVEQRKPQQ KPRTP----- ----------
Dictyostelium  DNNIKSNNK- ---------- ---KINESQN KKLFSSPPKK DISRNIITKP KVRKPITTKL KTTRMIESRL
Saccharomyces  ---------- ---------- ---------- RTYPASRTYS D--------- ---------- ----------
Phycomyces     ---------- ---------- ---LSALEHD AKENPKPLED HTL------- ---------- ----------
Ustilago       ---------- ---------- ---ESKAIWS ASGSASESSS SSRRTADKSS ADEPKTKLLF GNELLASSNL
Suillus        ---------- ---------- ---GLQGSFS GDGRPSKIKS TGRGQPPHVR HPLPYTRRVE G---------
Candida        ---------- ---------- ---HVKEKFQ KKHTEKNTKT STEPFFK--- ---------- ----------
Talaromyces    ---------- ---------- ---KPPTLTA RQAGKKPSRS KPARNPPGPP KPL------- ----------
```

```
               491
Tetrahymena    ---------- ---------- ---------- ---------- ---------- SSRFIFNSSF NSIQIPLDDV
Chondrus       ---------- ---------- ---------- ---------- ---------- TVDYSSWPEV GNCPAQLV-P
Galdieria      ---------- ---------- ---------- ---------- ---------- LSPWK----- VLDDFSQQLE
Naegleria      EEQEEENIEK PSTKQVDTID LKFLKPQIKF KNFKDEKEVV ESVKLGTSEL KNVVYQPSKG HNYRKDLVDE
Guillardia     ---------- ---------- ---------- ---------- ---------- DVTW------ NALPKPVGFQ
Fonticula      STKSSSSATA PVSTGGAAAP APAPIEGEGV LFKRVPAQAP WMGLGHQGTT QAAWSSAVVS RQEDAGRPHK
Spizellomyces  ---------- ---------- ---------- ---------- ---------- RAKWQ----- TVTPFPFNYQ
Dictyostelium  LQIESEDKSK EISNLNDDKE NSTTENQSLI LDMTGILKRK HKLSDPMDAT MQEFKEKLTF DSNRLNEQEM
Saccharomyces  ---------- ---------- ---------- ---------- ---------- SNSYTFRINV QKIRHALVRY
Phycomyces     ---------- ---------- ---------- ---------- ---------- SSSW------ SQIDNEIVYK
Ustilago       RWRDGKGKLI Q--------- ---------- ---------S LVYSGPFADE QKKWTESITG SVTPKSVPVD
Suillus        ---------- ---------- ---------- ---------- ------LFDD ARDLNAPSSS GPSSINNVLE
Candida        ---------- ---------- ---------- ---------- ---------- DKSTDKYLTS NNIKKHNLEQ
Talaromyces    ---------- ---------- ---------- ---------- ---------- NIEVSDTLVD GLQKEDIDFK
```

```
               561
Tetrahymena    NIINIENEQ- ---------- ---------- ---------- ---------- ---------- ----------
Chondrus       VLSGQDPSI- ---------- ---------- ---------- ---------- ---------- ----------
Galdieria      TVEAPNPEK- ---------- ---------- ---------- ---------- ---------- ----------
Naegleria      PIAPHQKFL- ---------- ---------- ---------- ---------- ---------- ----------
Guillardia     LIEAHGPDA- ---------- ---------- ---------- ---------- ---------- ----------
Fonticula      KKKSLGELAS AGPWFLPEGS AIELLTASEA IPTVENDPAL IDASVAFIES ISLDNLPPEV VKYFDGEEHV
Spizellomyces  AVERTDDAI- ---------- ---------- ---------- ---------- ---------- ----------
Dictyostelium  SDTPPRPLK- ---------- ---------- ---------- ---------- ---------- ----------
Saccharomyces  NQDGVQKHN- ---------- ---------- ---------- ---------- ---------- ----------
Phycomyces     RVEPPREVK- ---------- ---------- ---------- ---------- ---------- ----------
Ustilago       KVAPLREMQ- ---------- ---------- ---------- ---------- ---------- ----------
Suillus        DIDPPSEHK- ---------- ---------- ---------- ---------- ---------- ----------
Candida        FVAPKNED-- ---------- ---------- ---------- ---------- ---------- ----------
Talaromyces    ALDYDTPP-- ---------- ---------- ---------- ---------- ---------- ----------
```

```
              631
Tetrahymena   ---------- ---------- ---------- ---------- --TFHRVPKL MHNLDQVVYS P-GLYKLEDI
Chondrus      ---------- ---------- ---------- ---------- ------IPIL KHGVEAVLRG D-GVYPLEAP
Galdieria     ---------- ---------- ---------- ---------- ------TPRL AEPLRRVLQE D-GVVRVEEL
Naegleria     ---------- -------VEF SDCEYVQLAE THPSEKSYVG NSDVSRIPRL KNGLSRVLTY P-GVHKLYDY
Guillardia    ---------- ---------- ---------- ---------- -----EVPEL AHGLSKIIST KTETPVPLSS
Fonticula     RELLADDAGR ARVARVAEMF AASNGLQYLA ADKFQMGHLD LPKGGDIPVL NPALSHVIHE P-GVHVVRDF
Spizellomyces ---------- ---------- ---------- ---------- ------TPTL AHGLDRVLFN P-GPHFLQDS
Dictyostelium -YRFFPEVCR YLISTTKDNY TFSEDFKEVD YRNKELECVH RHGFDDIPTL KHGLDITVRD R-GLYFTNDF
Saccharomyces ---------- ---------- ---------- ---------- ----QKPPRI GHGLTRVLYQ PLSLQKLRDN
Phycomyces    ---------- ---------- ---------- ---------- ------VPSL AHELERVLFN P-GVHFLKDP
Ustilago      ---------- ---------- ---------- ---------- ------VATL AHGLDRVLFN P-GIYWLRDP
Suillus       ---------- ---------- ---------- ---------- -----PIARL AHGLDRVLFN P-GVHWLQDP
Candida       ---------- ---------- ---------- ---------- ------IARL AHNLDRVLFS P-GVHFLQDP
Talaromyces   ---------- ---------- ---------- ---------- ------VPTL SFGLDRVLFN P-GVYHLRDP

              701
Tetrahymena   ---------- ---------A QLQPDG---- GEFLKTIPQP DDIDFDRIP- ---------- ---PYIPPSN
Chondrus      WIKARGVHNN GRRLRRRASH SENDQYQLYY GDSLRTIVHP DHIAWENIP- ---------- ---EYIPAGS
Galdieria     ---------- ---------Q ENTQEGLDLS SKEEGDVLDR SSFIVENFF- ---------- ---KYIPASK
Naegleria     ---------- ---------K TNTFHF---- HPFLRQLHKP EEINFDNLT- ---------- ---PFIPPSQ
Guillardia    ---------- ---------K FRGAHF---- DSFLKKIAQP DEINWAGIP- ---------- ---PFIPPSQ
Fonticula     ---------- ---------Q SKTMHL---- PPEVRSLEKV KAETLRRMKL NQLIEMKGFK TGLSFTPSSK
Spizellomyces ---------- ---------E TDIFNF---- DPWLKNILQP DDFDYDALP- ---------- ---PYVIASQ
Dictyostelium ---------- ---------- SGKPSF---- SPFLSHIHDF DQLTLST--- ---------- ---NYMRPSE
Saccharomyces ---------- ---------R SRMYNF---- DPAVENI-NP EYLEKKSEKD VNTDSSGEGQ SKPIFITPHK
Phycomyces    ---------- ---------R TKQYNF---- TPFLENITQP SEFNYDAMQ- ---------- ---PYITSSK
Ustilago      ---------- ---------R SGIYNF---- DPRIKDILDV DLFDYAALP- ---------- ---PYVTSSM
Suillus       ---------- ---------R SRVYNF---- TPWLESIPKV TDFAFERVT- ---------- ---GFIRSSQ
Candida       ---------- ---------R TRIYNF---- SPFLKKVIHY KDFNFKAIE- ---------- ---NYTPVSK
Talaromyces   ---------- ---------R SRVYNF---- DPDLGTIMPV TEFDFKTLK- ---------- ---EYITSSK

              771
Tetrahymena   DKLLLDFAKQ S-NIRYVMST STISNVLSQI YFLFSSFRNP NF--DNISS- AYDQ---EPK KYMISQRK-P
Chondrus      DTRLQQIAES TANVHFYSST SSITPAISAL YHLISNFRDT DL--QGRLSS HISD---LPT YFSKMHRR-P
Galdieria     DHKLQRLAKN F-NLRYTGST SSVVPVLSSI YHVLSRYRRT SI--DGLSG- DFRN---STT AFTRYMLK-P
Naegleria     DESLHALMND Y-SCKYQSST SAITSPLVSL YLMLSNFKPT KA--RNLENF KF-----LTS TFTPAIRK-P
Guillardia    DAKLNKLAAE H-GASFQAST SSISGLYSHA YMLLSNWKPV DI--SKFSS- AFRN---MPN QFTSTIRLKP
Fonticula     DKTLERFAVE Q-GVRFIGST SSVSASLSHI YFTMSSNRFI TP--PPLISK SISQ---ETR RFTRATRQ-P
Spizellomyces DSSLLALSKK Q-RKRYVSST SSITGLLSQL YLLISNQKPI NT--SSFSP- CFQE---EPA DFTTTSRA-P
Dictyostelium DKRLLDITKE H-KAKYLSST SSITSILTHL YLSLVKGKAP SS--SPFSPP ILTRLLPLKM GFIPSIVK-P
Saccharomyces DESLLKVAKE H-RKKYISSS SSMTSVLSQL HYLLSNFRRL NIIDSSISK- NFPQ---KNC NYSESAYF-P
Phycomyces    DKNLLEMARD H-NKRYLGST SSVSAVLSHF YFTVSNFRPI DI--SILSK- AFEG---QST KFTRGTRA-P
Ustilago      DPELATLTQR H-QKKYCGST SSMTSLLSQC YFLISGWRNP DV--SGFSA- PFAG---LPS GFSVGAKL-P
Suillus       DDDLHTLAKI H-SRPYVGST SSLTGLLSHI YFLISGGKEV DT--SVLSG- AFAH---EPT NFTPGQRM-P
Candida       HQQLLENSQK F-EKQFYSST SSMTSLLSKF YHFLNKYDRY NV--KRFGPI PFTG---MSN DLPTNLILKP
Talaromyces   DETLIELARK T-KKKYVGSS SSMTSVLSHF HYLLSNWRPL KQ--DIISR- GFED---SDR QFTRLLRG-P

              841
Tetrahymena   TSNML-RKLD PKNGI----- YALDSDSG-- -LFETPNQIL MDLGKVMERQ LTLDPETF-- ----------
Chondrus      VAFTV-KRNS LKKRI----- YSVNAHSG-- --PATGPSIL RDLGHSMERM LTTSPNEFST KY--------
Galdieria     VTIIL---YP GGNGL----- YNIDKAEE-- --KPPKVGVL SDIGHLLEKL FTQENMSVKK RI--------
Naegleria     IVFIL----R PKQNLQGTTF YSLDSYRF-- LFEPRSNQIL LDLGKAMEKL FVMEPEEFEK RF--------
Guillardia    AVVHL----I PHNGI----- MCINAENE-- --KEQRNKIL MDLGKAMERM LTMDKKTFNS LM--------
Fonticula     VLVNL---VR KETGV----- YAVDSSAI-- ---LSTESIL SRMGHVFEKF LTSTTPEFED FLDWHLTHDS
Spizellomyces MSVVL----R HFDGG----- YAIDLEKP-- -DDDDDWNVM MSLGKSMEKM LTVPPAEFEK YH--------
Dictyostelium TSIHL---TP LDGGI----- WAIDTNSG-- -NITQDNTIL MNLGHSMEKM LTLDEEEFNQ KL--------
Saccharomyces SAVIL---RK KRNGI----- CSIDSDRS-- ---LDREIVL SVLGHYLEDF LTEK------ ----------
Phycomyces    ASIYL----R WKNGV----- YAVDVDKS-- --HDVEDTIL SLMGRSMEKV LTLEPEEYNR YL--------
Ustilago      ASIVL----H YKDGY----- YAIDADKTAA GEAENSNYVL TSLGKSMEKM LTATPEEYAK YM--------
Suillus       SSIVL----R YKDGV----- WAIDSNKD-- GEDDSQKNVL TWMGTLLEKF VTVPEPEFKT FL--------
Candida       QGEFMDSTTK EKKPV----- YSIQADNS-- ---CDLDTLL SAMGMCMETL LTNPQNEFVK YH--------
Talaromyces   SAMFL----H YKDGV----- YAIDADKE-- ---FDSANIL MNLGKSMEKQ LTLPKEQFER YR--------

              911
Tetrahymena   ---------R NGLLKNSPVK EQLIDDD--- ------HHRF MKLNKNICLR SQIDCQAIDP KTGQPF-VFE
Chondrus      ---------V RSPNENSSVH HDNMNESKGL ASPDEQFYNY SKISK-FLLR AQIDC--RNE N---TGEVFD
Galdieria     ---------D FSSINIPNVQ DSGSEER--- -PYHNAEYRF AKFGN-CLFR AQVDC--YDP S---TEKFFD
Naegleria     -------VKK YCHTDAISMN EAEV------ -------YRY LRHGS-FIVR SQLDC--YHP K---HG-VFD
Guillardia    -------LKP LGSDEMPEFP EELLLGA--- -------YNF SISNG-IMLR SQLDC--VLG K---NK-IFD
Fonticula     AVDLDSREDR TTPVDLAPPH PSDVHSA--- -------YNY FGAGS-LLLR SQLDC--HDP ENP-ER-IFD
Spizellomyces ---------K VNSTQF--AE KTPQKEA--- -------YHY ATAEG-FLLR AQIDC--HDA RLP-KK-TFD
Dictyostelium ---------L KSNVGDKPVE ---FPDS--- -------YIF SKFGS-LIYR SQLDC--KSD HLPEGRNTFD
Saccharomyces ---------- -------SLK NSSKSEN--- -------YHY SSIDE-FIVR SQLDA--YDP NLPGTG-VFD
Phycomyces    ---------K DNAIEF-PEE EKNLPES--- -------YAY GQIGK-FLLR SQLDC--YDS RLP-RG-TFD
Ustilago      ---------R INSCKL-TQE EKTKPEA--- -------YHY ACTDK-IMMR SQLDC--HDD RLP-RK-TFD
Suillus       ---------R SSPALA-EGE EDKRREA--- -------YRY AKSDR-FLMR SQLDC--VDP RLPGTG-VFD
Candida       ---------K DSGVEF---- NEPLGNT--- -------YNY ASYGD-FLLR SQLDC--YDE RLPGNG-TFD
Talaromyces   ---------R SAPNKITPEE ENEVPES--- -------YHY STLGD-FVMR SQLDA--YDP RLPGSG-MFD
```

```
              981
Tetrahymena   IKTRAVCPIR Y--------- DLNNYLDHID YRINTKFGLH SSFEREYYDL IRG-AMLKYA FQLKIGRMDG
Chondrus      VKTRAVAPIR Y--------- DLQNYEAFST HRLRFLRGKR DSYEREFYDM VRT-VFLKYA LQLRIGRMSG
Galdieria     VKTRALAAIR Y--------- QIERYEDYLE KEITKTTGYE DSFERELYDM IRS-VFIKYA LQFRIGGMSG
Naegleria     IKTRAINRIR I--------- NMEHYEKFVN SKIKLVKGIN NSYEREFYDM VRS-VFVKYS MQARIGDMDG
Guillardia    LKTRATHAIR NCYNILPDIP SEECHLHFTD YRITQQYGLW HSFERELYDM LRS-AFLKYS CQLRIGAMDG
Fonticula     LKTRATLAIR N--------- FVKDYENNVK YRLHRMSGQL SSFEREYYDM IRS-AFMKYS LQVRIGGMEG
Spizellomyces LKTRATVAIR M--------- DPVNYLSNID YKLTSVTGRY QSFESELFDM LRS-AFLKYS FQARIGDMDG
Dictyostelium LKTRSTSSIR N--------- NASTSFNYVS QRISRLSSDK DSFESEYFDL TKS-GAIKYA LQAKIGHMAG
Saccharomyces LKTRAVSAIR Y------DLS HVESNNNQTG YEIDKVYGEF ESLEREYFEL IRS-ALLKYS LQARIGKMDG
Phycomyces    LKTRAAIPIR L--------- DMSNYSDYLG YTLKRSHGLV ESFERDF--- ---------- -QVRIGHMDG
Ustilago      LKTRASIGIR N--------- DRANYVEGSG YQIRRATGVL ESFEREYFDM IRA-AFLKYN FQARIGHMDG
Suillus       VKTRAAVPIR L--------- DLLNFEENSG YLIRTLHGPL ESFEKEYYDL IRS-AFLKYS FQARIGNMDG
Candida       LKTRASSNIR Y--------N SKSGSLEKND YQIWRLNGTY ESYEHEFRDM IRTGAMLKYL FQARIGQMDG
Talaromyces   LKTRAVVSIR M--------- SVSDYEQGLG YEIRNRFGSF ESFEREYYDM IRA-AFLKYS LQVRIGRMDG

             1051
Tetrahymena   AFIAYHNTKE IFGFEYVKTK EIETR----- ---------- -------CFG ---------- ---N--QF--
Chondrus      ALVAYHNTSE ILGLEYLPLK EINSY----- ---------- -------VFG ---------- ---S--EK--
Galdieria     AFVSFHSIGK VSGYQYIPLE EIEKY----- ---------- -------VFG ---------- ---N--EA--
Naegleria     MFLAYHNTRE LFGFEYLKLS EIDSY----- ---------- -------VFG ---------- ---N--SY--
Guillardia    AFVTYHNTQE IFGFQYITLD YMDTL----- ---------- -------LYG ---------- ---S--PQ--
Fonticula     VMVAYHNTDE AFGFQFIPLR DMEQA----- ---------- -------LYG ---------- ---N--AD--
Spizellomyces IFVCYHNTAE VFGFQYLPMG EMDRL----- ---------- -------LFG ---------- ---N--TA--
Dictyostelium IFICYHNTKR IFGSEYLDLE ELEKS----- ---------- -------VFW ---------- ---S--TD--
Saccharomyces IFVAYHNISK MFGFQYLPLD ELDYIIHSSY NSKFDSLLKE KNDITKGIYG EEDYILHYDR DDRKIACL--
Phycomyces    IMVAYHNTSK LFGFQYISRE EMDSR----- ---------- -------IFG ---------- ---T--TK--
Ustilago      IFVAYHTTST IFGFQYIPVE EMNER----- ---------- -------LFG ---------- ---S--QQ--
Suillus       VFVAYHNTAR VFGFQYISLE EMDAR----- ---------- -------LFG QDVKPAGVVN SDSA--PT--
Candida       IFIAYHSINT IFGFQYLPLE ELDKL----- ---------- -------FYT RDTISEFPDV SVRNLNEHRL
Talaromyces   IFVAFHNIER IFGFQYVPLS EMDYA----- ---------- -------LHG ---------- ---QSDTE--

             1121
Tetrahymena   -------FAD ASFVVCSKIL TNLL-DHILN DLE--NEKY- EMLKIGFYSD SP----TKKM IIFV------
Chondrus      -------WGD IAFGTTMHLL QDVL-EKITA EVCQGDP-N- ENLKVVMSTE WS----RLHM YIFV------
Galdieria     -------WAG KSFCSSMKIF EDLL-DNMTH C----FPGE- DPIRVTIDPF SH----TGVL NVYV------
Naegleria     -------MAE RFFTLLLDLL RDVL-DRITD R----FP-N- IPLKITFRPL KVE---PYFI DIFV------
Guillardia    -------MGR FFFDESMKIV SHIL-HEVIQ R----YGQE- HPLRISMQAC KQ----SRRM IIHV------
Fonticula     -------LGD LVFRRGLAIF EQLL-ERISA D----FP-G- QSVALMINSQ I-R---PGHL EILAAGGGAD
Spizellomyces -------YAE QAFALSIKVL QAVL-DVITS K----YR-G- QDIRLTLARE GGE---NTSL AFFA------
Dictyostelium -------MAN RSFLMTNSIL EDLL-NRITE E----MGRE- YRLRVTLSAL TG----HKAS QVFV------
Saccharomyces -------VAN REFKMSMNLF SNIL-KHVEQ LLNSSNTKW- EKCKIMLKTE VEEKR-SKSG RFFN------
Phycomyces    -------MGD EAFRNTLVLF EAVL-DKATA K----YP-D- QTLRLSFEAK ESKNASSTKL NIFV------
Ustilago      -------MAD QAFKLSLGVM EKVL-DAATS M----FV-E- QTLKITVETK EKG---DVGM TVYV------
Suillus       -------RGT RVFEKCVKLL EVVM-DEIVG V----FG-E- RSVKCTFETR E-R---SSKM NIWV------
Candida       PDKLPSLIGE TQFKFSLEIW QKLLQEHILK DLNKEFNNKP TPFRLSVKYD AL----AHLL RVFV------
Talaromyces   -------LGD REFQYSIKLW NKIL-DKATA K----YP-Q- QSLRLHFETT ESR---PARM MVFV------

             1191
Tetrahymena   ELFPEQTKWD EKKNLLKPT- ---------- ------DDVK DEFDYYTKY- ---------- ----------
Chondrus      QRVVDGEQDP FGPSVFL--- ---------- ------EMGE NDTSPAENV- ---------- ----------
Galdieria     ARLREGAATL EWRNI----- ---------- ------QEFA FNKKVEESL- ---------- ----------
Naegleria     EPVQEDKGWS DRSPTEQEKA KW-------- ------ESEK FNANPFADY- ---------- ----------
Guillardia    SRITKYEVTP PVTVGADPTL KIETGQQ--- ------EEFH LFVKSYLND- ----KENKRK WNFGSK----
Fonticula     ERSDEFSGDG LSHRMVEKLL HHCAPTLSPE RQAALQEAVA ASSKPSASVS WCSRPVEPVA AKFVAQ----
Spizellomyces DPVMDFEIME DGG------- ---------- ---------- ---------- ---------- ----------
Dictyostelium EQLIEPDYAH SKIEYQTS-- ---------- ------EELA RAEA------ ---------- ----------
Saccharomyces EPVLNIVALP LSPEYED--- ---------- ------KSLL VKDTSNEQL- ---------- ----------
Phycomyces    EAVPPEEEAK TESDAPL--- ---------- ------LEDN FGTKFFQTH- ---------- ----------
Ustilago      EPA-DASSWS HRQAERKRTI ---------- ------AQLD ISVDRYLDGG L--------- ----------
Suillus       EPVE----WD AEKEGKGNNV AEL------- ------KEVE DETKVAEQG- ------KDGE KDGDRV----
Candida       IPATDEEIDV LQSFPERFKR DFAEDENLGE V-----KKHA RELTKFNEK- ---------- ----------
Talaromyces   QPVTDDDIRA IQEKNKTKIE KAQQQMLYPE TLTQE-EEID LEDDVVESET SEAIADADVA VAEARVGAEN

             1261
Tetrahymena   ---------- -KKLTNKVLK Y-EYQIFPFI NGVLAK---- SNNYSLMPGD ---QI--EVK YKFKK-----
Chondrus      ---------- -TSKQKNLSG IGQWHMDSFL HANWRG---- LSMLGSHPSI --SKLGGHML EPVKEDAALR
Galdieria     ---------- -KILPNDIKK F-QVHIATFV NALYSG---- --APVNYTKN --DQL--HLL YSIREIPV--
Naegleria     ---------- ---IKDTVYC Y-RLSIQTMI NGAVAR---- --GYVDFNRD --DKV--DVL YSLEEMGDTS
Guillardia    ---------- -DQLTVKYAF LKDSECSLEP TSQEQIDEVE EEGVKMKPRP --ADG--AVQ GKAANNGKVT
Fonticula     ---------- -DHSNLQFKL Y-SVQMESFL DGASIG-ANI PPSTSTQPFT --GQW--DLF FGIHERTPAD
Spizellomyces ---------- -YVSPPELSK F-ELQCRSIL NGAEEP---- SYFDIAADGS --DTW--ELQ YKIKQ-----
Dictyostelium ---------- -KQITNPVTM Y-ELKIDSLI NEKKVL---- --GSLHFSEN --DNV--RCF YRIVKPDH--
Saccharomyces ---------- -TEELLNLRS YNENLLEEHL NSLVGFKVNV KHFYHHHPNT --THL---PD FALKKNDILD
Phycomyces    ---------- ----DPTLDP YSKLTMFTMS TESYVNGKQI SDYVNLRRDT --DEW--SVQ YQIDETNKDE
Ustilago      ---------- -MLGPVDFSY LPGRRIDPLS DDEIER---- -RMRASLPAV ---RW--EID YSISPRPDLT
Suillus       ---------- -KTEEPPIVQ I-DVEVQNFV TGQPVW---G SKAIGVTPND ---NW--ILH WTVSHSSLET
Candida       ---------- -CLETKEVFS Y-VIQMDSCM IDGKIR---- --QYHQLPRD YFKDW--QLI YNIKRTSNAP
Talaromyces   ESIAADTTTI EKIESQPVLK TETGSIIDPT TTISTSELPS QPPQKDLPGL --ITA--LAK YIHNSRHIVD
```

```
              1331
Tetrahymena    ---------- ---------- ---------- ---------- ---------- ---------- ----------
Chondrus       ---------- ---------- ---------- ---------L AKRPRSETYD YSNYDLTSLT RDRFRVWEL-
Galdieria      ---------- ---------- ---------- ---------- ---------- ---------- ----------
Naegleria      ---------- ---------- ---------- ---------- ---------- ---------- ----------
Guillardia     ---------- ---------T GVQPSSGPKN FEQASKGKEV KSQQALEQDR KLLLNLKGLV NVQPVDLEKK
Fonticula      -----ALRSH FHAFVKNVSM SRDAGLFFRS LEAQQIPDDI WNSDIPVACI SNDPNAPGSS FVHRNKNFLK
Spizellomyces  ---------- ---------- ---------- ---------- ---------- ---------- ----------
Dictyostelium  ---------- ---------- ---------- ---------- ---------- ---------- ----------
Saccharomyces  ---TESRKYI SDMMKRDWYK DIPSTQTPNF FHASDVSTWE VNSTFTDIND KQILRKLYFK YLDVKLNALK
Phycomyces     ---------- ---------- ---------- ---GHVESMF RSLRRTQSNI FSASKYPIPI L---------
Ustilago       ESKIRSNLEA VRAKQASLNS LILPNVEALN AREQYRTEQL AKNPDALQRF LQERKSGVAA GMPLAPGQLS
Suillus        SRIRANLASA KDRQFRAWNF PANISGPEEM ETWWNALDFG RRKTAVSSSG EG--EEITLS RLQDLVAGVE
Candida        ---------- ---------- ---------- ---------- ------KKYV SNLLRFPDTK ISQRL-----
Talaromyces    ------NITE ILATTNQKGD TNKPALSPLT VLSEKIETSR KVVQSVKQKI DNILEEREAA YIRPKLFGME

              1401
Tetrahymena    ---------- ---------- ---------- ------IGKP AFLDYMNFLH EAYKFETMNL DLSYIGAWV-
Chondrus       ---------- ---------- ---------- ------RVAP LVNDKMAPRN LICLGEEDRF RLKYNLKEVS
Galdieria      ---------- ---------- ---------- ------CLND YIKALRLSLG LEDMPQSRHF SVSGLSSTEL
Naegleria      ---------- ---------- ---------- ------TDKF LMNEYVLTLK KAFFIEQ--- ----------
Guillardia     AEPTNVVMNE NVEQQASNLS DKGNS----- ------RGAA KQRISQAAGK QTNLKEGDQQ TISQRGRRRV
Fonticula      GNGARWQGSR RPDRRTKVFA PPGLEGKARI ------KSVD QFRDSLSSVR MARNAEGTSP ADASAAAAAA
Spizellomyces  ---------- ---------- ---------- ------ANIS LMQLYDEYER LRNRCSNLKP FVPTKGHKIF
Dictyostelium  ---------- ---------- ---------- ------TQDE IKYHYALALK ASKLYSQFSK IQKSRN----
Saccharomyces  NQVITRQEPD MSK------- ---------- ------KDEI MNRIKSLQAR NDHRDNGSNK RYSNFGPTRL
Phycomyces     ---------- ---------- ---------- ------KQLK SLSEKQLKME RQSRKTGNSK SIEPND----
Ustilago       AENAASQSPS TPPPAETSDA SSSNTVLPDD VLKPIEPNDT SPRRKQAESR FVRMRNARTL RLRELAKLGA
Suillus        SPVVTVEENG LVEEEGPKAN ENGVPSFNRK LFRPASTSIE ILRQLSRDGR EETLRAEAEE EAMGREKIVW
Candida        ---------- ---------- ---------- ------KKVH QLYEKIGSIR KKSWQEKDKK SQVYHPKFKF
Talaromyces    IGIQNLVNNR VVDRPTNLKP TNDWKVVYT- ------INEF KIGDGGLWNH FVGMRSRREK ALSFEKRKSS

              1471
Tetrahymena    ---------- ---------- ---------- ---------- ------
Chondrus       DVKTEHVAKF ITSLGRIYTY ---------- ---------- ------
Galdieria      ADILASTSFQ AFHLIK---- ---------- ---------- ------
Naegleria      ---------- ---------- ---------- ---------- ------
Guillardia     NSQGNAAITG NHGRDNRGSY TAKNSVQGRK PVTHQQMQGG QRSS--
Fonticula      AALATAVATA TAATSAAASI PPAAPVAVAA HSS------- ------
Spizellomyces  VNRLRKKANL DLY------- ---------- ---------- ------
Dictyostelium  ---------- ---------- ---------- ---------- ------
Saccharomyces  QTKLRAYAKK GALRRKLLER SNKFHI---- ---------- ------
Phycomyces     ---------- ---------- ---------- ---------- ------
Ustilago       KDANHNKSLD KLILYQPRS- ---------- ---------- ------
Suillus        GLVGNGGIVN SETKEVPEDG SSVSTITQSS EMGTVDAVEQ TSQEKE
Candida        ---------- ---------- ---------- ---------- ------
Talaromyces    DDHYILNLRR LAMQGKKFRE EEDRIDKERG IVVYEELKGG DSAGSA
```
