# Supplementary material for: The Pet127 protein is a mitochondrial 5′-to-3′ exoribonuclease from the PD-(D/E)XK superfamily involved in RNA maturation and intron degradation in yeasts
Source: RNA. 2022 May;28(5):711–28. doi: 10.1261/rna.079083.121 (PMC9014873; doi:10.1261/rna.079083.121)
Supplement: Supplemental Material [file supp_079083.121_Supplemental_Figure_S3.pdf]

Supplemental Figure S3. Coverage of the *C. albicans* mtDNA reference sequence with one of two identical copies of the inverted repeat region removed by forward and reverse RNA-seq reads in wild-type (BWP17, WT), homozygous *pet127Δ* strain (KO), and two independent isolates of *Capet127*$_{D375A}$/ *Capet127*$_{D375A}$ point mutant strain (mut3, mut4). Forward (fwd) and reverse (rev) mapping reads are shown separately. Note that for TU02 and TU04 the sense strand is the reverse strand. Transcription units and gene annotations are according to Kolondra et al. 2015. BWA files obtained using bamCompare were visualized in pyGenomeTracks. The maximum value on the depth coverage axis was set to better visualize low-coverage regions, truncating the highest values. General coverage graphs for the entire mtDNA are followed by detailed graphs for selected regions.

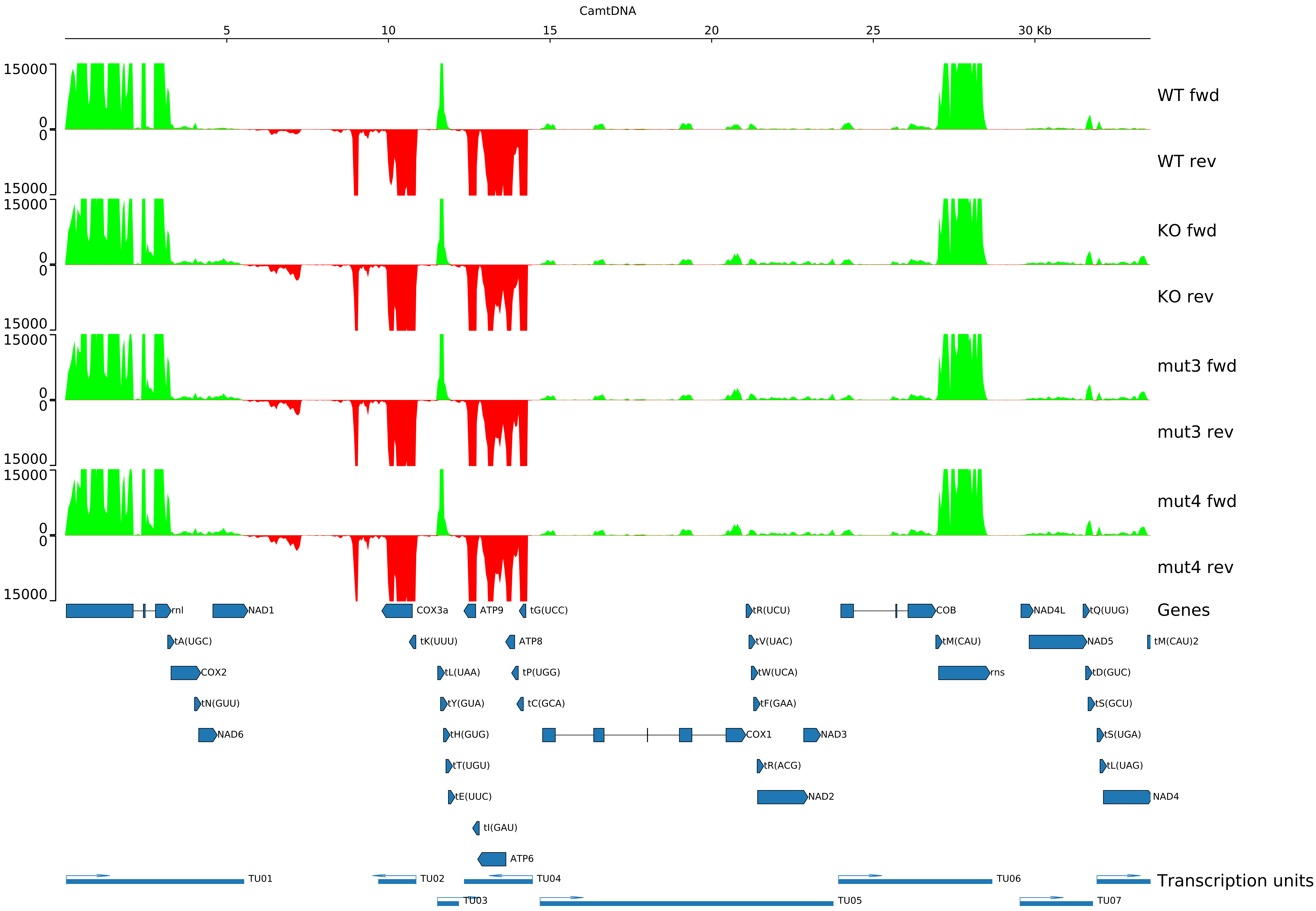

CamtDNA
5
10
15
20
25
30 Kb
15000
0
WT fwd
WT rev
KO fwd
KO rev
mut3 fwd
mut3 rev
mut4 fwd
mut4 rev
Genes
rnl
NAD1
COX3a
ATP9
tG(UCC)
tR(UCU)
COB
NAD4L
tQ(UUG)
tA(UGC)
tK(UUU)
ATP8
tV(UAC)
tM(CAU)
NAD5
tM(CAU)2
COX2
tL(UAA)
tP(UGG)
tW(UCA)
rns
tD(GUC)
tN(GUU)
tY(GUA)
tC(GCA)
tF(GAA)
tS(GCU)
NAD6
tH(GUG)
COX1
NAD3
tS(UGA)
tT(UGU)
tR(ACG)
tL(UAG)
tE(UUC)
NAD2
NAD4
tI(GAU)
ATP6
Transcription units
TU01
TU02
TU04
TU06
TU03
TU05
TU07

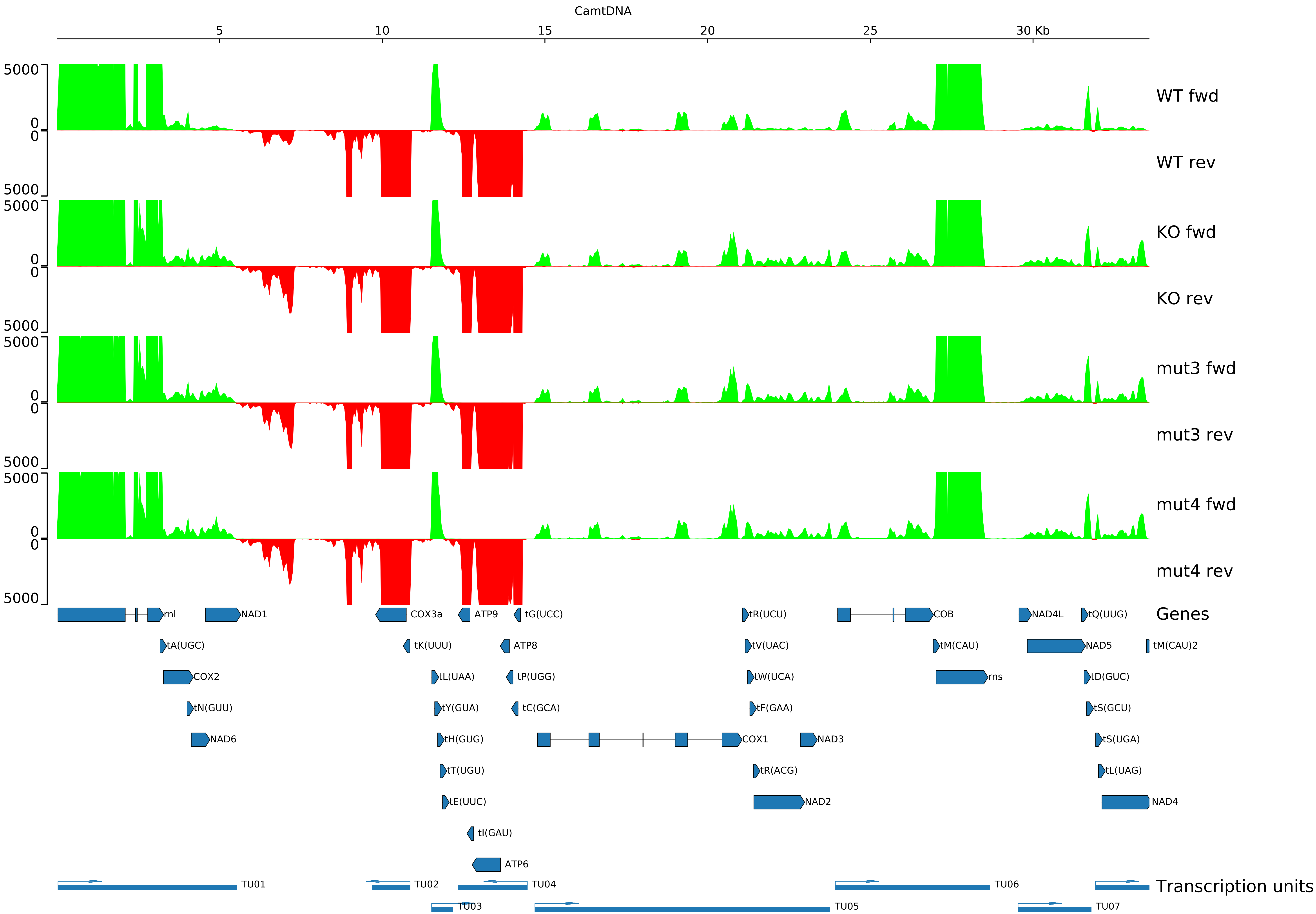

CamtDNA
5
10
15
20
25
30 Kb
5000
0
WT fwd
WT rev
KO fwd
KO rev
mut3 fwd
mut3 rev
mut4 fwd
mut4 rev
Genes
rnl
NAD1
COX3a
ATP9
tG(UCC)
tR(UCU)
COB
NAD4L
tQ(UUG)
tA(UGC)
tK(UUU)
ATP8
tV(UAC)
tM(CAU)
NAD5
tM(CAU)2
COX2
tL(UAA)
tP(UGG)
tW(UCA)
rns
tD(GUC)
tN(GUU)
tY(GUA)
tC(GCA)
tF(GAA)
tS(GCU)
NAD6
tH(GUG)
COX1
NAD3
tS(UGA)
tT(UGU)
tR(ACG)
tL(UAG)
tE(UUC)
NAD2
NAD4
tI(GAU)
ATP6
Transcription units
TU01
TU02
TU04
TU06
TU03
TU05
TU07

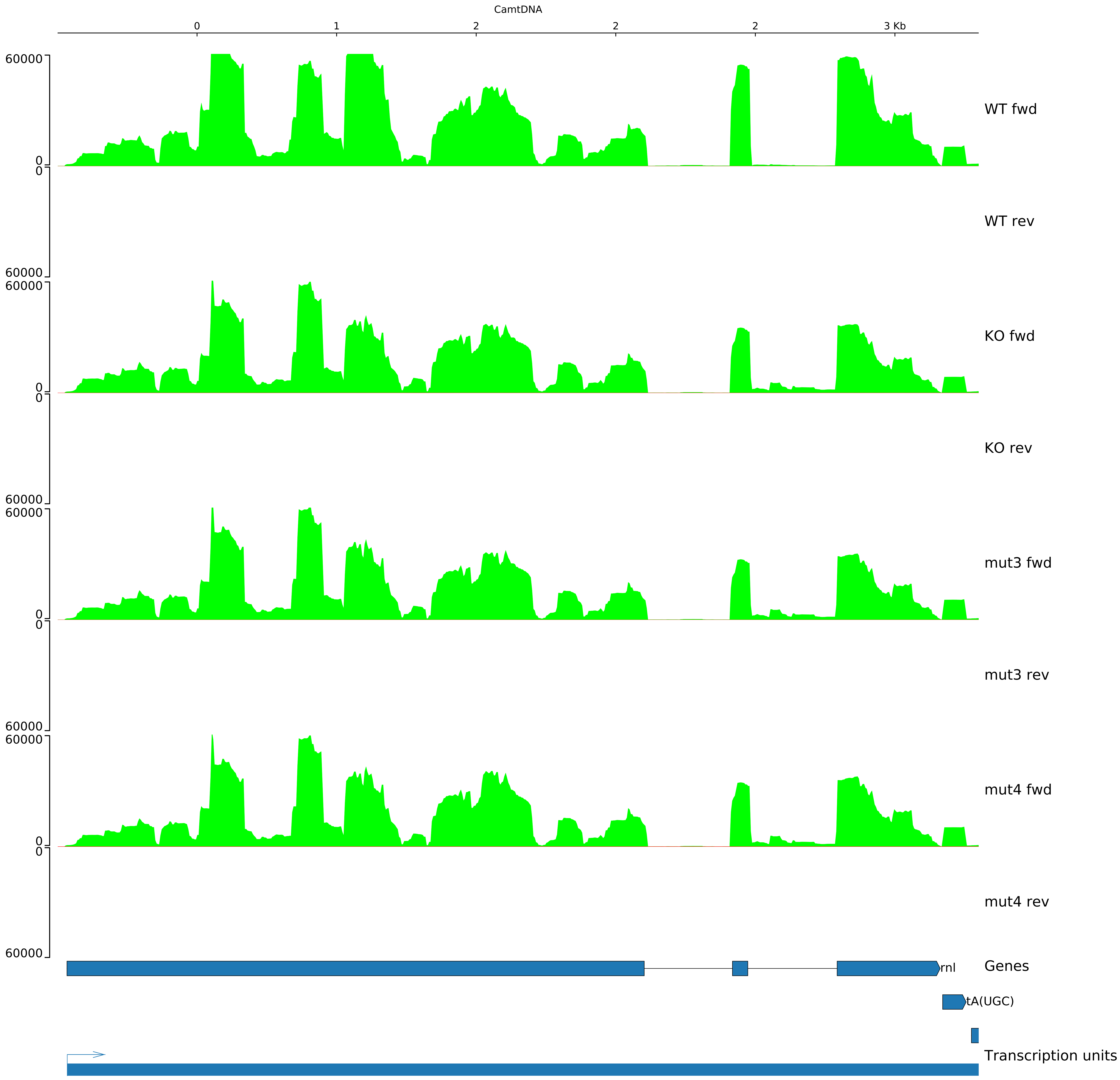
CamtDNA
0
1
2
2
2
3 Kb
60000
0
WT fwd
WT rev
60000
KO fwd
KO rev
mut3 fwd
mut3 rev
mut4 fwd
mut4 rev
Genes
rnl
tA(UGC)
Transcription units

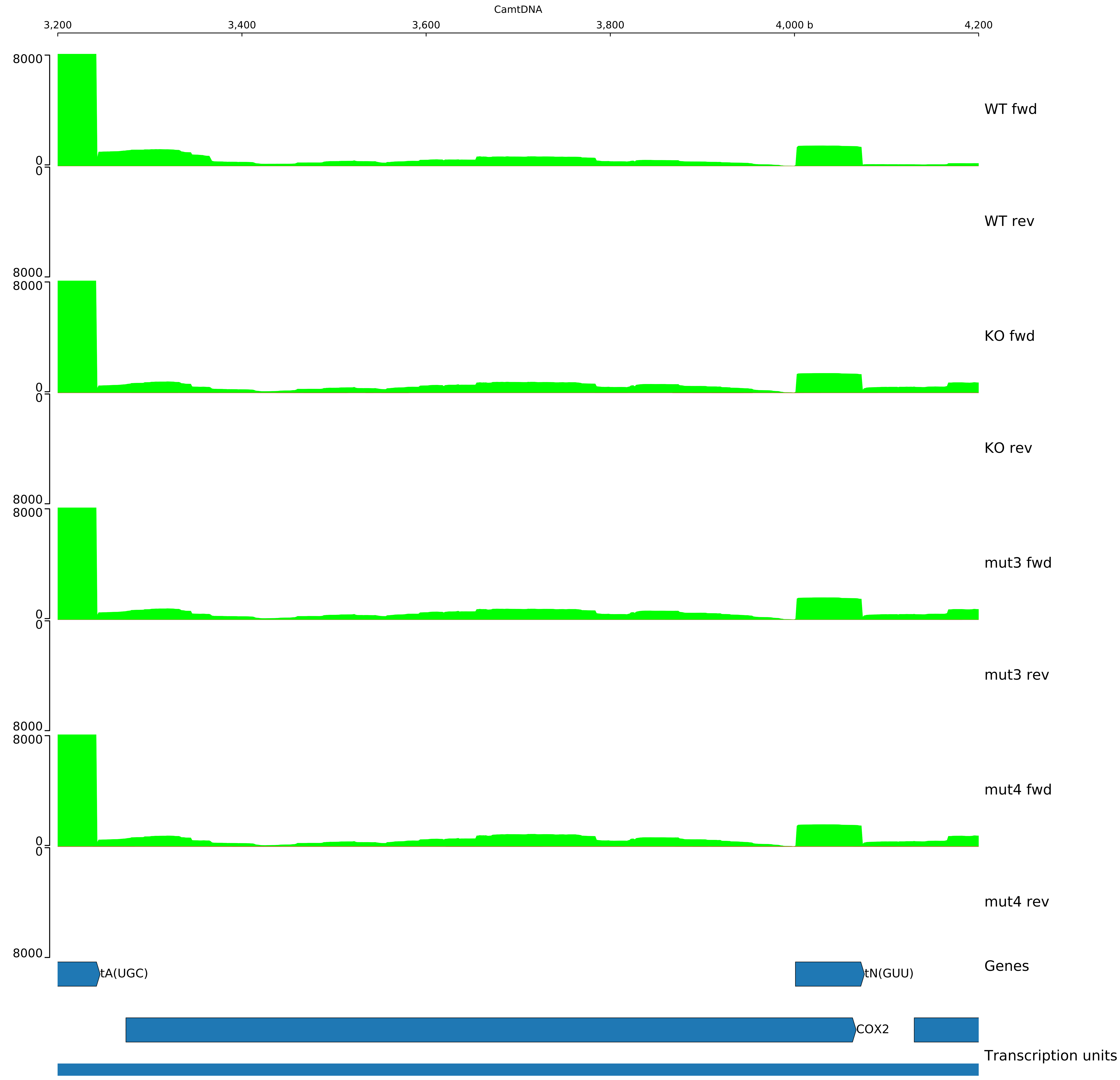
CamtDNA
3,200
3,400
3,600
3,800
4,000 b
4,200
8000
0
WT fwd
WT rev
KO fwd
KO rev
mut3 fwd
mut3 rev
mut4 fwd
mut4 rev
Genes
tA(UGC)
tN(GUU)
COX2
Transcription units

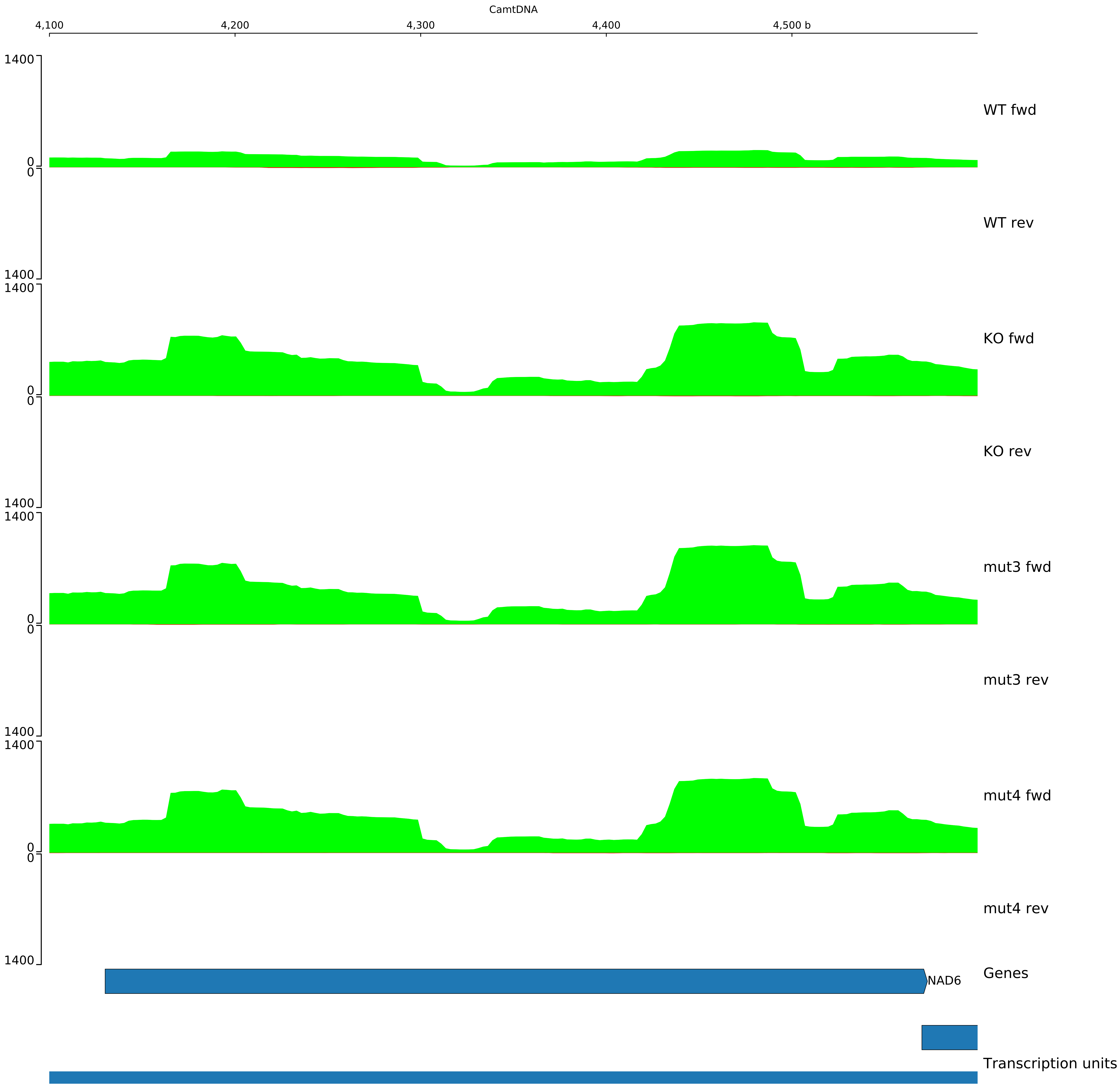
CamtDNA
4,100
4,200
4,300
4,400
4,500 b
1400
0
WT fwd
0
WT rev
1400
1400
0
KO fwd
0
KO rev
1400
1400
0
mut3 fwd
0
mut3 rev
1400
1400
0
mut4 fwd
0
mut4 rev
1400
Genes
NAD6
Transcription units

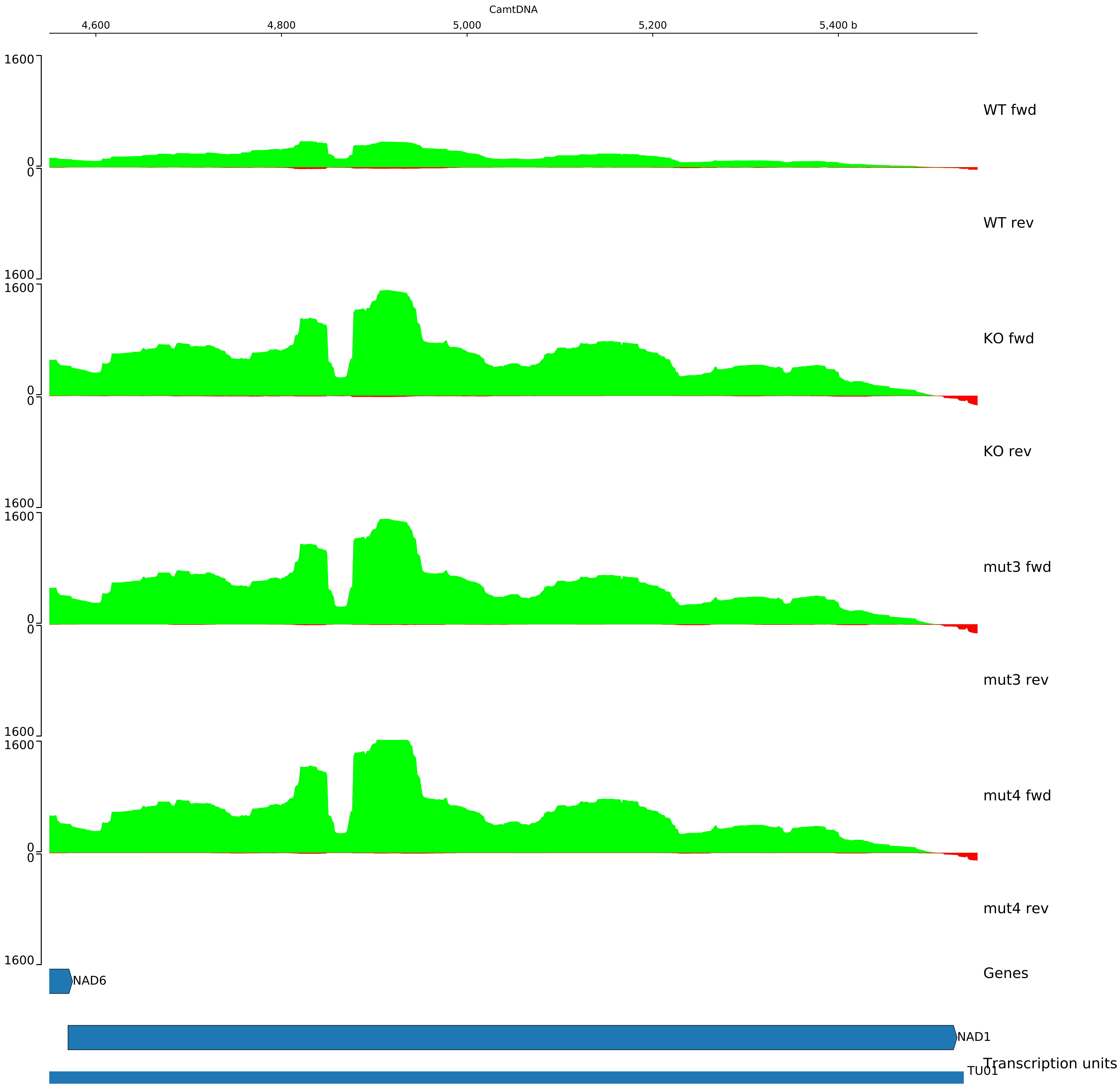

CamtDNA
4,600
4,800
5,000
5,200
5,400 b
1600
0
WT fwd
WT rev
KO fwd
KO rev
mut3 fwd
mut3 rev
mut4 fwd
mut4 rev
Genes
NAD6
NAD1
Transcription units
TU01

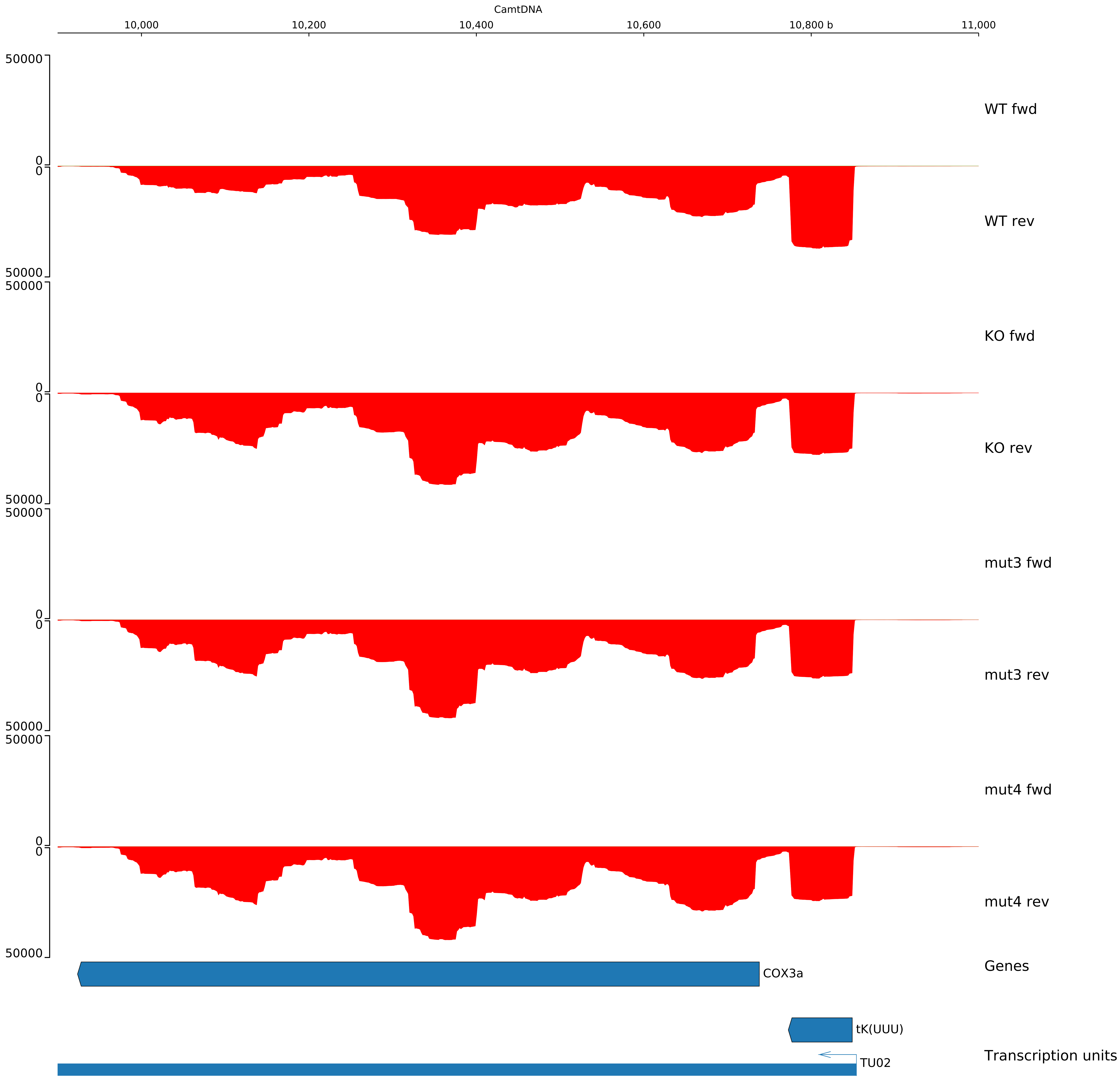
CamtDNA
10,000
10,200
10,400
10,600
10,800 b
11,000
50000
0
0
50000
WT fwd
WT rev
KO fwd
KO rev
mut3 fwd
mut3 rev
mut4 fwd
mut4 rev
Genes
COX3a
tK(UUU)
Transcription units
TU02

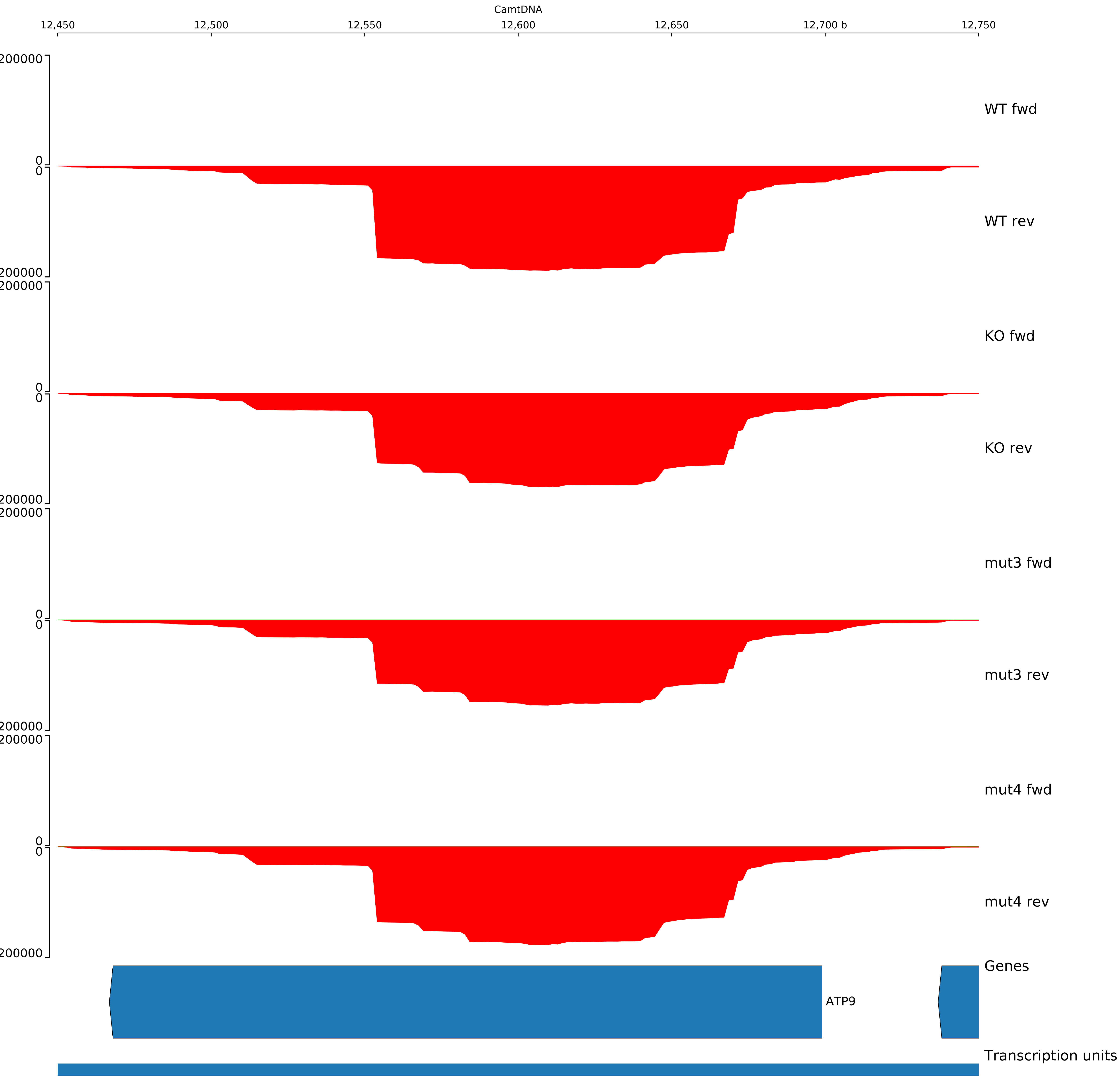

CamtDNA
12,450
12,500
12,550
12,600
12,650
12,700 b
12,750
200000
0
0
200000
WT fwd
WT rev
KO fwd
KO rev
mut3 fwd
mut3 rev
mut4 fwd
mut4 rev
Genes
ATP9
Transcription units

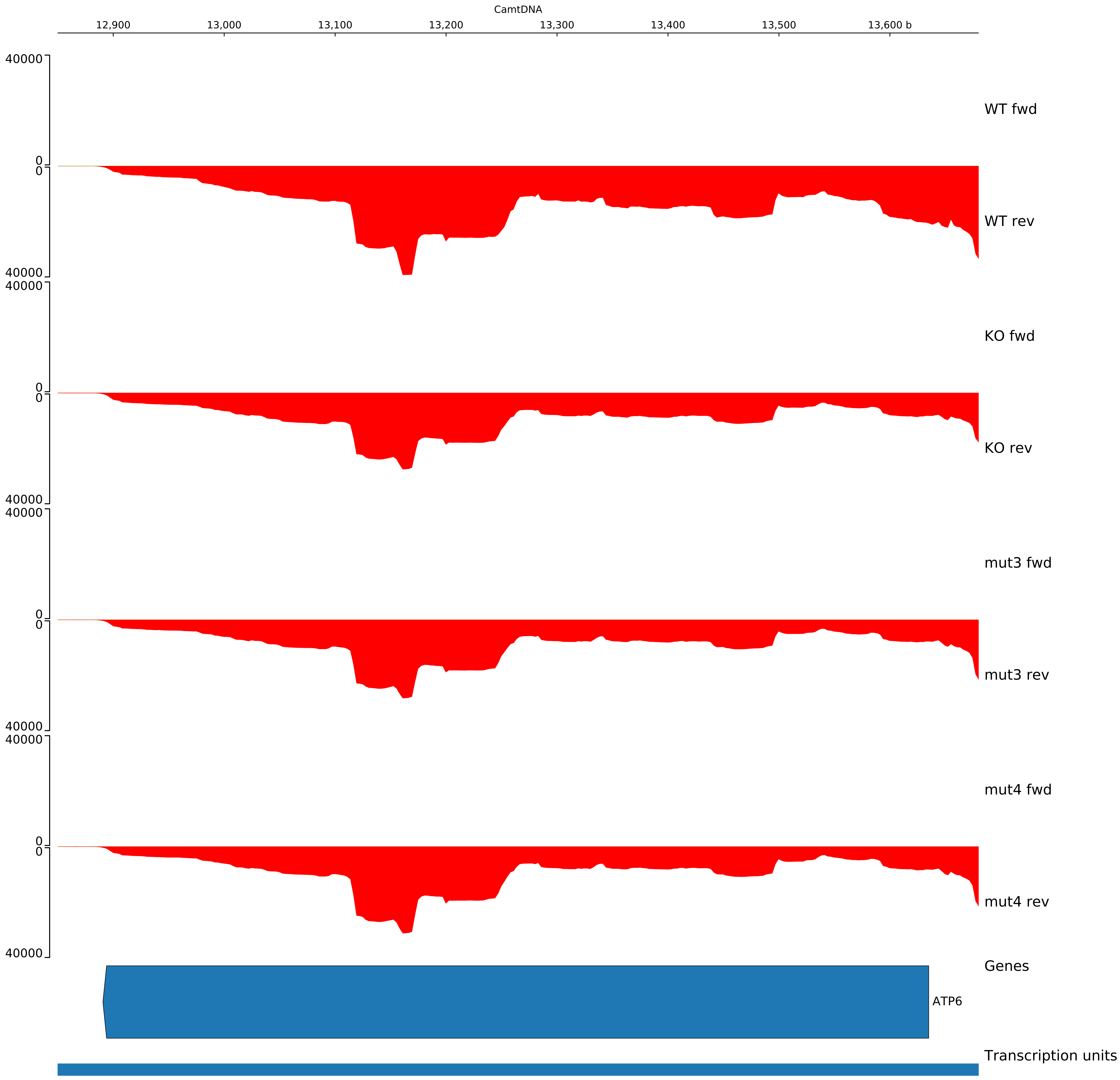

CamtDNA
12,900
13,000
13,100
13,200
13,300
13,400
13,500
13,600 b
40000
0
WT fwd
WT rev
KO fwd
KO rev
mut3 fwd
mut3 rev
mut4 fwd
mut4 rev
Genes
ATP6
Transcription units

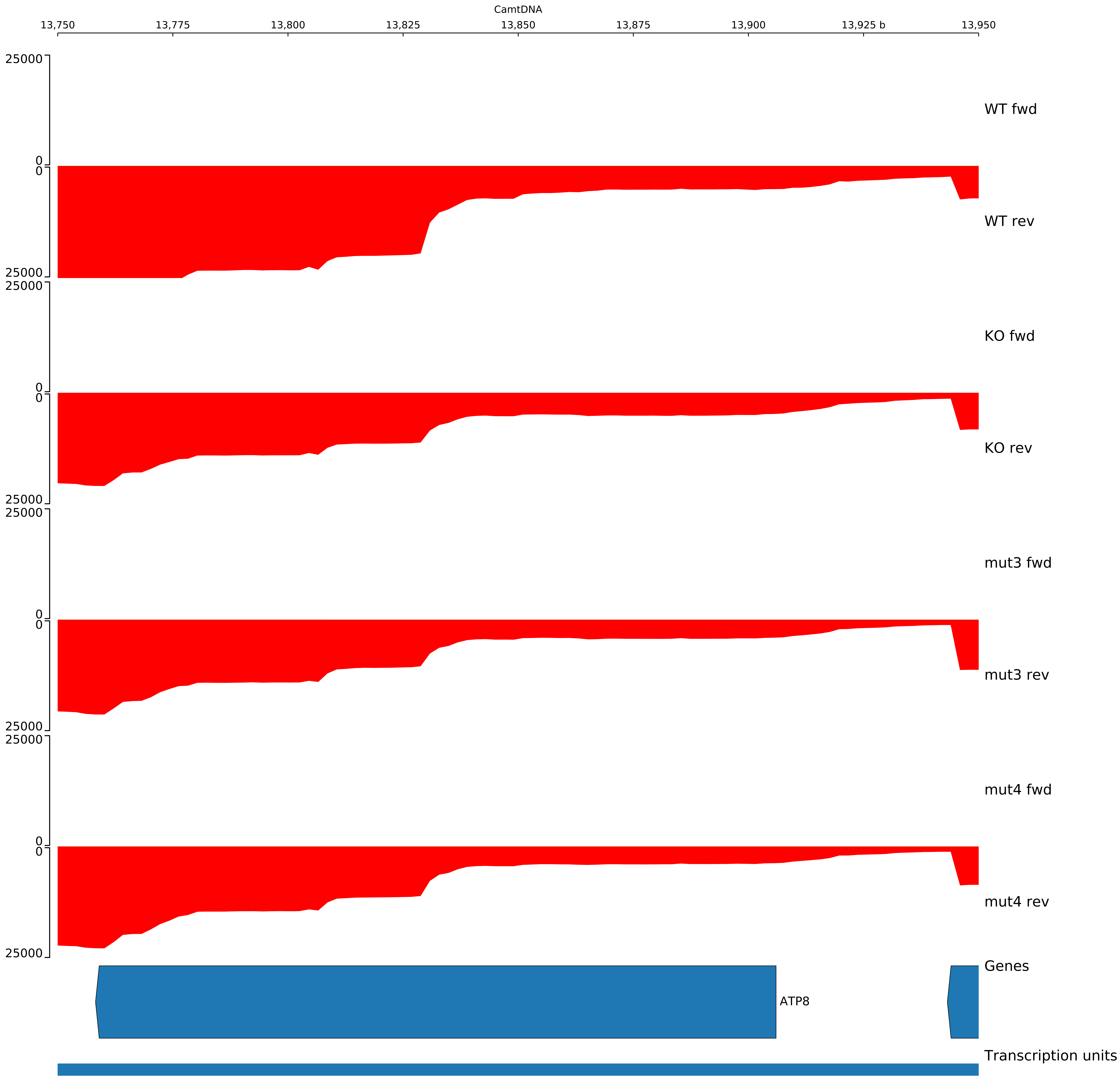
CamtDNA
13,750
13,775
13,800
13,825
13,850
13,875
13,900
13,925 b
13,950
25000
0
WT fwd
WT rev
KO fwd
KO rev
mut3 fwd
mut3 rev
mut4 fwd
mut4 rev
Genes
ATP8
Transcription units

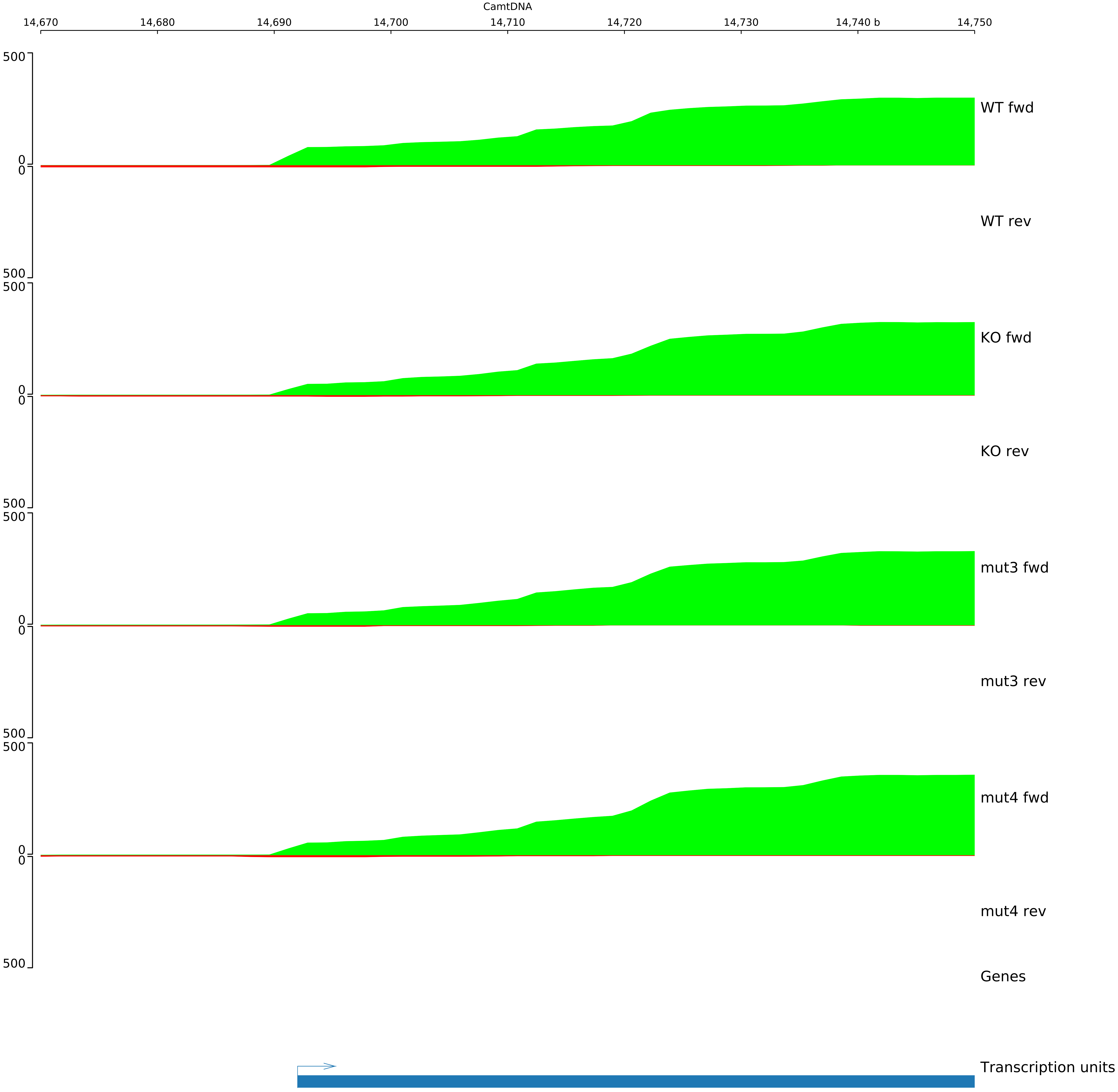
CamtDNA
14,670
14,680
14,690
14,700
14,710
14,720
14,730
14,740 b
14,750
500
0
WT fwd
WT rev
KO fwd
KO rev
mut3 fwd
mut3 rev
mut4 fwd
mut4 rev
Genes
Transcription units

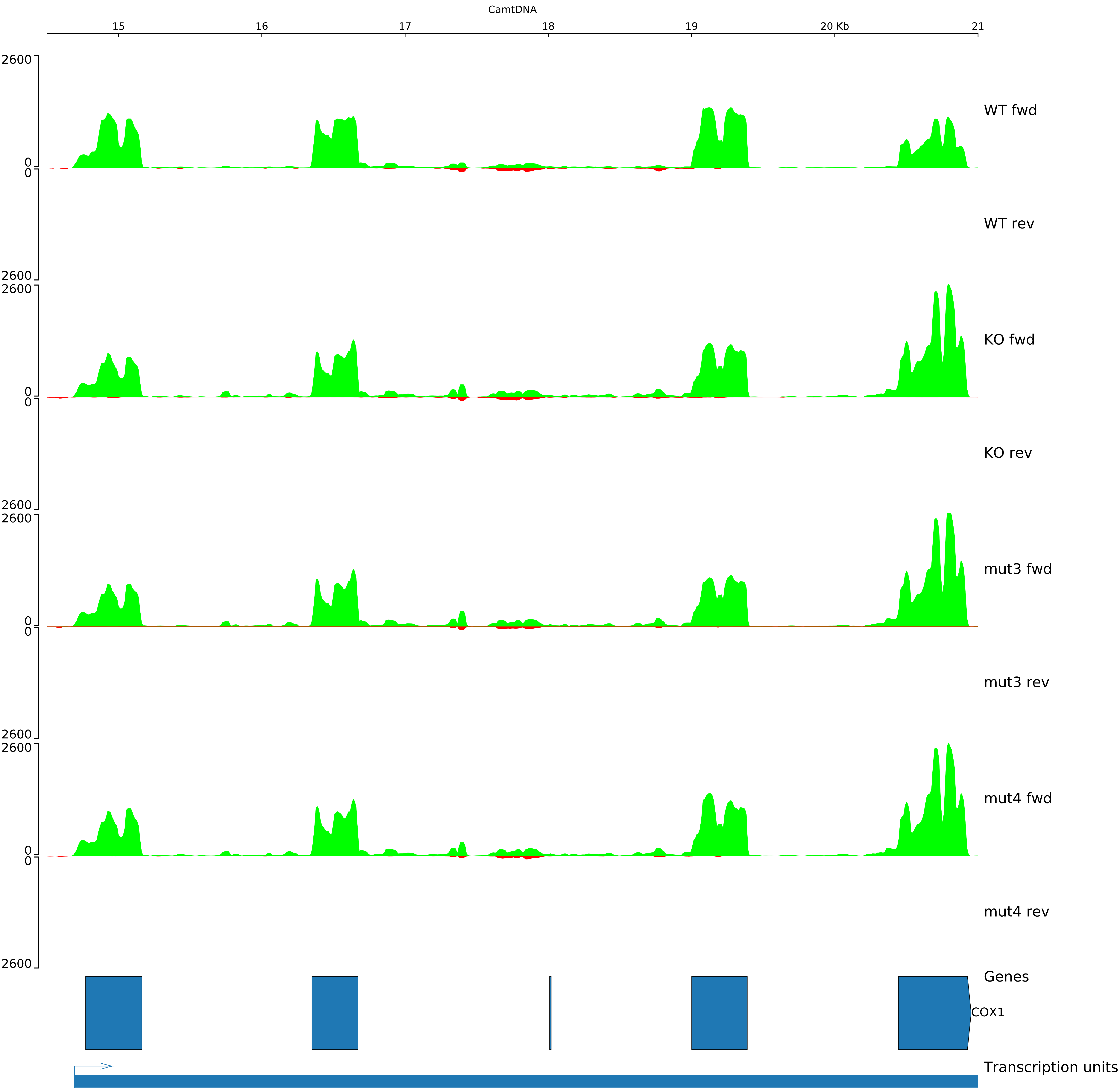

CamtDNA
15
16
17
18
19
20 Kb
21
2600
0
WT fwd
WT rev
KO fwd
KO rev
mut3 fwd
mut3 rev
mut4 fwd
mut4 rev
Genes
COX1
Transcription units

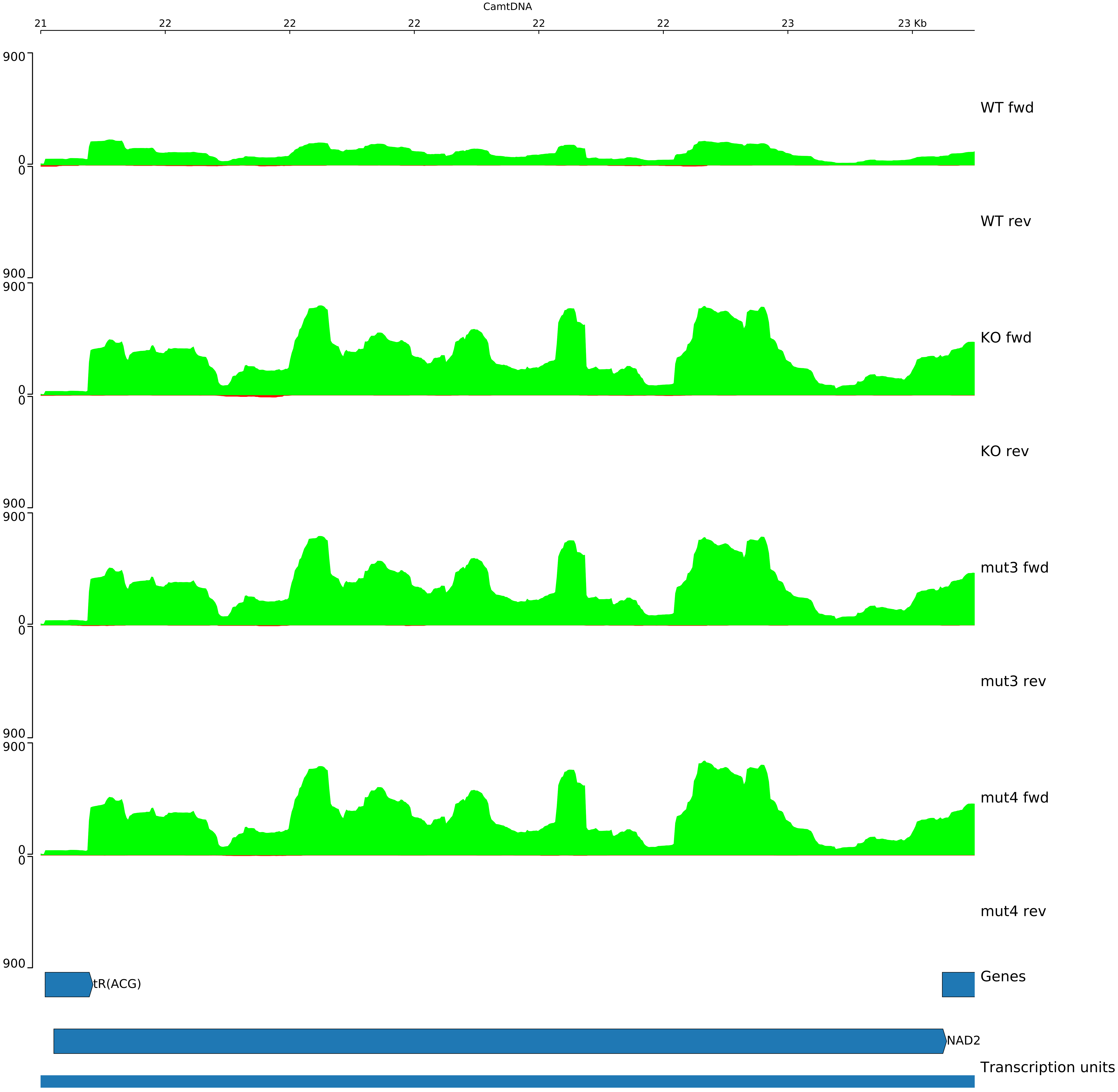
CamtDNA
21
22
22
22
22
22
23
23 Kb
900
0
WT fwd
WT rev
KO fwd
KO rev
mut3 fwd
mut3 rev
mut4 fwd
mut4 rev
Genes
tR(ACG)
NAD2
Transcription units

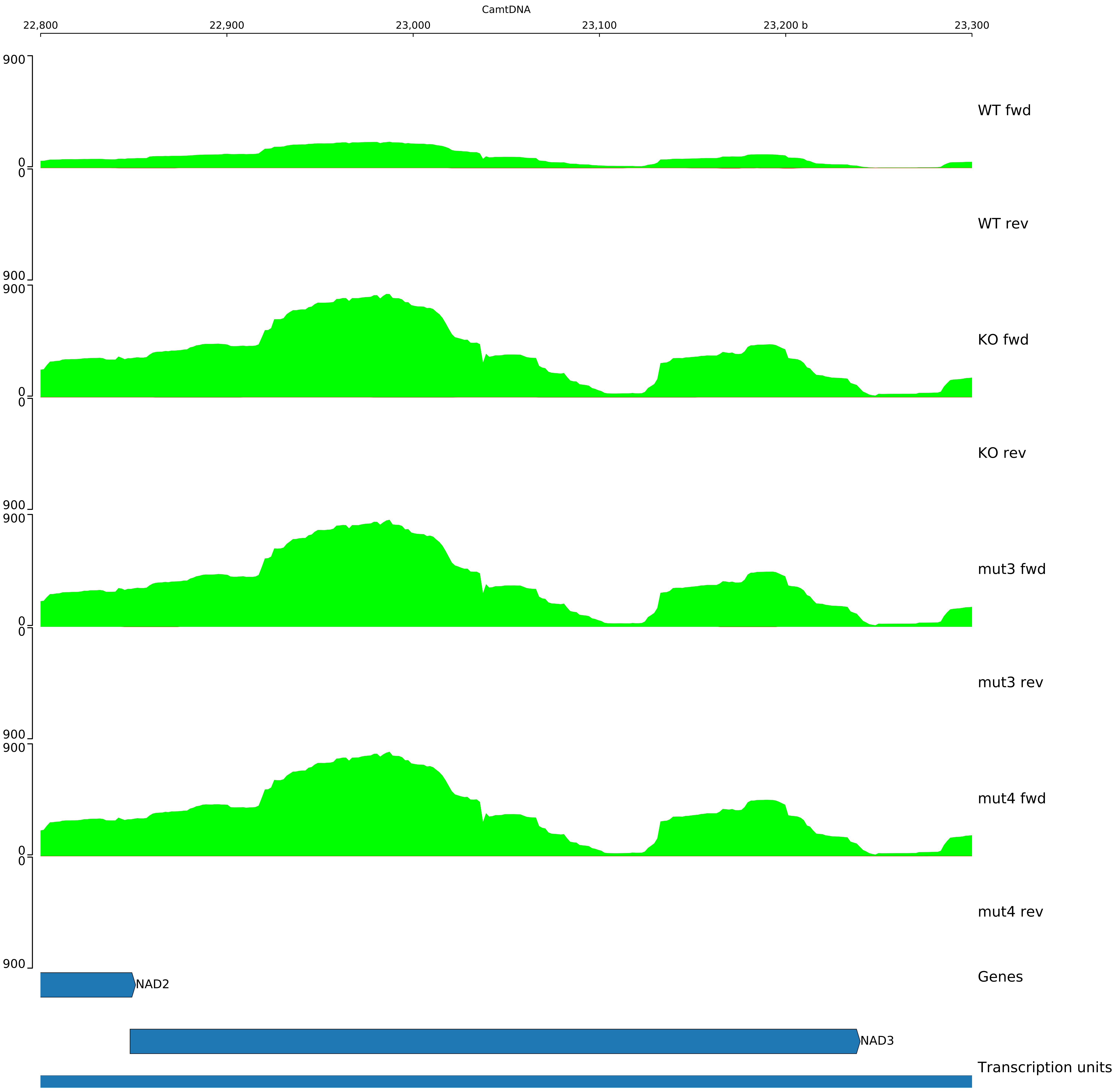

CamtDNA
22,800
22,900
23,000
23,100
23,200 b
23,300
900
0
WT fwd
WT rev
KO fwd
KO rev
mut3 fwd
mut3 rev
mut4 fwd
mut4 rev
Genes
NAD2
NAD3
Transcription units

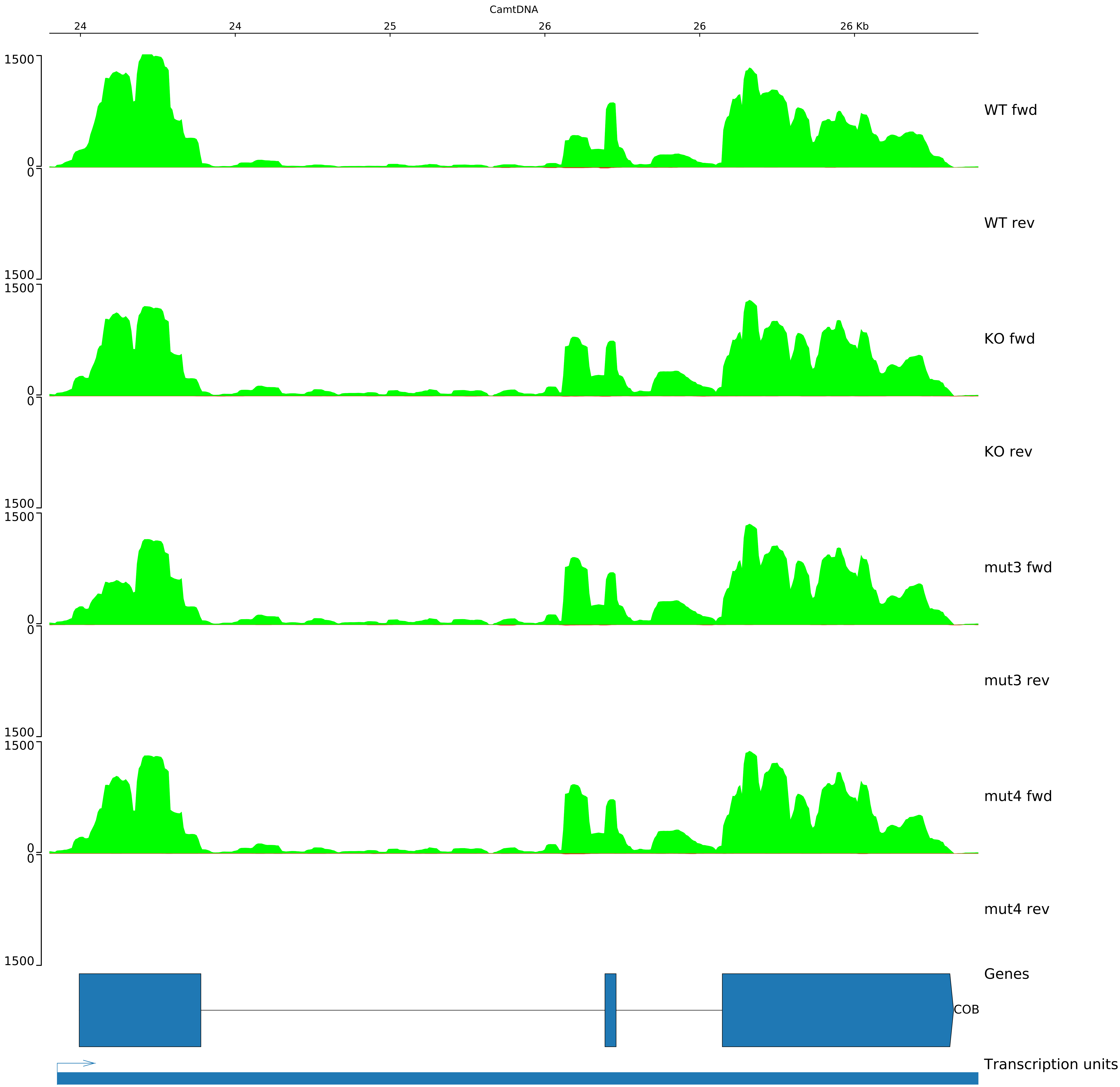

CamtDNA
24
24
25
26
26
26 Kb
1500
0
WT fwd
0
WT rev
1500
1500
0
KO fwd
0
KO rev
1500
1500
0
mut3 fwd
0
mut3 rev
1500
1500
0
mut4 fwd
0
mut4 rev
1500
Genes
COB
Transcription units

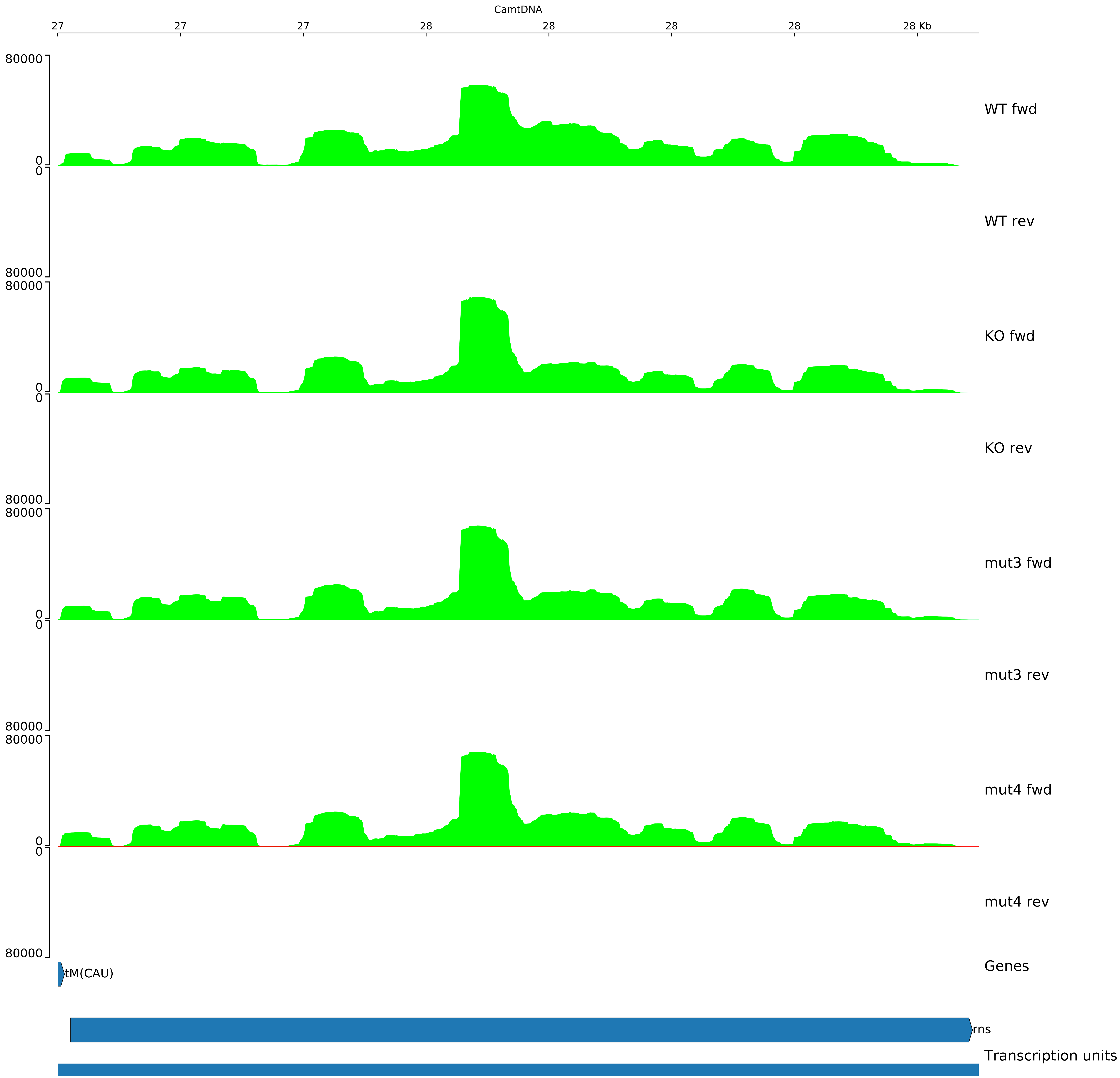

CamtDNA
27
27
27
28
28
28
28
28 Kb
80000
0
WT fwd
WT rev
KO fwd
KO rev
mut3 fwd
mut3 rev
mut4 fwd
mut4 rev
Genes
tM(CAU)
rns
Transcription units

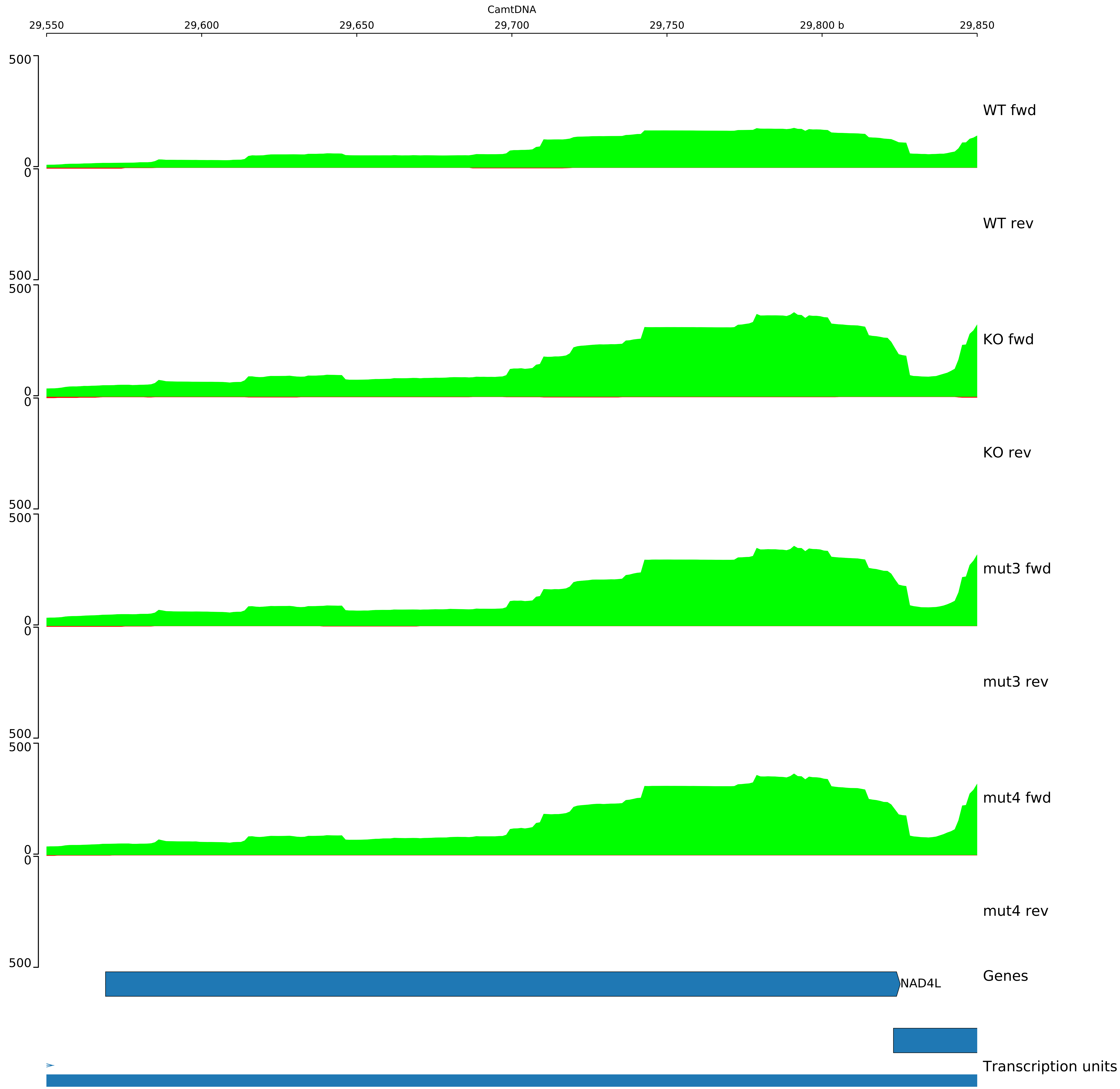

CamtDNA
29,550
29,600
29,650
29,700
29,750
29,800 b
29,850
500
0
WT fwd
WT rev
KO fwd
KO rev
mut3 fwd
mut3 rev
mut4 fwd
mut4 rev
Genes
NAD4L
Transcription units

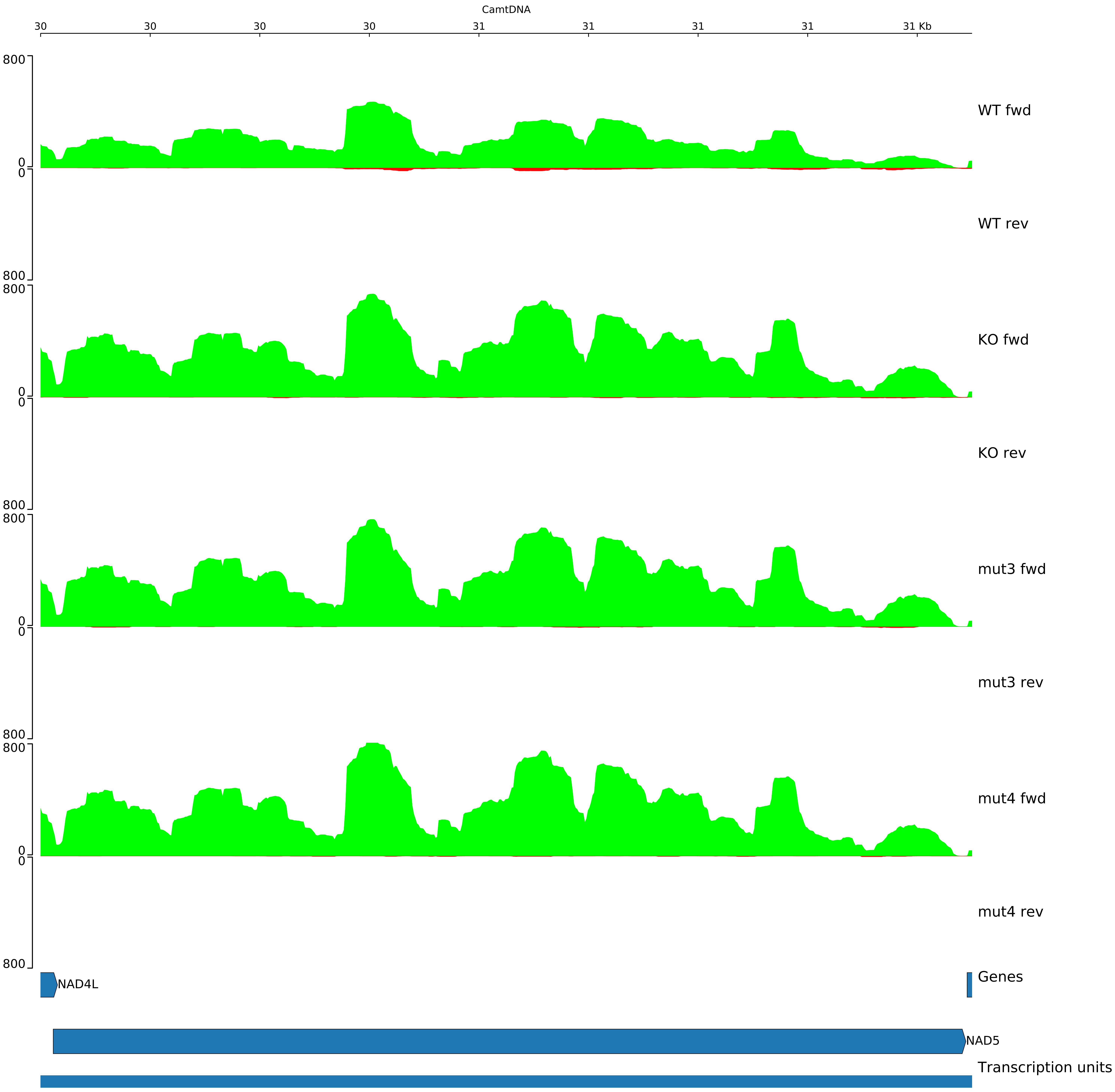

CamtDNA
30
30
30
30
31
31
31
31
31 Kb
800
0
WT fwd
WT rev
KO fwd
KO rev
mut3 fwd
mut3 rev
mut4 fwd
mut4 rev
Genes
NAD4L
NAD5
Transcription units

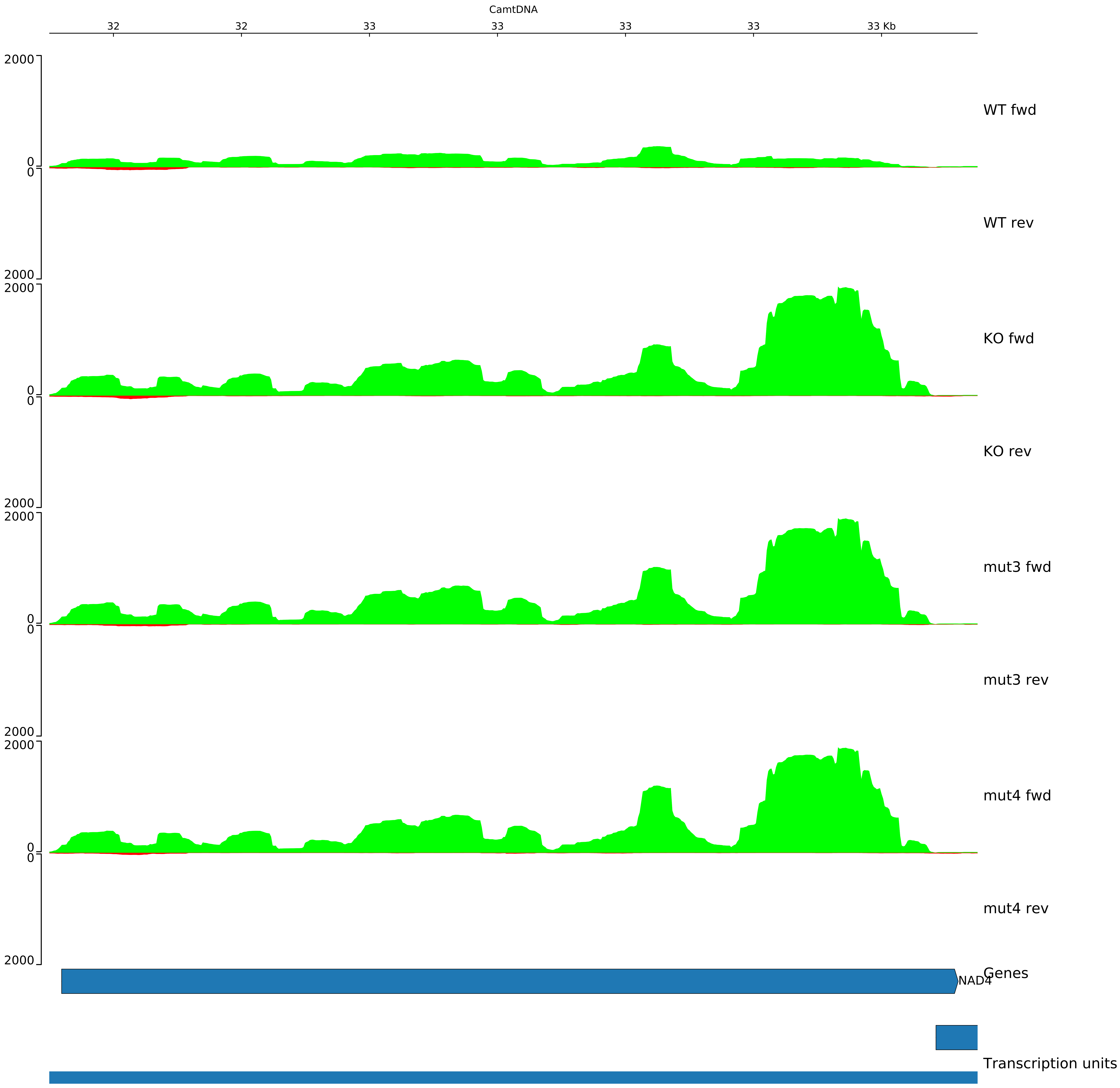

CamtDNA
32
32
33
33
33
33
33 Kb
2000
0
0
2000
2000
0
0
2000
2000
0
0
2000
2000
0
0
2000
WT fwd
WT rev
KO fwd
KO rev
mut3 fwd
mut3 rev
mut4 fwd
mut4 rev
Genes
NAD4
Transcription units
