# Supplementary material for: The Pet127 protein is a mitochondrial 5′-to-3′ exoribonuclease from the PD-(D/E)XK superfamily involved in RNA maturation and intron degradation in yeasts
Source: RNA. 2022 May;28(5):711–28. doi: 10.1261/rna.079083.121 (PMC9014873; doi:10.1261/rna.079083.121)

Supplemental Figure S4. Digestion of 3'-FAM labelled 12 and 40 nucleotide ssRNA substrated in different buffers. Conditions: Buffer 1: 10 mM Tris 7.5, 50 mM KCl, 2 mM MgCl2 , 0.5 mM DTT; Buffer 2: 10 mM Tris 7.5, 150 mM NaCl, 2 mM MgCl2 , 1 mM DTT; 60 nM protein, 30 nM 3’-FAM labelled ssRNA. The size ladder was generated by alkaline lysis of 3’-FAM labelled ssRNA. The faint band migrating faster than the degradation product corresponds to free 3-FAM phosphate, and the shortest band in the alkaline-digested ladder is a cyclic phosphate nucleotide. Counting the faint intermediate bands in the buffer 1 reaction of the 12nt substrate indicates 12 products, showing that the main digestion product is a single nucleotide.

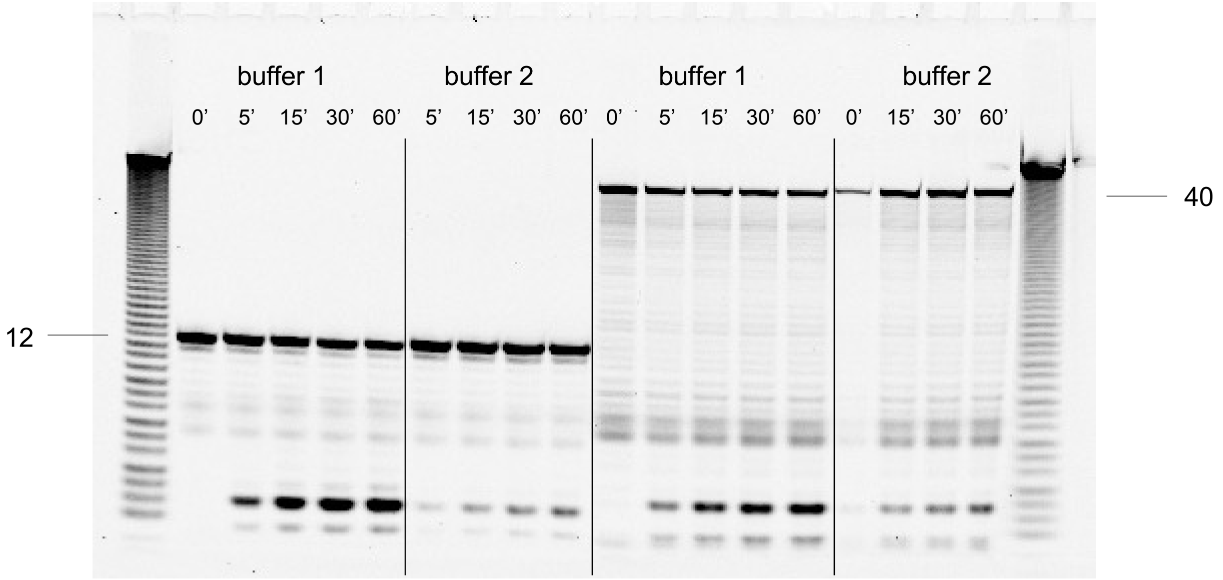

Supplement: Supplemental Material [file supp_079083.121_Supplemental_Figure_S4.pdf]
