# Supplementary material for: The Pet127 protein is a mitochondrial 5′-to-3′ exoribonuclease from the PD-(D/E)XK superfamily involved in RNA maturation and intron degradation in yeasts
Source: RNA. 2022 May;28(5):711–28. doi: 10.1261/rna.079083.121 (PMC9014873; doi:10.1261/rna.079083.121)
Supplement: Supplemental Material [file supp_079083.121_Supplemental_Table_S2.docx]

**Supplemetal Table S2:** List of primers and oligonucleotides used in this work

**A. DNA oligonucleotides**

| Name | Sequence (5’-3’) | Source | Use |
| --- | --- | --- | --- |
| HP1 | CATATCCTCAAGTTAAATATTCCAAATTAGCCGAAATGGAAGCTTCGTACGCTGCAGGTC | This work | Forward starter for  amplification of *CaPET127*  deletion cassette |
| HP2 | AGAGGGGAGACTATTTACAAAATATAGTATGATTTGCTTTTATCTGATATCATCGATGAATTCGAG | This work | Reverse starter for  amplification of *CaPET127*  deletion cassette |
| HP4 | CGCTTGCATTCGTCGTAGTC | This work | Diagnostic PCR |
| HP5 | TCAAGAGAACGAGCAACGCT | This work | Diagnostic PCR |
| HP6 | TCCACACGTGATTCCTAATGGT | This work | Diagnostic PCR |
| HP7 | CATGAACATGAACATCCAATGTC | This work | Diagnostic PCR |
| HP8 | GTAACGCCAGGGTTTTCCCAGTCACGACG  GCTTCACTACTAAAATGTATGCAGGATTAAAGTAAAAATGAG | This work | Forward primer, left flank,  second round of *CaPET127*  deletion |
| HP9 | GACCTGCAGCGTACGAAGCTTCCATTTCGGCTAATTTGGAATATTTAACTTGAGGATATG | This work | Reverse primer, left flank,  second round of *CaPET127*  deletion |
| HP10 | CTCGAATTCATCGATGATATCAGATAAAAGCAAATCATACTATATTTTGTAAATAGTCTCCCCTCT | This work | Forward primer, right  flank, second round of  *CaPET127* deletion |
| HP11 | GCGGATAACAATTTCACACAGGAAACAGC  CTTCATTTGGCCTTACTTGTTGTTGAACAAAACCTTTCAACTC | This work | Reversed primer, right  flank, second round of  *CaPET127* deletion |
| X2SAT | GCACACACTACTTAATATACACAGC | (Walther and  Wendland,  2008) | Diagnostic PCR |
| X3SAT | GTGAAGTGTGAAGGGGGAG | (Walther and  Wendland,  2008) | Diagnostic PCR |
| X2-  CaHIS1 | CAACGAAATGGCCTCCCCTACCACAG | (Walther and  Wendland,  2008) | Diagnostic PCR |
| X3-CaHIS1 | GGACGAATTGAAGAAAGCTGGTGCAACCG | (Walther and  Wendland,  2008) | Diagnostic PCR |
| KL178 | ATTTGCTTTTTATTGAGATCACAGTG | This work | sgDNA |
| KL179 | AAAACACTGTGATCTCAATAAAAAGC | This work | sgDNA |
| KL182 | CTTTTTATTGAGATCACAGTTAGCATGCTATGATGAAAGATTACCAG | This work | Mutagenesis of *CaPET127* |
| KL183 | CTGGTAATCTTTCATCATAGCATGCTAACTGTGATCTCAATAAAAAG | This work | Mutagenesis of *CaPET127* |
| KL184 | CGTGTGATTTAGATACTTTACTTAGTGC | This work | Repair template for pet127_D375A_ |
| KL185 | GCCAGATTTCTAACGAAAACTTAAATTG | This work | Repair template for pet127_D375A_ |
| KL189 | GGAGAAGAAACCAGTCTATT | This work | Diagnostic PCR |
| KL187 | GGTAACTTGTCAGGCAATCTATGTTCATTC | This work | Diagnostic PCR |
| oKD38 | GATATTGCAAGACTAGCTC | This work | Control sequencing of *CaPET127* |
| oKD41 | CAGCGGATCCAGTTTAAAACCAAAACCTGACATC | This work | Cloning CaPET127pET28aSUMO |
| oKD42 | CAGCGGATCCCTATCAAAACTTGAATTTGGGATG | This work | Cloning CaPET127pET28aSUMO |
| oKD43 | ACTCAAAGAATCATACTGTC | This work | Control sequencing of CaPET127pET28aSUMO |
| COB-e1F | GCTATGAGTTATAGGTGTAG | This work | RT-PCR |
| COB-i1F | AGGAATCAGTCTTAGAATATGG | This work | RT-PCR |
| COB-i1R | CTTTAGGACTATCCGCTTG | This work | RT-PCR |
| COB-e2R | GTGTACGGGCAAATGAGTC | This work | RT-PCR |
| RNL-e2F | CAGGTGATTTCTTCAGCATAG | This work | RT-PCR |
| RNL-i2F | CGGGTGGTATTATGGAAG | This work | RT-PCR |
| RNL-i2R | AGCTTACTCACCTTCTAGTC | This work | RT-PCR |
| RNL-e3R | AGTCCTACCCTTCACAGTTG | This work | RT-PCR |

**B. Sequences of RNA oligonucleotides used for Pet127 activity assays.** “FAM” denotes 6-carboxyfluorescein modification of the 3’ or 5’ end of the RNA substrate.

| Oligo name | Sequence |
| --- | --- |
| Sub1 | FAM-AGAGAGUUUGAGAGAGAGAG |
| Sub2 | GGGUAUCAGAUCCCUCGAGAAGCUGCGGGUACC-FAM |
| Sub3 | ACUUAUCAGAUCCCUCGAGAAGCUGCGGGUACC-FAM |
| Sub4 | GGUAGGGCCCACCCGGGAUCUUUGAUCCCGGGUGGGCUAUGUA-FAM |
